# Integration of Motor and Visual Information in the Dentate (lateral) Nucleus of Cerebellum During Locomotion

**DOI:** 10.1007/s12311-026-02088-x

**Published:** 2026-10-24

**Authors:** Irina N. Beloozerova, Wijitha U. Nilaweera, Vladimir Marlinski

**Affiliations:** 1 School of Biological Sciences, Georgia Institute of Technology, 555 14th St., NW, Atlanta, GA 30332 USA; 2 Barrow Neurological Institute, St. Joseph’s Hospital & Medical Center, Phoenix, AZ 85013 USA; 3 Ellsworth Community College, Iowa Falls, IA 50126 USA

**Keywords:** Accuracy, Cat, Firing rate, Neuronal activity, Vision, Walking

## Abstract

The dentate (lateral) nucleus (DN) of the cerebellum is one of the major sources of afferent input to the ventrolateral (VL) thalamus, and further to the motor cortex. Signals conveyed via this pathway are essential for neural control of movements, including accurate vision-guided stepping on complex terrain. The neuronal mechanisms underlying visual control of steps are not well understood. The goal of this study was to elucidate the integration of motor and visual information in the DN during locomotion on complex terrain by characterizing the components of DN activity that reflect overall locomotion movement, components related to visual guidance of feet to desired locations, and the integration of the two. Experiments were conducted on chronically implanted cats using an extracellular single-neuron recording technique. We found that during locomotion on a flat surface, a task that does not require visual control of feet placement, 63% of neurons discharged one activity burst per stride, while 19% had two bursts. Upon transition to vision-guided stepping on crosspieces of a horizontally placed ladder, the firing of 88% of neurons changed: either in the phase (63% neurons), depth of stride-related modulation (59%), firing rate (54%), and/or temporal precision (30%). We describe several ways by which motor and visual information needed for accurate feet placement on complex surface is integrated in the DN.

**Supplementary Information:**

The online version contains supplementary material available at https://doi.org/10.1007/s12311-026-02088-x.

## Introduction

The ability to accurately step on specific sites is critical in daily living. However, neural mechanisms enabling visual guidance of feet to specific locations during locomotion are not well understood. The cerebellum is essential for control of movement accuracy (e.g., [1–9]. This includes control of the accuracy of steps during locomotion [10–13]. The cerebellum receives visual information about the walking path via the pons, and is proposed to be the single most important link between visual and motor areas of the cerebral cortex [14–16]. Signals from the cerebellum are conveyed to the cortex via cerebellar nuclei. The dentate (lateral) nucleus (DN) of the cerebellum is one of the major sources of afferent input to the ventrolateral (VL) thalamus, and further to the motor cortex. During locomotion, the firing activity of neurons in the VL changes periodically in the rhythm of strides [17] and is essential for the stride-related modulation of the activity of neurons in the motor cortex [18–20]. In turn, the activity of neurons in the motor cortex is critical for accurate stepping on visually identified targets [21–27].

During locomotion requiring visual control and accurate stepping, the firing of DN neurons charges vigorously in the rhythm of strides, and it was suggested that this activity helps the accurate feet placement during walking [28]. In the experiments of [28], however, cats only walked along a horizontally placed ladder, a task that requires visual control and step accuracy, while no testing on the flat surface was done, in a task that does not require visual control and particular foot placement,. Thus, it remained unclear which part of the DN activity is specifically related to the visual guidance of feet to desired locations and which simply reflects general locomotion movement.

In this study, we aimed to clarify how DN neurons integrate motor and visual signals in their discharges. We tested the firing activity of DN neurons in cats during locomotion on both a horizontally placed ladder and a flat surface. By comparing the activity of individual neurons and populations of neurons during locomotion on the flat surface and ladder, we isolated and characterized the components of their activity that reflect the overall locomotion movement and those that are specifically related to the visual guidance of feet to crosspieces of the ladder, and described the integration of the two.

## Methods

### Animals and Locomotion Tasks

All procedures followed US NIH guidelines and were approved by the Barrow Neurological Institute Animal Care and Use Committee (protocol #236, assurance #A3519-01). Experiments were conducted in cats as a continuation of related studies of neuronal activity during locomotion in this animal. These include studies of the activity of DN neurons during visually-guided stepping [28] and VL neurons during locomotion on simple and complex terrain [17]. Four adult domestic cats (*Felis catus*) were used: two females, cat JS (weight 2.8 kg) and cat 13 (weight 3.0 kg), and two males, cat 8 (weight 5.0 kg) and cat 14 (weight 3.7 kg). Cats were purchased from a certified class B dealer. Some data obtained from three of these cats were included in previous publications (Appendix).

**Fig. 1:**
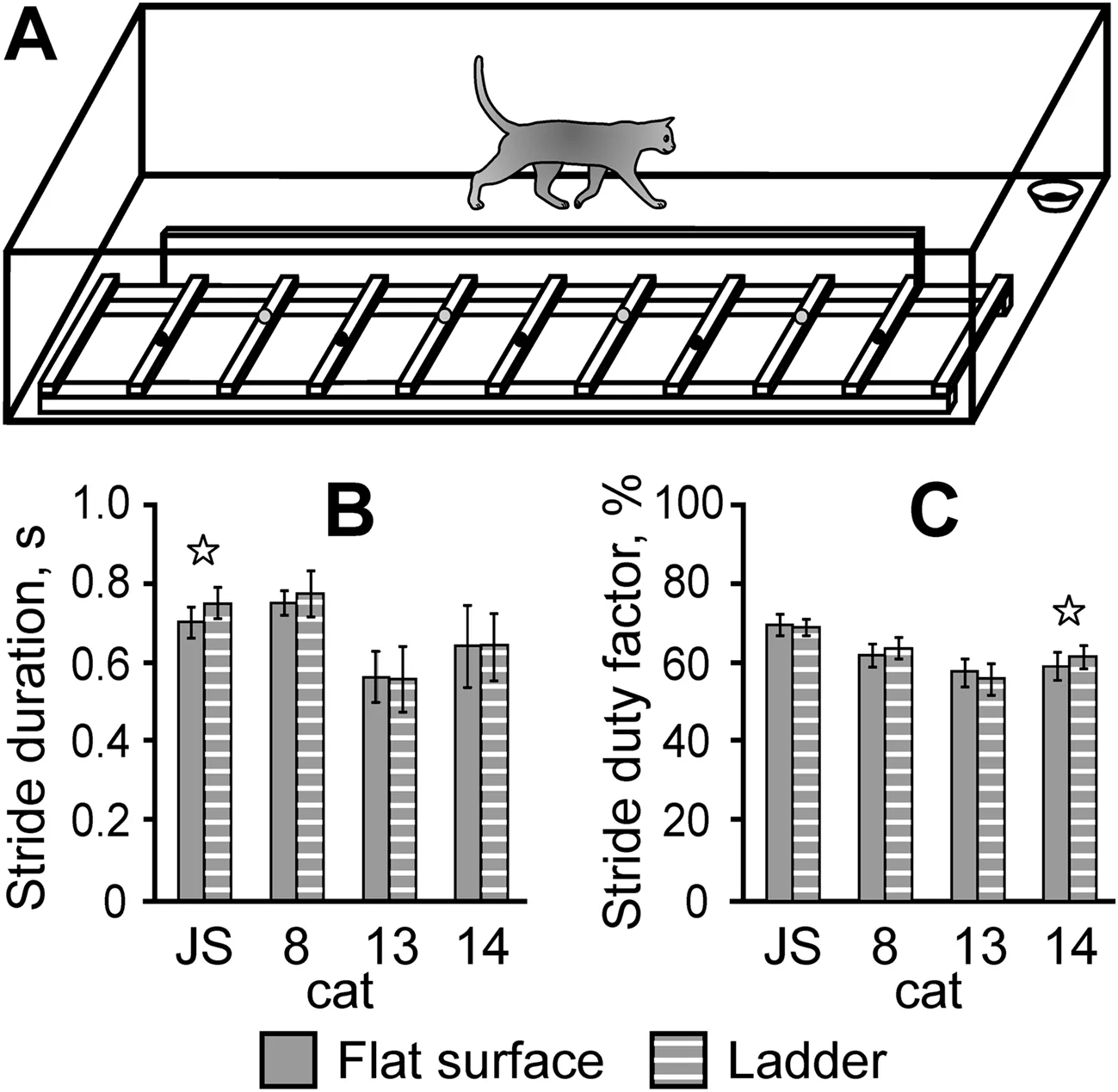
Locomotion tasks. **A**: The experimental box was divided into two corridors. In one corridor, the floor was flat, whereas the other corridor contained a horizontal ladder. Cats walked at self-selected speed. Filled circles on the crosspieces of the ladder schematically show placements of the right (black) and left (gray) paws. See text for description of the ladder. **B**: Stride duration during locomotion on the flat surface and ladder. **C**: Stride duty factor during locomotion on the flat surface and ladder. In B and C, error bars show SDs, stars indicate statistically significant difference between locomotion tasks (*p* =.003 for cat JS stride duration, *p* <.001 for cat 14 stride duty factor, *t* test)

Two tasks were used: locomotion on a flat surface, a task that does not require vision or accurate foot placement, and locomotion on crosspieces of a horizontally placed ladder, a task that requires vision to achieve accurate foot placement (Fig. 1A; [21, 29–33] . Cats were trained using food rewards [34, 35]. They walked in a chamber with two connected parallel corridors 2.5 m by 0.3 m each (Fig. 1A). One had a flat floor; the other contained a "convenient" ladder. The centers of this ladder's crosspieces were spaced 25 cm apart, equal to one-half of a cat’s average stride length during locomotion in the chamber with the flat floor [21, 32]. The crosspieces had flat tops 5 cm wide, which was slightly greater than the 3 cm diameter support area of the cat’s paw (e.g., [36]. During locomotion on the ladder, each limb overstepped two gaps and every other crosspiece of the ladder (Fig. 1A). We have previously shown that when the distance between crosspieces of a horizontal ladder equals half the length of the cat’s normal step and the crosspieces have flat tops just wide enough to fully support the paw, the biomechanics of locomotion on such a "convenient" ladder and flat surface are very close [32]. Namely, 229 variables of full-body mechanics and the activity of eight limb muscles were analyzed, and, besides the variable step size on the flat surface and constant size on the ladder, only a handful of differences were found.

Cats wore a cotton jacket, a light backpack with connectors, and a sock on the right forepaw with a metal plate on the sole for recording paw contact with the rubberized electrically conductive floor and ladder’s crosspieces. Swing and stance of the right forelimb (ipsilateral to DN recordings, see below) were monitored by measuring resistance between the plate and floor. We refer to the full movement cycle of one limb from the beginning of a swing to the beginning of the next swing of the same limb as a step cycle or stride and use these terms interchangeably. During locomotion, cats passed through the two corridors sequentially and repeatedly, occasionally changing direction. During both training and experiments, food was provided in one of the corners after each round. Cats were trained to stand quietly for 3–5 s for food to be dispensed. One second in the middle of this period was considered “standing.”

### Surgical Procedures

The methods of surgical preparation and recording techniques have been described in detail previously [37]. In short, under Isoflurane anesthesia, screws were implanted around the skull and embedded in a plastic cast serving as a base for connectors, micro-drives, and head fixation. On the right side of the head, an array of 1 (cat JS), 3 (cat 8), or 19 (cats 13 and 14) 28-gauge hypodermic guide tubes was implanted in the skull above the DN. The array was aimed at the nucleus, its tip placed just above the surface of the cerebellar cortex without penetrating it. The hypodermic tubes were fitted with stainless steel wires that were later replaced with electrodes for recordings in awake cats. Immediately after the surgery and 12 h thereafter, an analgesic buprenorphine was administered subcutaneously.

### Single−unit Recording and Receptive Field Identification

**Fig. 2:**
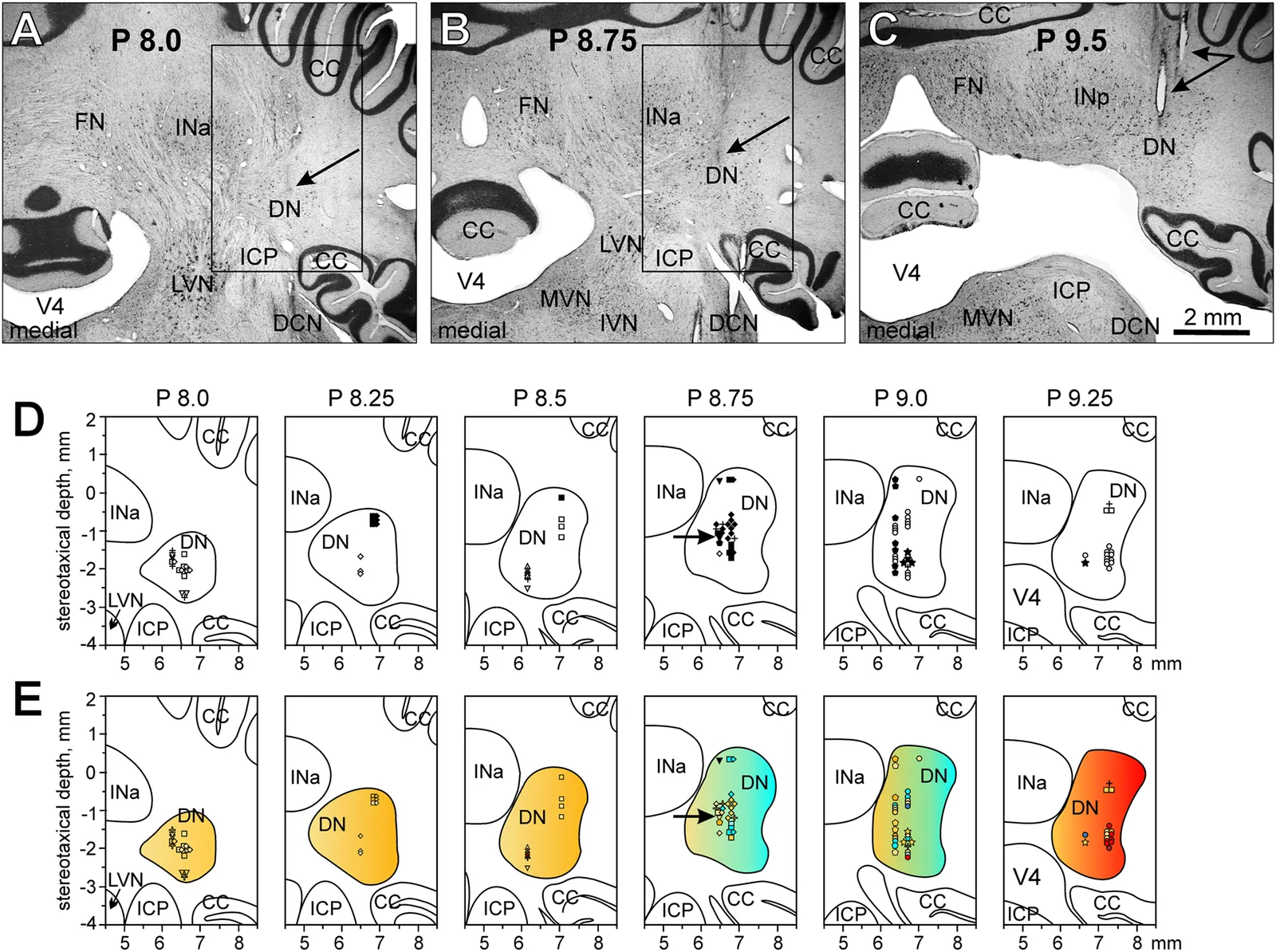
Region of recording in DN and location of neurons. **A-C**: Recording sites in DN of cat 14 are shown on photomicrographs of frontal sections of the cerebellum. **A**: Section at ~ P8.0 rostro-caudal plane. **B**: Section at ~ P8.75 plane. **C**: Section at ~ P9.5 plane. Arrows point to electrolytic lesion marks. In A & B, the black rectangle denotes the area shown in the corresponding panel in D. Cresyl violet stain. **D**: Reconstruction of locations of neurons recorded during locomotion in cat 14. Filled symbols represent neurons with a receptive field (RF) on the forelimb or head; open symbols show those with a RF on the hindlimb. Squares, neurons with a RF on the shoulder (responding to a passive movement in the shoulder joint and/or palpation of muscles on the upper back or neck) or on the hip (responding to a passive movement in the hip joint and/or palpation of muscles on the lower back); diamonds, cells that were activated by movements in the elbow or knee joint; overlapping square and diamond, neurons with a RF encompassing both the shoulder (hip) and elbow (knee); up-facing triangles, cells with a RF on the wrist (ankle) or paw; down-facing triangles, cells whose RF included the entire limb; pentagons, cells activated by palpation of neck muscles, turning of the head, or a touch to the face; stars, cells responsive to visual stimulation or eye movement; circles, cells without RF; crosses, cells whose RF was not tested. **E**: Same as D, but symbols representing neurons are color-coded according to whether visual (red and orange) or motor (blue) information dominated their firing during ladder locomotion. See text for details. Nucleus area is shaded by information type that dominated the output of the majority of neurons in the area. In D, E, an arrow in panels P 8.75 points to an estimated location of the neuron whose firing during locomotion is shown in Fig. 3. CC, cerebellar cortex; DCN, dorsal cochlear nucleus; DN, dentate nucleus; FN, fastigial nucleus; ICP, inferior cerebellar peduncle; INa, anterior interposed nucleus; INp, posterior interposed nucleus; IVN, inferior vestibular nucleus; LVN, lateral vestibular nucleus; MVN, medial vestibular nucleus; V4, fourth ventricle

Extracellular recordings of single neuron activity were obtained from the rostral half of the DN (Figs. 2A-C), the region most heavily projecting to the VL [17, 38, 39]. A tungsten varnish-insulated microelectrode (120 µm, 1–3 MΩ at 1000 Hz; FHC Inc., Bowdoin, ME) was advanced using a lightweight (2.5 g) manual micro-manipulator permanently fixed to the cat’s head. Signals were pre-amplified with a preamplifier on the cat’s head, and further amplified and filtered (0.3–10 kHz) with a CyberAmp 380 (Axon Instruments). After amplification, signals were digitized with a sampling frequency of 30 kHz and recorded using a data acquisition and analysis package (Power1401/Spike2 System, Cambridge Electronic Design, Cambridge, UK). The Power1401/Spike2 waveform-matching algorithm was used to initially identify and isolate spikes of single neurons.

Receptive fields (RFs) of the neurons were examined in the animal sitting with restrained head. Somatosensory stimulation was produced by lightly stroking fur, palpating muscles bellies and tendons, or passive movements around limb joints. Visual stimuli, such as toys, laboratory objects, and hands were moved in different directions in front of the animal at 0.5–1.0 m/s. 

### Processing of Neuronal Activity

Calculations were conducted using a custom MATLAB program (The Mathworks Inc, Natick, MA, USA) or Excel (Microsoft Corporation, Redmond, WA, USA). From the four or five strides along each corridor (Fig. 1A), two or three in the middle were selected for analysis. The strides were further selected so that their average duration during flat surface and ladder locomotion in each session differed by no more than 10%. Selecting strides of similar duration minimized potential differences in the activity of neurons resulting from the difference of locomotion speed between the two tasks. Each group of selected strides contained at least 15 strides.

**Fig. 3:**
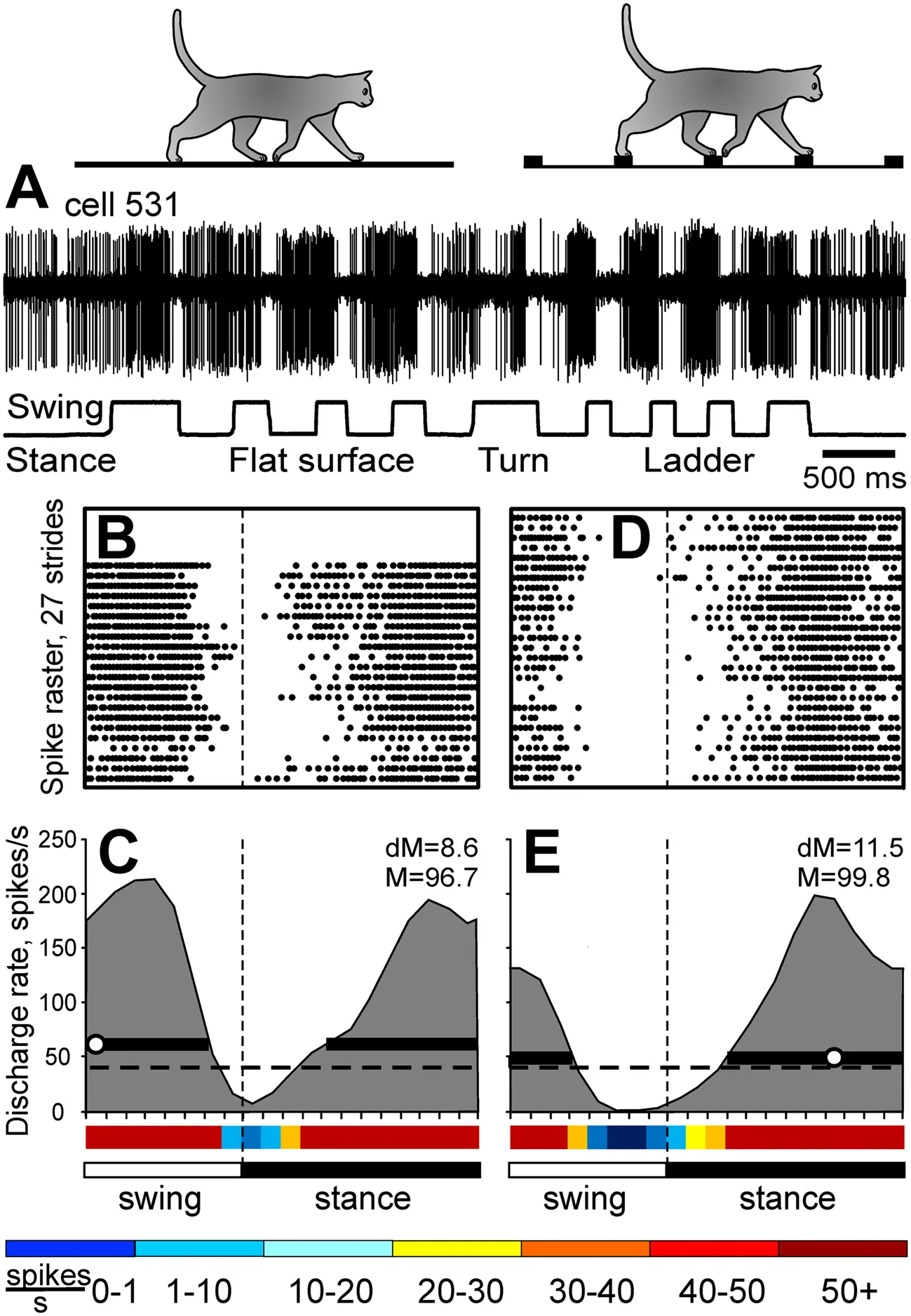
Example activity of a neuron during locomotion on the flat surface and ladder. **A**: Discharges during one round of locomotion. Cat silhouettes at top indicate flat-surface (left and ladder (right) locomotion. Bottom trace shows swing (up) and stance (down) of the right forelimb. **B, C**: Activity on the flat surface shown as a raster of 22 step cycles (B) and a histogram (C). In the histogram, the horizontal dashed line shows the standing firing rate, the horizontal black bar indicates the PEF, and the circle shows the PrPh. The values of the depth of modulation (dM) and coefficient of modulation (M) are indicated. The rainbow bar below the histogram shows the average discharge frequency in each 1/20th portion of the cycle, color-coded according to the scale at bottom of the figure. Periods of the swing and stance phases of the right forelimb are indicated by white and black horizontal bars, respectively. Vertical dashed line denotes the average end of swing and beginning of stance of right forelimb. **D, E**: Activity during ladder locomotion

The onset of the swing phase of the right (ipsilateral) forelimb was taken as the beginning of the step cycle for all neurons with a forelimb-, head/neck, or vision-related RF, as well as for cells without a RF recorded in the vicinity of these neurons. The estimated onset of the swing phase of the right (ipsilateral) hindlimb was taken as the beginning of the step cycle for all neurons with a hindlimb-related RF and for six cells without a RF recorded in the vicinity of these neurons. The estimate was based on data on the cat interlimb coordination during locomotion tasks studied here [32]. The step cycles were time-normalized, and raster plots were created to visualize the discharge of the neurons across all selected step cycles (e.g., Figs. 3B, D). The duration of each step cycle was divided into 20 equal bins, and a phase histogram of the discharge rate during the cycle was generated, averaged over all selected cycles (e.g., Figs. 3C, E). The phase histogram was smoothed by recalculating the value of each bin as: F_n_’ = 0.25F_n − 1_ + 0.5F_n_ + 0.25F_n + 1_, where F_n_ is the original value of a bin.

A coefficient of the stride-related activity modulation, the “depth” of modulation (dM) that characterizes fluctuation in probability of the discharge, was calculated as dM = (N_max_ − N_min_)/N * 100%, where N_max_ and N_min_ are the number of spikes in the maximal and minimal histogram bin, and N is the total number of spikes in the histogram. The activity with dM > 3.6% was judged to be stride-modulated. This threshold was determined by analyzing the activity fluctuations in the standing animal. For this analysis, discharges of 100 neurons recorded while the cat was standing in front of the feeding dish were processed as if the cat was walking [17, 40, 41]. The timing of steps made by the same cat during the preceding walking was used to construct the histogram. This analysis showed that during standing the values of dM exceeded 3.6% in only five cells. Therefore, when the dM was greater than 3.6% during locomotion, we concluded with 95% confidence that this was due to the stride-related modulation. In addition to the dM, a coefficient of the stride-related frequency modulation, M, was calculated using the histogram of cell activity throughout the step cycle. It was defined as M = (1 − F_min_/F_max_) * 100%, where F_min_ and F_max_ are the minimal and maximal frequencies of the discharge in the histogram. The firing of neurons with M > 50% was judged to be stride-modulated based on a threshold estimate similar to that for the dM. The two measures for modulation, dM and M, enabled the characterization of the fluctuation in discharge rate both in terms of the probability (dM) and frequency (M) of discharge.

The portion of the step cycle, in which the discharge rate exceeded the value of the minimal rate plus 25% of the difference between the maximal and minimal rates in the histogram, was defined as a “period of elevated firing” (PEF; Figs. 3C, E). PEFs were smoothed by removing all one-bin peaks and troughs (1.1% of bins were altered throughout the database). For neurons with a single PEF per step cycle, the “preferred phase” (PrPh) of the activity was calculated using circular statistics [30, 31, 42–44]. The coefficient of variability of discharge rate, CV, was defined as CV = σ^2^/m, where σ is standard deviation and m is mean firing rate.

The following parameters of the activity were calculated for each neuron: the mean discharge rate, dM, M, CV, the number of PEFs, the duration of PEF(s), and, for neurons with a single PEF per step cycle, the PrPh. For each neuron, the difference in each activity parameter between locomotion on the flat surface and the ladder was determined. For the comparison of the discharge frequency during different tasks, the Student’s two-tailed *t* test was used. When comparing dMs, Ms, PrPhs, and durations of PEFs, differences equal to or greater than ± 2%, ± 20%, ± 20%, and ± 20%, respectively, were considered significant. These criteria were adopted based on the results of bootstrapping analyses that compared differences in activity parameters between various reshufflings of strides from the same locomotion task [17, 40, 41].

For populations of neurons, the following activity parameters were calculated and compared between locomotion tasks: the percentage of neurons at their PEF during different phases of the stride, the distribution of the average discharge frequency of the population over the stride, the range of coefficients of modulation, and the average widths of PEFs. The difference of each parameter of population activity between two tasks or two groups of neurons was tested using Student’s two-tailed *t* test. Unless noted otherwise, for all mean values the standard error of the mean (SEM) is given. When data were categorical, a nonparametric Mann–Whitney (*U*) test or χ^*2*^ test (Fisher’s two-tailed test) was performed. To compare values among several groups, Welch one-way ANOVA followed by post-hoc *t* tests with Holm-Bonferroni correction was conducted. To determine whether neuronal recruitment fluctuated across the step cycle, a planned contrast within a logistic generalized estimating equations (GEE) model (bin as categorical predictor, neuron as the clustering unit, exchangeable working correlation, robust sandwich SEs) was used (GEE contrast). The contrast compared the equal-weighted mean log-odds of firing across selected bins via a Wald χ^*2*^ test. The level of significance for all tests was set at 0.05. 

### Histological Procedures

At termination, cats were deeply anesthetized with pentobarbital sodium, and reference electrolytic lesions were made in the areas of recording. Animals were perfused with a 4% paraformaldehyde solution, and brains were removed. Frozen 40 μm sections were cut through recording regions and stained with cresyl violet. The positions of the recording tracks in the DN were estimated in relation to reference lesions. See [17] for details.

## Results

### Area of Recording, Neuronal Populations Studied, and Activity During Standing

Recordings were obtained from the rostral half of the DN. This was determined by evaluation of brain sections containing reference lesions in the area of recordings using brain atlases of Reinozo-Suarez [45], Snider and Niemer [46], Berman [47], and Berntson et al. [48]. Figures 2A-C show photomicrographs of three frontal sections of the cerebellum and brainstem of cat 14 with electrolytic lesions visible in the DN at the antero-posterior coordinates of P 8.0 (Fig. 2A), P 8.75 (Fig. 2B), and P 9.5 (Fig. 2C). The activity of 104 neurons was recorded in this cat between P 8.0 and P 9.25. Figure 2D shows their estimated positions within the nucleus. In cat 13, recordings were obtained between P 8.0 and P 9.0 (not illustrated). In cat JS and cat 8, the area of recording was slightly more caudal, P 9.5 and P 9.75, respectively. According to different atlases, the DN may be recognized up to P 10.5—P 11.5.

This report is based on the analyses of the activity of 157 neurons: 104 from cat 14, 32 from cat 13, 15 from cat JS, and 6 from cat 8. These neurons were recorded in 23 microelectrode penetrations: 13 in cat 14, 7 in cat 13, 2 in cat 8 and 1 in cat JS. Between 1 and 19 neurons were recorded per penetration, 6.8 ± 5.3 (mean ± SD) on average.

Responses of 126 neurons to somatosensory and visual stimulation were tested. A third of neurons (37%, 47/126) did not respond. Of the 79 responding, 33 were activated by a movement of the ipsilateral (right) forelimb or palpation of its muscles: of the shoulder or shoulder and elbow ("shoulder & elbow-related" cells, *n* = 10; Supplementary Fig. 1 A), elbow only (*n* = 15), wrist (*n* = 2), or the entire limb (*n* = 6, including 3 cells that responded to either forelimb). In addition, 24 neurons were activated by a movement of the ipsilateral (right) hindlimb or palpation of its muscles: of the hip (*n* = 7), hip and knee (*n* = 4), knee (*n* = 4), paw (*n* = 4), or the entire limb (*n* = 5). Also, 10 neurons were activated by palpation of neck muscles or turning of the head, and 6 responded to a gentle touch on the face. Besides, 5 neurons were activated by visual stimuli (Supplementary Fig. 1B) or eye movement, 3 of these also responded to somatosensory stimulation of the head and body. One neuron responded to vestibular stimulation, a yaw head rotation.

For cat 14, we reconstructed positions of neurons with different RFs within the nucleus (Fig. 2D). Cells with a hindlimb-related field were predominantly located in the most rostral aspect of the nucleus (Fig. 2D, panels P 8.0—P 8.5). Caudal to them were neurons with a forelimb-related field (Fig. 2D, panel P 8.75), and caudal to these were cells with a RF on the neck or head and neurons responsive to visual stimuli or eye movement (Fig. 2D, panels P 9.0—P 9.25). This distribution is analogous to that observed in monkeys [38, 49].

**Fig. 4:**
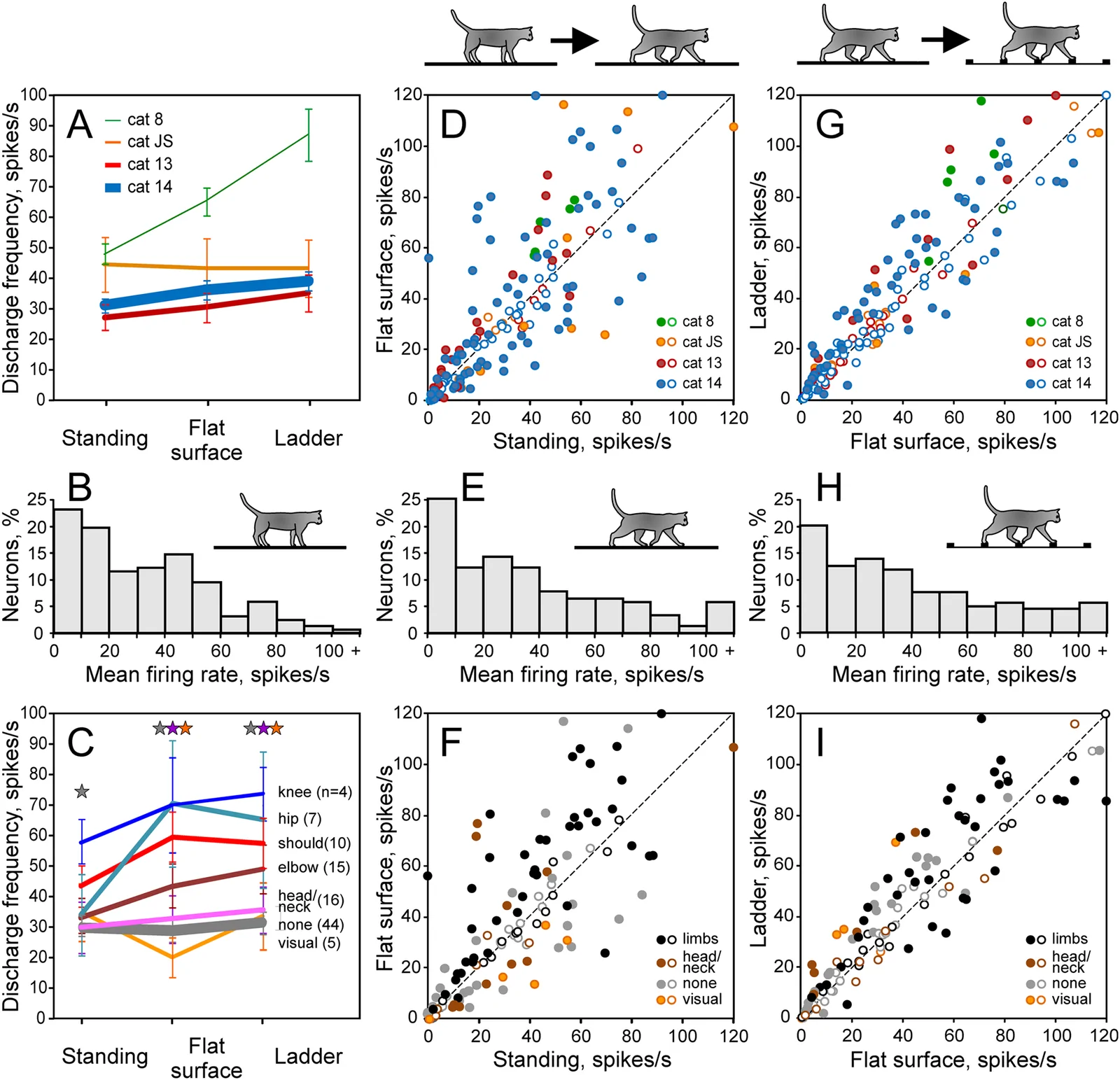
Comparison of neuron firing rates across tasks. **A**: Average firing rate of neurons during different tasks. Data obtained from different cats are color-coded as indicated in the insert. Line thickness reflects the number of neurons in each cat: 104 in cat 14, 32 in cat 13, 15 in cat JS, and 6 in cat 8. Error bars show SEM. **B**: Distribution of mean firing rates during standing. **C**: Average firing rate during different tasks of neurons with different RFs. The general characteristic of the RF is stated next to the colored line. Line thickness reflects the number of neurons in the group (shown in brackets). Stars mark statistically significantly lower firing of the group of cells without a RF (grey stars) or those with a head/neck- or vision-related RF (purple and yellow stars, respectively) compared to the combined group of cells with a limb-related RF. Error bars show SEM. **D**: Comparison of the firing rate of individual neurons during standing and locomotion on the flat surface. The *x*- and *y*-axes of each point show the firing rate of a neuron during standing and locomotion, respectively. Neurons whose firing rate was statistically different between the tasks are shown by filled symbols, others are shown by open symbols. Symbols of different colors show cells recorded in different cats, color-coded as indicated in the insert. **E**: Distribution of mean firing rates during flat-surface locomotion. **F**: Comparison of the firing rate of individual neurons with different RFs during standing and flat-surface locomotion. Symbols of different colors show cells with different RFs, color-coded as indicated in the insert. Other designations as in D. **G**: Comparisons of the firing rate of neurons during flat-surface and ladder locomotion. Designations as in D. **H**: Distribution of mean firing rates during ladder locomotion. **I**: Comparisons of the firing rate of neurons with different RFs during flat-surface and ladder locomotion. Designations as in F

When the cat was standing, all cells except one (156/157) were active. The average firing rate did not differ significantly among cats, equaling: 27.2 ± 4.3, 31.1 ± 2.4, 44.7 ± 9.1, and 48.2 ± 3.5 spikes/s for cats 13, 14, JS, and 8, respectively (*p* = 0.061, *F*(3,153) = 2.51, ANOVA; Fig. 4A, left). The frequency distribution of mean firing rates of all neurons is shown in Fig. 4B. The discharge variability, CV, was 1.48 ± 0.15. Across all cats, cells with a limb-related RF were more active than those without a field, firing 40.6 ± 3.4 *vs*. 30.0 ± 3.5 spikes/s (*p* = 0.033, *t* test; Fig. 4C, left). The firing rate of the fore- and hindlimb-related groups did not differ (*p* = 0.448, *t* test).

### Characteristics of Locomotion

During recording of each neuron, the cat walked between 10 and 50 times (21 ± 9; mean ± SD) down each corridor. A total of 5668 strides were analyzed for the flat-surface locomotion: 479, 299, 1112, and 3778 for cats JS, 8, 13, & 14, respectively, and 4953 were analyzed for the ladder locomotion: 506, 316, 821, and 3310 for cats JS, 8, 13, & 14, respectively. For each neuron, the number of strides selected for analysis according to the criteria described in the Methods was 34 ± 12 for the flat surface and 30 ± 9 for the ladder (mean ± SD). The average duration of strides on the flat surface varied among cats from 571 ± 65 ms in cat 13 to 759 ± 31 ms in cat 8 (Fig. 1B, solid bars). These stride durations corresponded to the locomotion speed of 0.9 m/s for cat 13 and 0.7 m/s for cat 8. The duration of strides on the ladder varied similarly (Fig. 1B, striped bars). For cat JS, strides on the ladder were 48 ms or 6.7% slower than on the flat surface (*p* = 0.003, *t* test), while for all other cats the average duration of strides on the ladder did not differ from that on the flat surface (*p* = 0.431, 0.763, 0.952, for cats 8, 13, 14, respectively, *t* test). The stride duty factor, which is the proportion of the stance phase in the step cycle and characterizes the structure of the stride, varied among cats between 0.58 and 0.7. For cats JS, 8, and 13, the duty factor was similar between the flat-surface and ladder locomotion, whereas for cat 14 it differed by 4.2% (Fig. 1C).

### Population Activity During Vision-independent and Vision-guided Locomotion

A Example neuron activity

When the cat walked on the flat surface, all neurons were active. An example is shown in Figs. 3A-C. In resting cat, this neuron was activated by a touch to the ipsilateral (right) forelimb. In panels P 8.75 of Figs. 2D, E, an arrow points to its estimated position in the nucleus. During standing, the neuron fired 42 spikes/s on average (Supplementary Fig. 1 C). During vision non-requiring locomotion on the flat surface, the discharge rose to 121 spikes/s, and was strongly modulated with the rhythm of strides (Fig. 3A, left side). Figure 3B raster shows higher activity during swing and end-stance. Figure 3C sums firing across 22 strides. The discharge peaked at 214 spikes/s mid-swing and dropped below 10 spikes/s at the swing-to-stance transition. The depth of modulation, dM, was 8.6, and M was 96.7% (see Methods). The period of elevated firing (PEF, black horizontal bar) and preferred phase of the activity (PrPh, white circle) are indicated. During vision-requiring locomotion on the ladder the same neuron fired at 86 spikes/s, 30% lower than on the flat surface. Its discharge now peaked in the second half of stance instead of mid-swing, mostly due to reduced swing activity (Figs. 3A, D, E). The dM was 11.5, higher than on the flat surface, and M was 99.8%. Thus, this neuron's activity illustrates several types of differences seen across the population between vision-independent and vision-requiring locomotion: altered firing rate, altered depth of modulation, and altered phase of firing.

B Mean firing rate and discharge variability

Mean firing rates during flat-surface locomotion did not differ significantly among cats 13, 14, and JS: 30.5 ± 4.9, 35.9 ± 3.1, and 43.3 ± 10.0 spikes/s (*p* = 0.423, *F*(2, 148) = 0.866; ANOVA; Fig. 4A, middle), and for each did not differ from standing (*p* = 0.601, 0.217, 0.921, respectively; *t* test). In cat 8, the rate increased compared to standing (*p* = 0.017, *t* test) and tended to be greater than in other cats (65.4 ± 4.7 spikes/s; *p* = 0.073, *F*(3, 153) = 2.37; ANOVA). The distribution of all mean rates is shown in Fig. 4E. The population average was 36.7 ± 2.5 spikes/s; CV was 2.80 ± 0.17, much higher than during standing (*p* < 0.001, *t* test).

During ladder locomotion, the mean firing rate across cats 13, 14, and JS was 38.9 ± 2.6 spikes/s (Fig. 4A, right). For each of these cats, the rate did not differ from that on the flat surface (*p* = 0.512, 0.459, 0.996, respectively; *t* test). In cat 8, firing rates tended to be higher than on the flat surface (*p* = 0.060, *t* test) and the average was greater than in other cats (87.1 ± 8.7 spikes/s; *p* = 0.005, *F*(3, 153) = 4.46; ANOVA). Figure 4H shows the distribution of mean discharge rates of all neurons. Across all cats, average discharge (40.7 ± 2.7 spikes/s) and CV (2.71 ± 0.14) did not differ from those seen on the flat surface (*p* = 0.271, 0.679, respectively; *t* test).

The firing of somatosensory-responsive cells during flat-surface locomotion was greater than during standing by about 10 spikes/s (48.8 ± 3.9 *vs*. 38.2 ± 3.2 spikes/s; *p* = 0.039, *t* test). These cells were more active than the non-responsive ones, which fired 28.7 ± 4.0 spikes/s (*p* < 0.001, *t* test), and substantially more active than visually-responsive ones, which fired only 19.9 ± 6.5 spikes/s (*p* = 0.006, *t* test; Fig. 4C, middle). Among somatosensory responsive neurons, those driven by limb movement or palpation were more active than cells with head/neck RFs (53.3 ± 4.4 *vs.* 32.8 ± 7.8 spikes/s; *p* = 0.030, *t* test; Fig. 4C, middle). The firing rate of the fore- and hindlimb-related groups did not differ (*p* = 0.576, *t* test). During ladder locomotion, the average firing rate of all neuronal group we considered: the somatosensory- and visually-receptive cells, the highly active limb-related and the low active head/neck-related ones, the fore- and hindlimb-related, the average firing rate did not differ from that seen on the flat surface (*p* = 0.470, 0.337, 0.488, 0.785, 0.568, 0.687, respectively; *t* test; Fig. 4C). Consequently, distinctions in the firing rate seen among these groups on the flat surface remained on the ladder. The somatosensory-responsive cells were still more active than non-responsive ones (*p* = 0.001, *t* test; Fig. 4C, right) and tended to be more active than visually-responsive. Within this group, limb-related cells were more active than head/neck-related (*p* = 0.019, *t* test; Fig. 4C, right).

C Stride-related modulation of the firing

During locomotion on the flat surface, the discharge of 82% (129/157) of neurons was stride phase-modulated, i.e., higher in some stride phase(s) and lower in others. Neurons had either one period of elevated firing (1-PEF) or two (2-PEF). One cell had three PEFs and was grouped with 2-PEF cells. The 1-PEF pattern was more common: 63% (99/157) *vs*. 19% (30/157) with 2-PEF (*p* < 0.001, χ^*2*^ test). The average dM was greater in the 1-PEF than 2-PEF group (8.5 ± 0.4 *vs.* 7.0 ± 0.5, respectively; *p* = 0.029,* t* test), and the PEF duration was longer in the 1-PEF group than the combined duration of PEFs in the 2-PEF one (66 ± 1.5% *vs.* 60 ± 2.0% of the cycle, respectively; *p* = 0.014, *t* test).

**Fig. 5:**
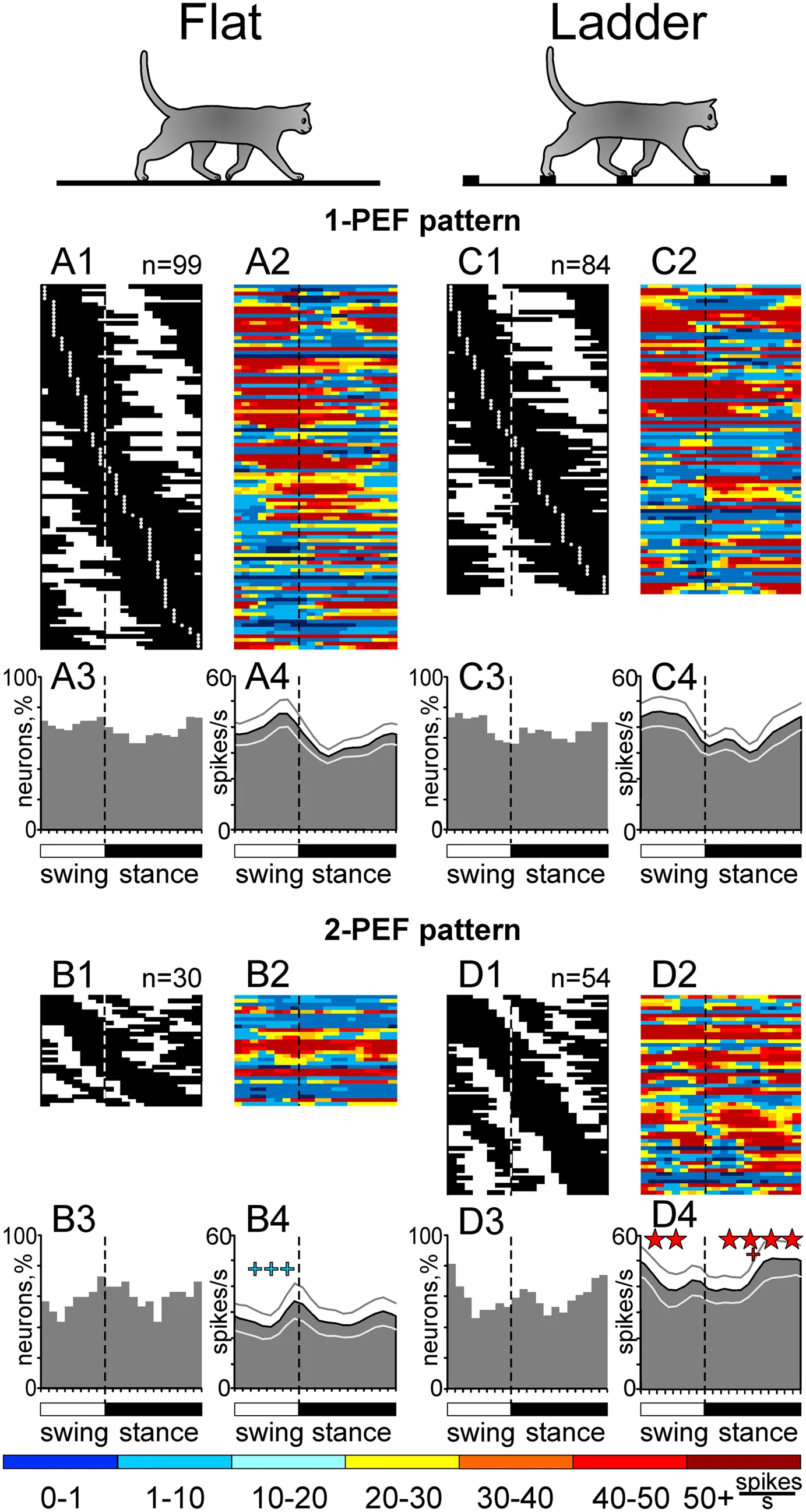
Population characteristics of neurons with 1-PEF and 2-PEF discharge pattern during flat-surface and ladder locomotion. Activity of neurons with 1-PEF (A, C) or 2-PEF (B, D) pattern during locomotion on the flat surface (A, B) and ladder (C, D). **A1—D1**: Phase distributions of PEFs of individual neurons. Each horizontal bar represents the location of PEF(s) of one neuron relative to the step cycle. Neurons are rank-ordered so that those active earlier in the cycle are plotted at top of the graph. In A1 and C1, a white mark indicates the preferred phase (PrPh) for each neuron (see definition in Methods). **A2—D2**: Corresponding distributions of discharge frequencies. The average frequency in each 1/20th portion of the cycle is color-coded according to the scale at bottom of the figure. A3 - D3**:** Fraction of active neurons (neurons at PEF) in different phases of the stride on the flat surface (A3, B3) and ladder (C3, D3). **A4—D4**: Mean discharge during the stride on the flat surface (A4, C4) and ladder (B4,D4). Thin lines show SEM. In B4, blue crosses indicate stride periods of flat-surface locomotion when firing of the 2-PEF group was smaller than that of the 1-PEF group (*p* =.030, *t* test). In D4, the red cross indicates the stride period of ladder locomotion when firing of the 2-PEF group tended to be greater than that of the 1-PEF group (*p* =.057, *t* test). Red stars in D4 show stride periods when the group's firing on the ladder was greater than on the flat surface (*p* =.022, *t* test). In each panel, a vertical dashed line denotes the average end of the right forelimb's swing and the beginning of stance

On the ladder, the discharge of 88% (138/157) of neurons was also stride phase-modulated, a similar fraction to that seen on the flat surface (*p* = 0.189, χ^*2*^ test). These included 16 cells (10%, 16/157) whose firing became stride-modulated only on the ladder. On the other hand, the firing of 6 neurons lost its stride-related modulation upon the flat-to-ladder transition. Discharge of 12 cells (8 recorded in cat JS) was not stride-modulated during either task. Between cats 13 and 14, fractions of cells with and without stride-modulation, as well as fractions with a 1-PEF and 2-PEF pattern did not differ *(p* = 0.069, 0.563, respectively; χ^*2*^ test). The 1-PEF pattern still dominated (54%, 84/157;* p* < 0.001, χ^*2*^ test), but the fraction of cells with a 2-PEF pattern increased to 34% (*versus* 19% on the flat surface;* p* = 0.002, χ^*2*^ test). The firing rate of the 2-PEF group rose compared to the flat surface, by ~ 16 spikes/s (43.9 ± 4.7 *vs*. 27.6 ± 4.7 spikes/s, *p* = 0.017, *t* test; Figs. 5 D4 *vs*. Figure 5B4), while the combined PEF duration still remained shorter than in the 1-PEF group (60 ± 1.5% *vs*. 66 ± 2.0%; *p* = 0.012, *t* test). In addition, the stride-related firing modulation of the 2-PEF group became as high as that of the 1-PEF group (8.6 ± 0.5 *vs.* 8.4 ± 0.4,* p* = 0.755, *t* test).

In both 1-PEF and 2-PEF populations, PEFs of different neurons were distributed across the step cycle and overlapped during either task (Figs. 5A1-D1). On the flat surface, 43–74% of cells were at PEF in every phase (Figs. 5A3, B3). In the 1-PEF group, neuronal recruitment (the percentage of neurons at PEF) was highest (65–74%) during swing and lowest (57%) in early stance (*p* = 0.034, *GEE contrast*; Fig. 5A3). In addition, neurons with a PEF in swing tended to fire more than those with a PEF in stance (42.7 ± 5.3 spikes/s, top of Fig. 5A2, *vs*. 30.7 ± 3.7 spikes/s, bottom of Fig. 5A2; *p* = 0.066, *t* test). Thus, the 1-PEF population rate peaked at the end of swing (at 45.7 ± 5.3 spikes/s, Fig. 5A4).

In the 2-PEF group, recruitment during flat-surface locomotion tended to be higher at swing-to-stance and stance-to-swing transitions and lower mid-swing and mid-stance (*p* = 0.054, *GEE contrast*; Fig. 5B3). The 2-PEF population rate peaked at the swing-to-stance transition (34.2 ± 6.9 spikes/s) with a secondary maximum at the end of stance (30.2 ± 5.5 spikes/s), which is the phase of the swing-to-stance transition of the other (left) forelimb (Fig. 5B4). Through most of swing, the 2-PEF group was 13–19 spikes/s less active than the 1-PEF group (Fig. 5B2,4 *vs.* A2,4).

During ladder locomotion, 45–80% of neurons were also active at any phase (Figs. 5C3, D3). In the 1-PEF group, recruitment peaked (73–76%) during early swing and dipped (56%) at swing-to-stance transition (*p* = 0.007, *GEE contrast*; Fig. 5C3). Neurons with a PEF in swing were more active than those with a PEF in stance (48.9 ± 5.7 spikes/s, top of Fig. 5C2, *vs*. 29.1 ± 4.0 spikes/s, bottom of Fig. 5C2; *p* = 0.006, *t* test). The 1-PEF population's discharge peaked in the first half of swing (at 46.1 ± 5.7 spikes/s), earlier in the cycle than on the flat surface (Fig. 5C4 *vs.* Fig. 5A4). The mid-stance minimum was 35.0 ± 4.9 spikes/s, resulting in a 11 spikes/s range for the group's discharge modulation over the cycle.

Neuronal recruitment in the 2-PEF group during ladder locomotion was similar to that on the flat surface (Fig. 5D3 *vs.* B3). However, unlike on the flat surface, the average discharge of 2-PEF population peaked at stance-to-swing rather than swing-to-stance transition (at 51 ± 6.3 spikes/s; Fig. 5D4). The 2-PEF population was now as active during swing as the 1-PEF group (*p* = 0.684, *t* test; Figs. 5C2,4; D2,4). The discharge rate of the 2-PEF population varied across the step cycle in the range of 12.5 spikes/s.

The characteristics of neuronal group discharges during ladder locomotion were similar to those previously reported by [28].

D Firing patterns of neurons with different receptive fields

The prevalence and strength of stride-related modulation depended on receptive-field of the neurons. During flat-surface locomotion, firing of 91% (71/78) of cells with a somatosensory or visual RF was stride-modulated, a greater fraction than among non-responsive cells (74%, 35/47; *p* = 0.013, χ^*2*^ test). Somatosensory-responsive cells also had higher dM than non-responsive ones (8.8 ± 0.5 *vs*. 7.3 ± 0.5; *p* = 0.026, *t* test). During ladder locomotion, the firing rate of the same fraction of neurons with a somatosensory or visual RF was stride-modulated as on the flat surface (92%, 72/78). However, among non-responsive cells, the firing of 5 more cells became stride-modulated upon the flat-to-ladder transition, and the fraction of such cells in this group became as high as in the responsive cells group (*p* = 0.193, χ^*2*^ test). In addition, the average dM of non-responsive cells did not significantly differ from that of somatosensory-responsive ones anymore (7.9 ± 0.7 *vs.* 9.0 ± 0.4, respectively; *p* = 0.144, *t* test). Thus, the non-responsive group became as involved in locomotion while on the ladder as the cells responsive to somatosensory stimulation, which was not the case on the flat surface.

**Fig. 6:**
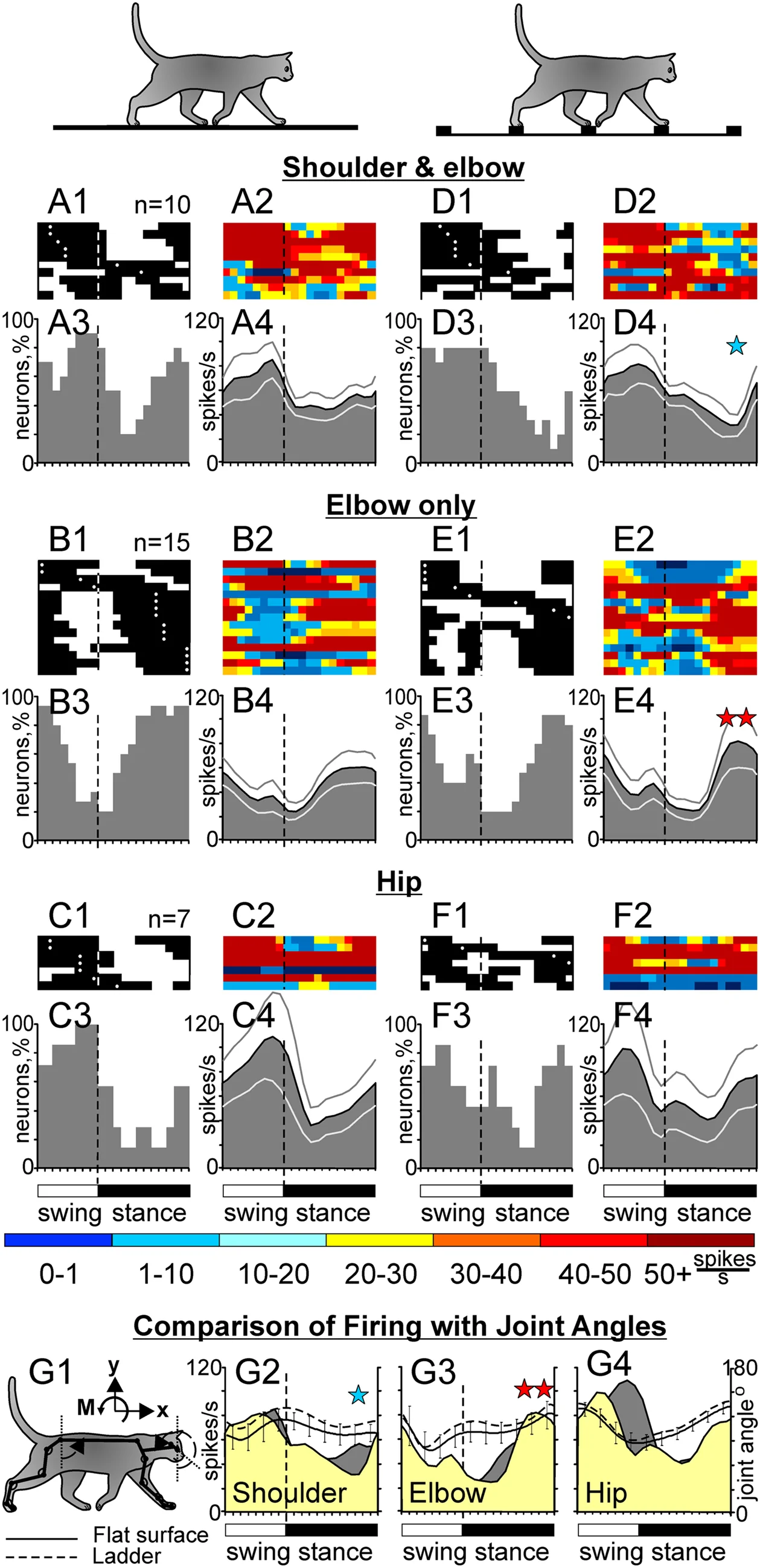
Population characteristics of neurons with a limb-related RF during flat-surface and ladder locomotion. Activity of neurons with shoulder & elbow- (A, D), elbow only- (B, E), or hip-related (C, F) RF during flat-surface (A, B, C) and ladder (D, E, F) locomotion. **A1- F1**: Phase distributions of PEFs of individual neurons. Cells with 1-PEF and 2-PEF pattern are rank-ordered separately. 1-PEF neurons are at top of the graph; a white mark indicates each neuron's preferred phase (PrPh). 2-PEFs are at bottom. Within each group, cells firing earlier in the cycle are plotted at top of the group. **A2—F2**: Corresponding distributions of discharge frequencies. **A3—F3**: Fraction of active neurons (neurons at PEF) in different phases of the stride on the flat surface (A3, B3, C3) and ladder (D3, E3, F3). **A4—F4**: Mean discharge rate during the stride on the flat surface (A4, B4, C4) and ladder (D4, E4, F4). **G1- G4**: Comparison of the firing activity with limb joint angles excursions during flat-surface and ladder locomotion. **G1**: Definition of joint angles. Orientation of each limb was determined as the angle between the negative direction of the vertical axis and the longitudinal segment axis directed from the distal end of the segment to the proximal end, as indicated for the head/neck segment. **G2**: Comparison of the firing activity of the shoulder & elbow-related cell group during flat-surface and ladder locomotion (grey and yellow area histograms, respectively, replotted from A4,D4) with angular excursions in the shoulder joint during flat-surface (solid line) and ladder locomotion (interrupted line). SDs for joint angles were similar between the tasks and for clarity are shown only for flat-surface locomotion. **G3**: Same for the elbow only-related cell group and the elbow joint. **G4**: Same for the hip-related cell group and the hip joint. In D4, E4 and G2,G3: the blue star indicates the stride period when the firing on the ladder was lower than on the flat surface(*p* =.048, *t* test), and red stars show when it was higher (*p* =.009, *U* test). In G2-4,** j**oint angles shown were obtained as part of [32] study by averaging data from five cats. They are described and discussed in that paper but are not illustrated there. Other designations as in Fig. 5

Among forelimb-related neurons, stride phase relationship of the activity differed between groups with more proximal and more distal RFs. During flat-surface locomotion, recruitment of neurons with an RF on the shoulder or shoulder & elbow grew toward late stance and was high in swing, peaking at 90% at end-swing (*p* = 0.002, *GEE contrast*; Figs. 6A1,3). Correspondingly, the firing of shoulder-related cells peaked in swing at ~ 85 spikes/s and dipped to 45 spikes/s during stance (*p* = 0.008, *U* test; Figs. 6A2,4; G2), in synchrony with shoulder's joint excursion (Fig. 6G1,2, solid line in G2). On the ladder, recruitment of these neurons still peaked in swing (~ 80%; *p* = 0.001, *GEE contrast*; Figs. 6D1,3) and dipped to 10–50% in the second half of stance(Figs. 6D1,3). Correspondingly, their firing peaked in swing at ~ 80 spikes/s while dropping to ~ 30 spikes/s in the second half of stance, lower than on the flat surface (*p* = 0.048, *t* test; Figs. 6D2,4 *vs*. A2,4). This drop did not correspond to any change in the shoulder joint excursion (Fig. 6G2, interrupted line in G2).

The strictly elbow-related neurons showed a different stride phase preference. During flat-surface locomotion, they were recruited mostly (at 93%) in the second half of stance and early swing (*p* = 0.002, *GEE contrast*; Figs. 6B1,3). Their firing peaked at ~ 60 spikes/s in the second half of stance and was only ~ 25 spikes/s in early stance (*p* = 0.002, *U* test; Figs. 6B2,4), partly reflecting elbow joint excursion (Fig. 6G3). On the ladder, the recruitment of these cells still peaked in the second half of stance and early swing (at 87%; *p* < 0.001, *GEE contrast*; Figs. 6E1,3). However, their group firing now reached ~ 80 spikes/s in the second half of stance, ~ 20 spikes/s higher than on the flat surface (*p* = 0.012, *U* test; Figs. 6E2,4; G3). The average PEF(s) duration was shorter than on the flat surface (53 ± 3% *vs*. 66 ± 2.5%; *p* = 0.003, *t* test). These changes did not correspond to any change in the elbow joint excursion (Fig. 6G3).

The recruitment of hip-related neurons and their firing rate modulation on the flat surface resembled those of the shoulder-related cells (Figs. 6C1-4; G4), and the knee-related activity resembled hip-related (not illustrated). The average dM and the width of PEF(s) did not differ between hindlimb- and forelimb-related subpopulations (*p* = 0.695, 0.223, respectively; *t* test). During ladder locomotion, the recruitment and firing rate modulation of the hip-related group remained similar to those seen on the flat surface (Fig. 6F1-4), and hip joint excursions were also similar between the tasks (Fig. 6G4). The firing rate modulation of the group of four knee-related cells was, however, markedly different from that on the flat surface, their discharge now peaking in the stance-to-swing transition rather than in swing (not illustrated). On the ladder, the average PEF(s) duration was greater in the hindlimb- than forelimb-related group (63 ± 3.0% *vs*. 53 ± 2.5%; *p* = 0.019, *t* test), while the average dM tended to be smaller (8.1 ± 0.7 *vs*. 10.2 ± 0.6; *p* = 0.063, *t* test). This hindlimb/forelimb distinction was not seen on the flat surface.

**Fig. 7:**
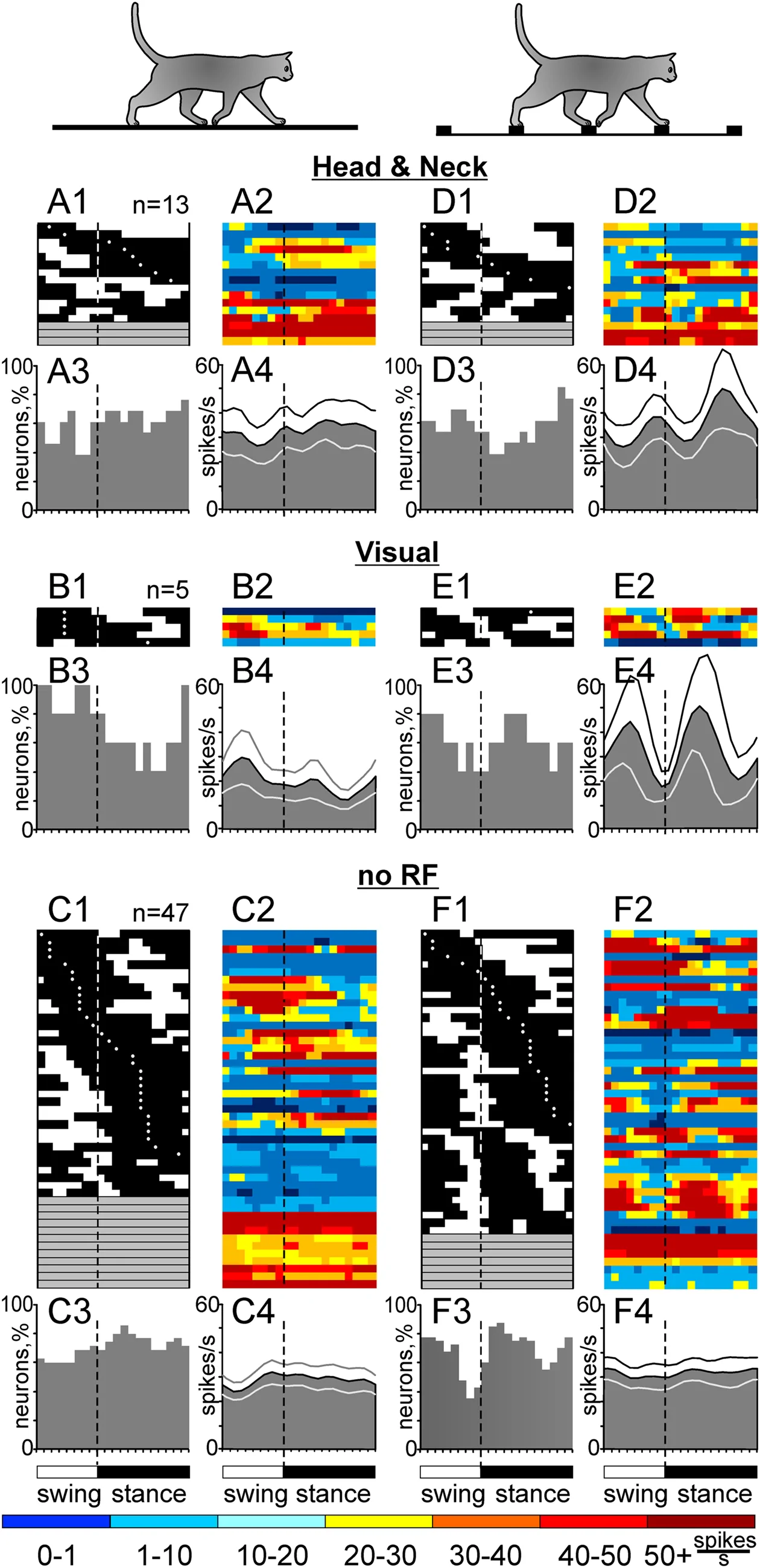
Population characteristics of neurons with a head/neck-related RF, visually-responsive, and non-responsive cells during flat-surface and ladder locomotion. Activity of neurons with a head & neck RF (A, D), vision-related (B, E) RF, and those without a somatosensory or visual RF (C, F) during flat-surface (A, B, C) and ladder (D, E, F) locomotion. **A1- F1**: Phase distributions of PEFs. Gray bars designate cells whose firing was not stride-modulated. **A2—F2**: Corresponding distributions of discharge frequencies. **A3—F3**: Fraction of active neurons in different phases of the stride on the flat surface (A3, B3, C3) and ladder (D3, E3, F3). **A4—F4**: Mean discharge rate during the stride on the flat surface (A4, B4, C4) and ladder (D4, E4, F4). Other designations as in Fig. 5

Unlike the conspicuous stride-modulated firing of the limb-related groups during flat-surface locomotion, the firing of the head & neck-related group was weakly modulated during this task, only changing from ~ 27 spikes/s in swing to ~ 37 spikes/s in stance (*p* = 0.008, *U* test; Fig. 7A4). On the ladder, however, their firing, although still lower than that of the limb-related group (*p* = 0.019, *t* test), became prominently stride-modulated. It showed two peaks: a higher one in the second half of stance (~ 50 spikes/s) and a lower one at end-swing (~ 40 spikes/s;* p* = 0.008,* U* test for the inter-peak difference; Fig. 7D4). This may have led to the downward rotation of the head & neck segment while on the ladder (Figs. 4 F, H & 5 A, B in [32]).

During flat-surface locomotion, the group activity of neurons with a visual RF fluctuated in roughly opposite phase to that of the head & neck-related group, with a 30 spikes/s maximum in mid-swing and a 12 spikes/s minimum in second half of stance (*p* = 0.012, *U* test; Fig. 7B4). There was also a secondary 21 spikes/s maximum in the first half of stance (*p* = 0.016, *U* test; Fig. 7B4), the swing phase of the other (left, contralateral) forelimb. On the ladder, the firing of this group also became prominently stride-modulated, and also with two peaks: one in mid-swing (~ 44 spikes/s) and one in mid-stance (~ 50 spikes/s; Fig. 7E4). They were, as on the flat surface, in roughly opposite phases to the peaks of the head & neck-related group, but now there were two peaks per stride rather than one.

On the flat surface, the group firing of cells without a somatosensory or visual RF had a weak maximum at the swing-to-stance transition (*p* = 0.012, *U* test; Fig. 7C4). On the ladder, their recruitment peaked twice per cycle, roughly synchronously with the visually-responsive cells (Figs. 7F1, 3), however, the average firing rate of this population was steady over the cycle (Figs. 7F2, 4).

In summary, during locomotion on the flat surface, where visual control and step accuracy were not required, the firing activity of the great majority of neurons was stride-modulated. The firing of the 1-PEF population peaked at end-swing of the ipsilateral limb, whereas that of the 2-PEF group peaked at end-swing of either forelimb. The group activities of the shoulder-, hip-, and knee-related neurons were maximal during swing of the ipsilateral limb, while that of elbow-related cells peaked during stance. The firing activity of visually responsive neurons was lower than that of other groups and peaked in mid-swing of either forelimb. Collectively, these were the signals sent from the DN population during unobstructed vision-independent locomotion.

During ladder locomotion, when visual control and step accuracy were required, the firing of the 1-PEF population peaked earlier in the swing phase than on the flat surface. The fraction of neurons with a 2-PEF pattern and their firing rate were greater, particularly during the stance-to-swing transition. PEF(s) in the forelimb-related group were shorter, i.e., more stride-phase specific, than PEF(s) in the hindlimb-related group, and the average dM of the forelimb-related cells tended to be greater, the forelimb/hindlimb differences not seen on the flat surface. Head/neck- and vision-responsive groups developed two activity peaks per stride; these peaks being roughly in opposite phases between the groups. The non-responsive cell group became as involved in locomotion as the other groups. Collectively, these were the signals sent from DN neuronal populations to assist in the accurate placement of feet on the ladder's crosspieces under visual guidance.

### Responses of Individual Neurons to the Ladder Task: the Intensity, Salience, and Precision of the Signals

We next analyzed signals sent by individual neurons. With the start of locomotion, the firing rate of 70% of the neurons changed. In Fig. 4D, the mean discharge rate of individual neurons during flat-surface locomotion is plotted against that during standing. In cat 13 (red symbols), the activity of 59% (19/32) of cells increased, by 84 ± 52% (mean ± SD); in cat JS (orange), the firing of 53% (8/15) of cells decreased, by 39 ± 17%; whereas in cat 14 (blue) the firing of 1/3 of cells increased, while that of 1/3 decreased. Across cats, discharge of most limb-receptive neurons (65%, 37/57) increased, by 62 ± 57%; while that of all visually receptive cells decreased, by 46 ± 18% (Fig. 4F, black & orange). The firing of head & neck-receptive and non-responsive cells did not show a preference to increase or decrease (Fig. 4F, brown & gray).

Upon transition from the flat-surface to ladder locomotion, discharge of 88% (138/157) of cells altered in at least one of five parameters we considered: the firing rate, magnitude of stride-related modulation, duration of PEF(s), number of PEFs, and, for 1-PEF neurons, the preferred firing phase.

The firing rate of half of individual neurons (51%, 80/157; Fig. 4G) changed on the flat-to-ladder transition. In cat 14 (blue), the firing of 41% of neurons increased, by 67 ± 69%, while decreasing in 13%, by 31 ± 18% (mean ± SD). In cat 13 (red), discharge of 34% of neurons increased, about same as in cat 14. In cat 8 (green), firing of all but one neuron increased, by 42 ± 23%, while in cat JS (orange) it changed in only a few cells. Overall, more neurons increased firing than decreased (*p* < 0.001, χ^*2*^ test), however, as described above (Fig. 4A), this did not significantly change the average discharge rate of the entire population.

Among neurons with different RFs, changes in the firing rate differed only slightly. The firing of 3/5 visually responsive cells increased on the ladder compared to flat surface. Among cells not responsive to visual stimulation, the limb-related group tended to have a larger fraction of cells (63%, 36/57) whose firing rate changed upon the flat-to-ladder transition than the head & neck-related and non-responsive groups, where the firing rate of only 38% (6/16) and 45% (21/47) of neurons, respectively, altered (both *p* ≤ 0.067, χ^*2*^ test; Fig. 4I). The limb-related group also tended to have a larger fraction of cells whose firing decreased rather than increased upon the transition, 19% (11/57) compared to 6% (1/16) and 9% (3/47) in the head & neck- and non-responsive groups, respectively. Across all groups, firing increases ranged 10—250% and decreases 10—72%.

**Fig. 8:**
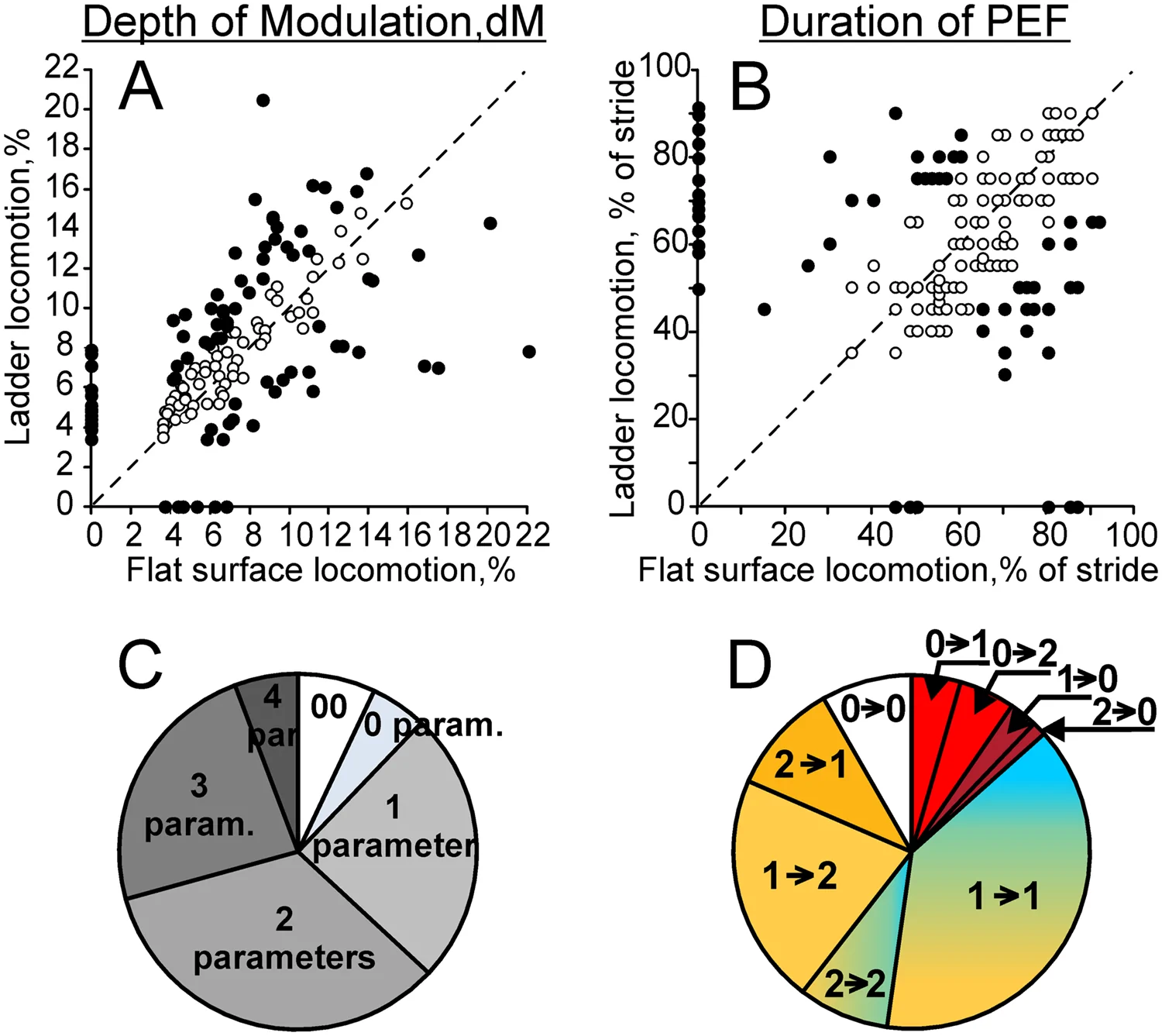
Differences in firing characteristics of individual neurons between flat-surface and ladder locomotion. **A**: Comparison of the depth of stride-related modulation (dM) of neurons. The *x*- and *y*-axes of each point show the dM of the activity of a neuron during flat-surface and ladder locomotion, respectively. Neurons whose dM was statistically different between the tasks are shown by filled symbols, others are shown by open symbols. **B**: Comparison of the PEF(s) duration. For 2-PEF neurons, the combined duration of two PEFs is given. Designations as in A. **C**: Fractions of neurons with different number of activity parameters changing between the two locomotion tasks. 00—cells whose firing was not stride-modulated during either task. 0—cells whose firing was stride-modulated and similar between the tasks. 1–4 parameters—cells, in the activity of which 1, 2, 3, or 4 characteristics were different between the tasks. **D**: Fractions of neurons with similar or different number of PEFs during flat-surface and ladder locomotion: 0, no stride-related modulation; 1, 1-PEF pattern; 2, 2-PEF pattern. The value on the left indicates the number of PEFs during flat-surface locomotion; the value on the right shows that for ladder locomotion

The dM coefficient, which quantifies the fluctuation in the discharge probability over the step cycle and is one of the parameters that characterize the salience of the locomotion-related signal, was greater on the ladder compared to flat surface in 33% (40/123) of cells with stride-modulated firing during both tasks, while that of 20% (24/123, a smaller fraction; *p* = 0.019, χ^*2*^ test) was smaller (Fig. 8A). Increases in the dM ranged 20–120% (52 ± 27%, mean ± SD); decreases were 37 ± 13%. Increases in the M, a parameter that quantifies the fluctuation in the discharge frequency, were 19.7 ± 6.4% (mean ± SD), and decreases were similar. In addition, the firing of 15 neurons was stride-modulated only on the ladder, while that of 6 was modulated only on the flat surface (Fig. 8A). Thus, during ladder locomotion the salience, and, therefore, the presumed impact of signals from 66% ([15 + 40]/144) of locomotion-related neurons was greater than on the flat surface while that of only 21% ([6 + 24]/144) was smaller. A reduction in the signal's salience in a subset of neurons was probably needed for accurate stepping on the ladder, along with an increase in the signal's salience in the majority of cells. Similar fractions of neurons with increasing and decreasing dM on the flat-to-ladder transition were previously reported for the cat primary motor cortex [21].

PEF duration characterizes signal's precision. Among 123 cells with stride-modulated firing during both locomotion tasks, PEF(s) shortened on the ladder compared to flat surface in 16% (20/123) of neurons, and lengthened in 14% (17/123; Fig. 8B), with changes being 15–50% of the cycle in either direction. Thus, the signals from individual DN neurons for vision-guided foot placement were not always shorter, more stride-phase precise, but could be longer, less precise, compared to those sent during flat-surface locomotion. This is similar to responses of neurons in the VL thalamus [17].

For most neurons with stride-modulated firing during both tasks (78/123), the stride phase, in which the signal for accurate stepping on the ladder went out, was different from the phase when the neuron fired during the flat-surface locomotion. Below, we analyze these phase differences in detail.

Across the five characteristics of the signal — firing rate (magnitude), depth of modulation (salience), width of PEF(s) (precision), phase, and number of PEFs — the majority of neurons (59%, 92/157) had only one or two different between the tasks (Fig. 8C, light grey). Most often it was a difference in the firing rate (*n* = 13) or in the depth of modulation plus the number of PEFs (*n* = 13), or in the firing rate plus the number of PEFs (*n* = 11). Ten cells had a different number of PEFs only. The activity of 29% (46/157) of neurons differed in three or four parameters (Fig. 8C, dark gray), most often in the firing rate, depth of modulation, and number of PEFs (*n* = 16) or the firing rate, depth of modulation, and width of PEF(s) (*n* = 13).

### Integration of Motor and Visual Information in the Activity of Individual Neurons

**Fig. 9:**
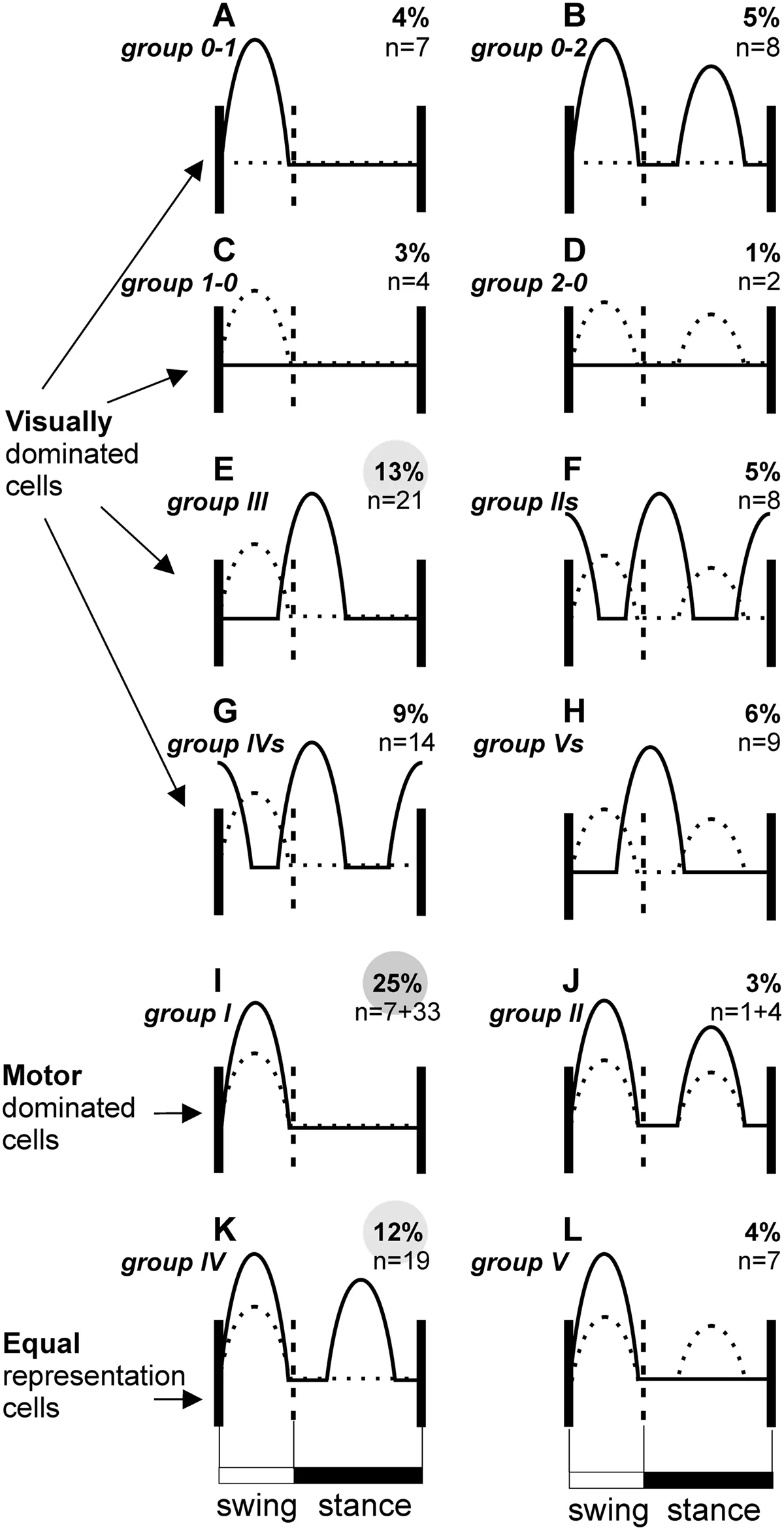
Schematic presentation of changes in the discharge patterns of neurons on the flat-to-ladder transition—the modes of integration of motor and visual information in the activity of individual neurons. Dashed lines show firing during flat-surface locomotion, and solid lines show that for ladder locomotion. **A—H**: Neurons whose activity during ladder locomotion was dominated by visual input. **A, B**—*group 0–1* (A) and *group 0–2* (B) cells with the firing unrelated to strides on the flat surface but stride-modulated with one (*group 01*) or two (*group 02*) PEFs on the ladder. **C, D**—*group 1–0* (C) and *group 2–0* (D) cells with the firing stride-modulated on the flat surface, showing one (*group 1–0*) or two (*group 2–0*) PEFs, but not stride-modulated on the ladder. **E, F**—*group III* (E) and group *IIs* (F) cells with 1-PEF (*group III*) or 2-PEF (*group IIs*) pattern on the flat surface that remained on the ladder, but the PEF(s) occurred in different phase(s) of the stride. **G, H**—*group IVs* (G) and *group Vs* (H) cells with 1-PEF (*group IVs*) or 2-PEF (*group Vs*) pattern on the flat surface that on the ladder had a different number of PEFs, and those PEF(s) were in different stride phase(s) than on the flat surface. **I, J**: Neurons whose discharge pattern was determined by motor information during both tasks, while the magnitude of modulation could be adjusted by visual information while on the ladder. *Group I* (I) and *group II* (J) cells with 1-PEF (*group I*) or 2-PEF (*group II*) pattern on the flat surface that remained on the ladder, and the PEF(s) phases did not change. In seven 1-PEF and one 2-PEF neurons, none of the five evaluated activity parameters changed on the flat-to ladder transition. **K, L:** Neurons, to the activity of which on the ladder, visual and motor information contributed approximately equally. *Group IV* (K) and *group V* (L) cells with 1-PEF (*group IV*) or 2-PEF (*group V*) pattern on the flat surface that on the ladder had a different number of PEFs, but those PEF(s) occurred in one of the same phase(s). The number and fraction of neurons in each group are stated on each panel. *Group I* (*n* = 40, 25%) was the most populous, followed by the distant second and third *groups III* and *IV* (13% and 12%, respectively); fractions of these three groups are highlighted by a gray circle

Changes in the discharge of individual neurons on the flat-to-ladder transition reflect, at the single-neuron level, integration of signals related to processing of visual information for accurate stepping with signals related to basic motor aspects of locomotion [17, 50]. We identified 12 types of integration, grouped into three broad categories: (i) domination of vision-related signals, (ii) domination of motor-related signals, and (iii) equal contribution of both (Fig. 9).

A Neurons with stride-modulated firing only during ladder locomotion, *groups 0–1* and *0–2*

**Fig. 10:**
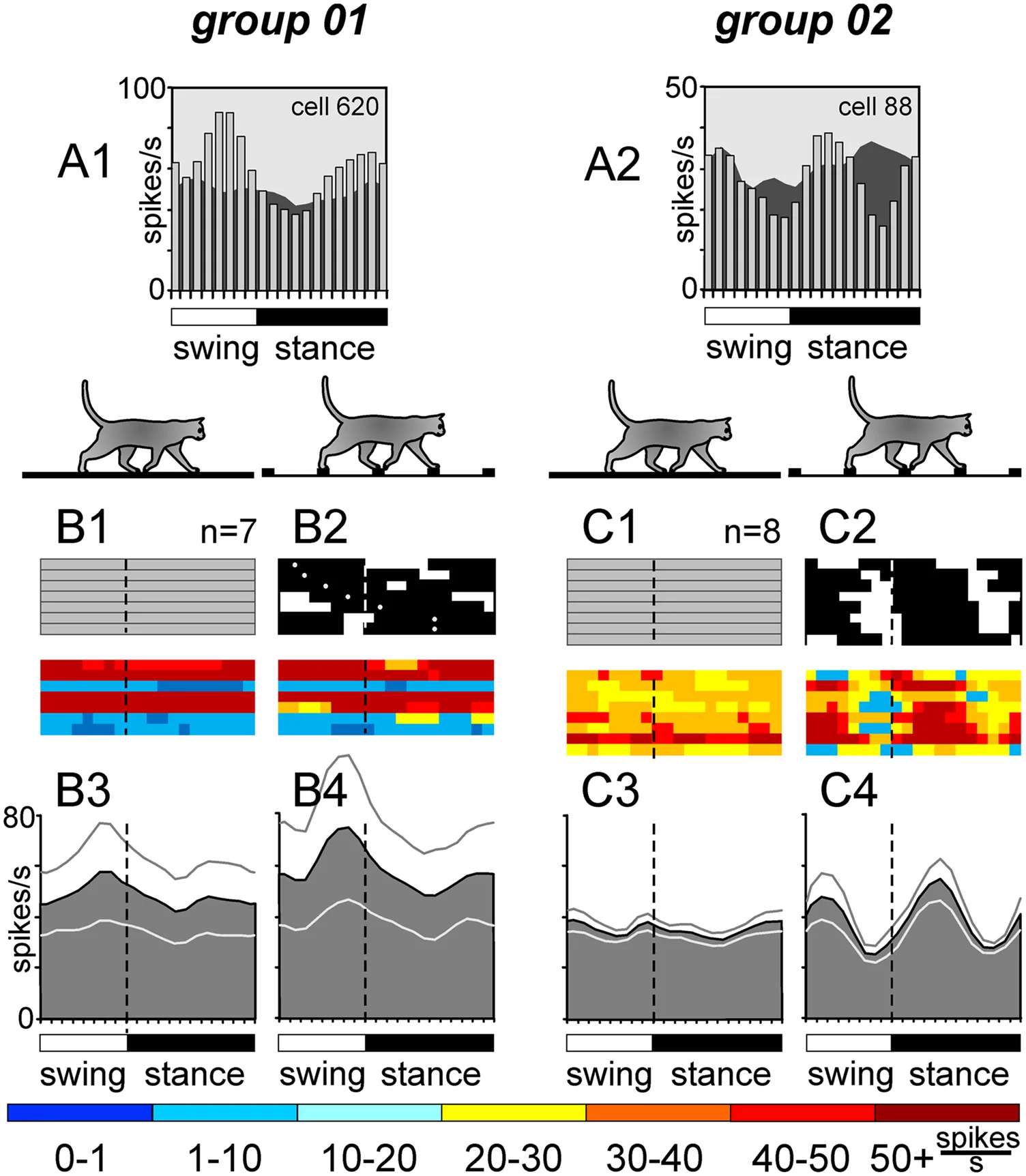
Population characteristics of neurons whose firing on the flat surface was not stride-modulated, but was stride-modulated on the ladder, *group 0–1* and *group 0–2* neurons. **A1**: Example *group 0–1* neuron. On the flat surface, the activity of cell #620 was steady at ~ 50 spikes/s (the dark gray area histogram in the background). On the ladder, the discharge was higher and had one PEF at the end of stance and most of swing (activity on the ladder is shown with a bar histogram). **A2**: Example *group 0–2* neuron. On the flat surface, the firing of cell #88 slightly fluctuated (the dark gray area histogram), not meeting our criteria for being stride-modulated (see Methods). On the ladder, the modulation increased and met the criteria. There were two PEFs: one in the beginning of stance and one at the stance-to-swing transition. The latter PEF emerged because the firing around it diminished; the PEF at the beginning of stance appeared additionally because the firing in this phase increased. **B**: *Group 0–1* neurons (*n* = 7). **B2** (top panel): Phase distribution of PEFs during ladder locomotion. **B1,2** (bottom panels): Corresponding discharge frequencies. **B3,4**: Group mean firing rate during the stride on the flat surface (B3) and ladder (B4). **C**: *Group 0–2* neurons (*n* = 8). Designations are as in Figs. 5 and 7

The activity of 54% (15/28) of neurons with stride-unmodulated firing on the flat surface, was modulated with strides on the ladder. Seven had one PEF and 8 had two PEFs. We called them *group 0–1* and *0–2* neurons, respectively (red in Fig. 8D; Figs. 9A, B). The activities of exemplary neurons are shown in Figs. 10A1,2. The average firing rate of these groups did not change on the flat-to-ladder transition (*p* = 0.693, 0.541, respectively, *t* test), but the rate of half (8/15) of individual neurons altered. On the ladder, PEFs of *group 0–1* cells were distributed across the step cycle, their group firing peaking at end-swing (*p* = 0.012, *U* test for the max/min difference; Figs. 10B2,4). In contrast, PEFs of *group 0–2* cells were synchronized and the group's activity had two prominent maxima: one in early swing and one in the first half of stance (*p* = 0.016, *U* test; Figs. 10C2,4). Across two groups, PEFs were 11% of the cycle longer than PEFs of cells whose firing was also stride-modulated on the flat surface (*p* = 0.006, *t* test). Among 9 tested *group 0–1* and *0–2* cells, 4 did not have a somatosensory RF, suggesting that the emergence of the stride-related modulation on the ladder did not result from stimulation of neurons' RF.

*Groups 0–1* and *0–2* neurons did not transmit any cyclical stride-related information during unobstructed locomotion, and one can suggest they also did not transmit locomotion movement-related information while on the ladder, but only transmitted stride-related vision-based signals about the ladder. In cat 14, these cells resided almost exclusively in the most caudal aspect of the explored area, around P 9.25 (Fig. 2E, panel P 9.25, red symbols).

B Neurons with stride-modulated firing only during flat-surface locomotion, groups 1–0 and 2–0

The firing of 5% (6/129) of neurons, whose activity was stride-modulated on the flat surface, lost modulation on transition to the ladder. On the flat surface, 4 of these cells had one PEF and 2 had two. We called them *group 1–0* and *2–0* neurons (dark red in Fig. 8D; Figs. 9C,D). Out of 5 cells tested, 4 did not have a somatosensory RF, suggesting that the suppression of firing modulation on the flat-to-ladder transition did not result from a change in stimulation of neurons' RF between the tasks, but likely reflected visual processing overriding locomotor movement-related signals. Thus, as for the *group 0–1* and *0–2* neurons, the activity of *group 1–0* and *2–0* cells on the ladder was determined by processing of visual information. 

For most neurons, however, movement-related information contributed to shaping their activity during ladder locomotion. In the following classification, grouping of neurons according to the types of integration of motor and visual information in their discharge was made to correspond to the published classification of neurons in the VL thalamus, one of the main projection targets of the DN [17].

C Neurons active in the same stride phase during both tasks

Among 123 neurons with stride-modulated activity during both locomotion tasks, 37% (45/123) fired in the same phase(s). Eight (7%, all but one with a 1-PEF pattern) retained all other activity characteristics as well. These cells transmitted locomotor movement-related information only, even while on the ladder. The fraction of such neurons in the DN was substantially smaller than in the VL, where it was 24% [17], which suggests that the pure locomotion movement-related information mainly arrives to the VL from sources other than the DN.

**Fig. 11:**
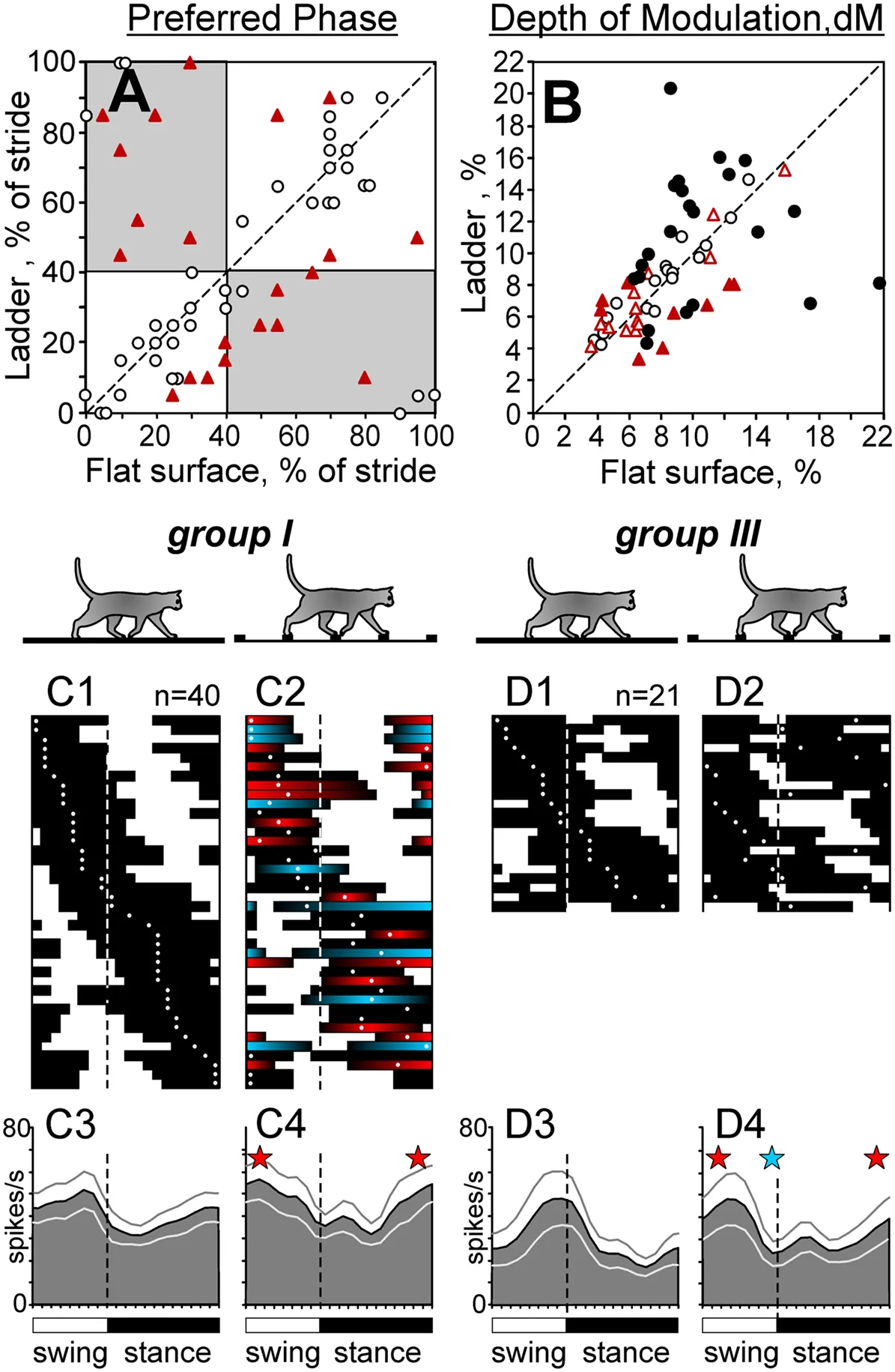
Characteristics of individual neurons and populations of neurons with 1-PEF pattern on both the flat surface and ladder, *group I* and *group III*. **A**: Comparison of the preferred phase of activity, PrPh, of individual neurons during locomotion on the flat surface and ladder. The x- and y-axes of each point show the values of the PrPh for a neuron on the flat surface and ladder, respectively. Neurons whose PrPhs were not statistically different between the tasks are shown by black open circles (*group I* neurons, *n* = 7 + 33). Cells whose PrPhs were statistically different are shown by red triangles (*group III* neurons, *n* = 21). Areas corresponding to the swing phase of one task but stance phase of the other are shaded. **B**: Comparison of the dM during flat-surface and ladder locomotion. Neurons whose dM was statistically different between tasks are shown by filled symbols, others are shown by open symbols. Black filled circles represent *group I* >< *dM* neurons; black open circles show *group I* = *dM* neurons, and triangles represent *group III* neurons. **C**: *Group I* neurons. **C1,2**: Phase distribution of PEFs during flat-surface (C1) and ladder (C2) locomotion. In C1, neurons are rank-ordered so that those active earlier in the cycle are plotted at top of the graph. In C2, neurons are shown in the same sequence as in C1; PEFs of cells, the dM of which was greater on the ladder than flat surface, are marked with a red gradient, PEFs of those whose dM was smaller on the ladder are marked with a blue gradient. **C3,4**: Group mean discharge rate during the stride on the flat surface (C3) and ladder (C4). **D**: *Group III* neurons. **D1,2**: Phase distribution of PEFs during flat surface (D1) and ladder (D2) locomotion. Rank-ordering as in C1 and C2. Note phase shifts of the PEFs between the tasks. **D3,4**: Mean discharge rate during the stride on the flat surface (D3) and ladder (D4). In C4 and D4, red stars indicate stride periods when the firing on the ladder was greater than on the flat surface (*p* <.001, *U* test), the blue star indicates a period where it was smaller (*p* =.002, *U* test). Other designations are as in Fig. 5

For neurons with a 1-PEF pattern during both tasks (Fig. 8D, blue in the 1- > 1 segment), the preferred phase of the activity of 54% (33/61) was similar between the tasks, while other parameters differed. We called them *group I* neurons (Fig. 9I, Fig. 11A, black open circles). They comprised 21% (33/157) of all recorded cells and formed the largest group in respect to the type of the signal sent during ladder locomotion (Fig. 9I). The dM differed for most, being greater in 14/33 (by 37 ± 13%) or smaller in 8/33 (by a similar amount) on the ladder compared to flat surface. This *group I* >< *dM* subgroup, represented 36% (22/61) of 1-PEF neurons (black filled circles in Fig. 11B). The firing rate usually decreased as dM rose (*r* = 0.63, *p* = 0.016). The PEF duration also tended to decrease with higher dM (*r* = 0.74, *p* = 0.023), while the firing rate within the PEF typically increased, by 6–80 spikes/s. The M generally did not change. Thus, upon transition to visually-guided stepping, the salience of the signal from 36% of neurons with one PEF during both tasks changed, more often increasing, and the firing precision tended to rise. The discharge of the entire *group I* on the flat surface had a weak peak in late swing (Fig. 11C3). On the ladder, the group's firing peaked during the stance-to-swing transition (Fig. 11C2,4). This phase shift in the discharge was most prominent in *subgroup I* >< *dM*.

**Fig. 12:**
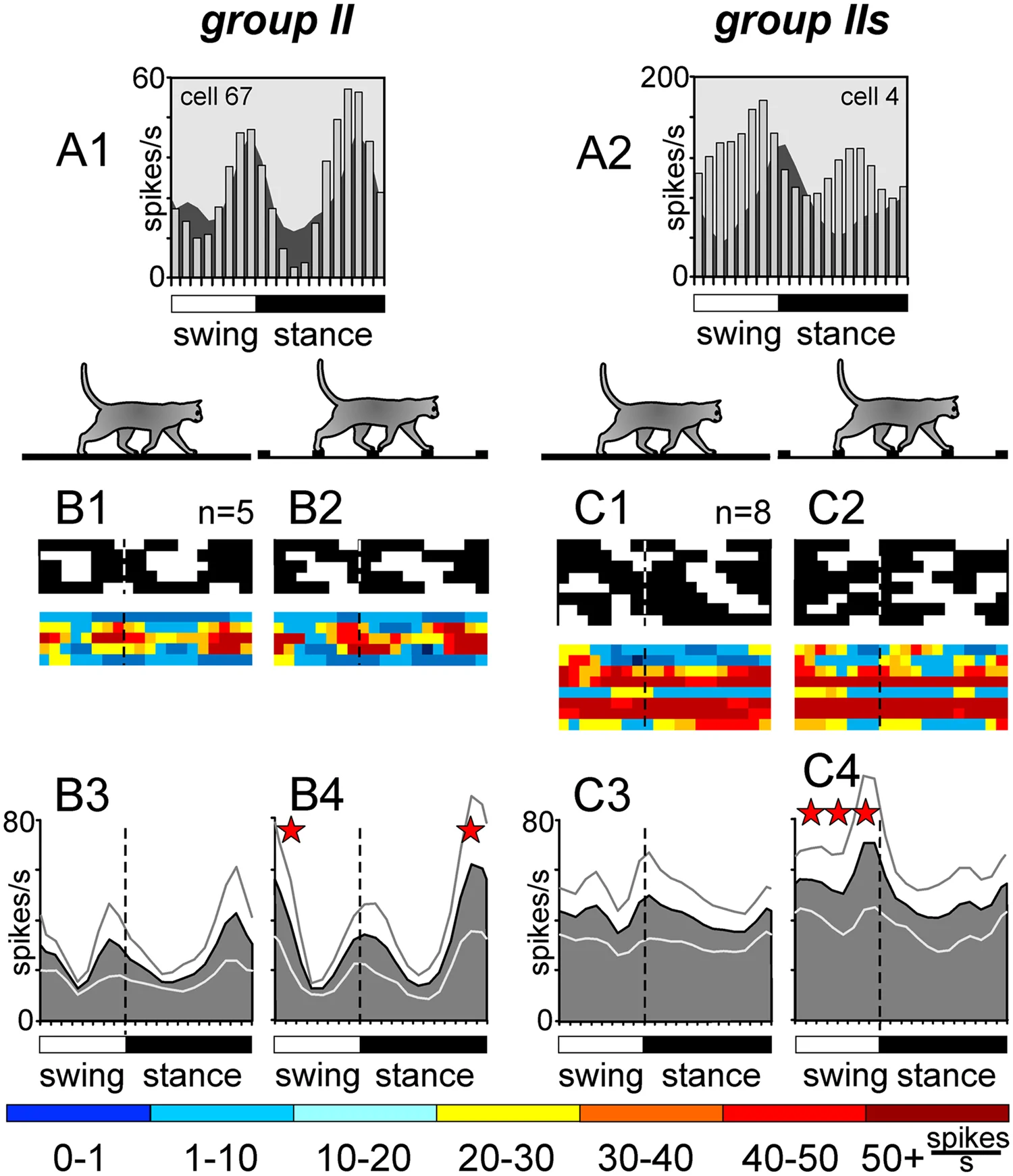
Population characteristics of neurons with two PEFs per stride on both the flat surface and ladder, *groups II* and *IIs*. **A1**: Example *group II* neuron. On the flat surface, the firing of cell #67 had two PEFs: one at end-swing and one at end-stance. On the ladder, these discharge pattern and timing remained, however, the firing rate in the first half of stance diminished while increasing in the second half, which resulted in an increase of the magnitude of the stride-related modulation. **A2**: Example *group IIs* neuron. On the flat surface, the firing of cell #4 also had two PEFs: during swing-to-stance and stance-to-swing transitions. On the ladder, the discharge pattern remained but the timing changed: the PEFs were now in the middle of swing and stance phases. In addition, the firing during one of the inter-PEF intervals increased resulting in a decrease of the modulation. **B**: *Group II* neurons (*n* = 5). **B1,2**: Phase distribution of PEFs during flat-surface (B1) and ladder (B2) locomotion. Top panels show distributions of PEFs; bottom panels show distributions of discharge frequencies. In B1, neurons are rank-ordered so that those active earlier in the cycle are plotted at top of the graph. In B2, cells are rank-ordered as they are shown in B1. **B3,4**: Phase distributions of discharge frequencies for the *group II* population. **C**: Same for *group IIs* neurons (*n* = 8). Other designations are as in Figs. 5 and 10

For neurons with a 2-PEF pattern during both tasks (Fig. 8D, blue in the 2- > 2 segment), phases of the activity of 38% (5/13) were similar between the tasks (Figs. 12A1, B1,2). We called them *group II* neurons (Fig. 9J). Discharges of four of them differed between the tasks in other aspects: the magnitude of modulation (*n* = 2), mean frequency (*n* = 1), or both (*n* = 1). PEFs of *group II* neurons concentrated in swing-to-stance and stance-to-swing transitions (Fig. 12B1,2), producing discharge peaks in these phases during both tasks (Fig. 12B3,4). On the ladder, however, the firing during the stance-to-swing transition was greater than on the flat surface (*p* = 0.036, *U* test), while the swing-to-stance discharge peak was unchanged.

Thus, the firing pattern and phase of 37% (45/123) of stride-modulated neurons with the same 1-PEF (*n* = 7 + 33) or 2-PEF (*n* = 5) pattern during both tasks were determined by locomotion movement-related information. Vision-related input adjusted the magnitude of the modulation (salience) in about half, and for some neurons also adjusted the average and/or intra-PEF rates and the PEF duration (precision). In cat 14, these neurons were located in the middle of the explored area, P 8.75—P 9.0 (Fig. 2E, P 8.75—P 9.0, blue symbols).

D Neurons active in different stride phases during the two tasks

Of 123 neurons with stride-modulated firing during both locomotion tasks, 63% (78/123) fired in different stride phases between the tasks. Among 1-PEF cells, the preferred phase of one third (34%, 21/61) differed (Fig. 8D, orange in the 1- > 1 segment; Fig. 11A, red triangles). We called them *group III* neurons (Fig. 9E). They accounted for 13% (21/157) of the entire sample and were the distant second largest group in respect to the type of the signal sent during ladder locomotion (Fig. 9E). The average PrPh difference was 28 ± 17% of the cycle (mean ± SD; Fig. 11A). The direction of the PrPh shift appeared to be related to the somatosensory RF of the neuron. Most (7/10) cells with a PrPh earlier in the cycle while on the ladder had a hindlimb-related field, whereas 7/8 cells with a PrPh later in the cycle did not have a RF. These phase shifts in firing of individual *group III* neurons shifted the group activity peak from late swing on the flat surface to the first half of swing on the ladder (Fig. 11D). This refocusing must have heavily contributed to the shift of the peak firing of the entire 1-PEF subpopulation to earlier in the cycle upon the flat-to-ladder transition (Fig. 5C4 *vs.* 5A4). Besides the phase shift, *group III* neurons could also change the strength of the stride-related modulation. The dM altered in 43% (9/21) of these neurons, decreasing in 6 or increasing in 3 cells by 44 ± 12% (mean ± SD; red filled triangles in Fig. 11B*)*. As in *group I* neurons, the average discharge rate often decreased with the dM increase (*r* = 0.71, *p* = 0.032). The M coefficient changed by 20–50% in 19% (4/21) of the neurons. The PEF duration usually did not change.

Among 2-PEF neurons, the activity phases of two-thirds (62%, 8/13) were also different between the tasks, with the peak of one (3 cells) or both (5 cells) PEFs being ≥ 20% of the cycle away from a peak seen on the flat surface (Figs. 12A2, 12C1,2). We called them *group IIs* neurons ("*s"* for shifters; orange in the 2- > 2 segment of Fig. 8D, Fig. 9F). In 3 cells, the shift of the PEF(s) was accompanied by a dM change, which decreased in two cells (e.g., cell #4 shown in Fig. 12A2) or increased in one. Shifts of PEFs of individual neurons contributed to increased group activity during swing (*p* < 0.001, *U* test; Fig. 12C4).

Thus, for *groups III and IIs* neurons, the locomotion movement-related information set the 1-PEF or 2-PEF pattern during both tasks. On the ladder, vision-related input dominated by: shifting the phase(s) of the PEF(s) and adjusting the magnitude of the stride-related modulation in ~ 40% of the cells. In cat 14, these neurons were found throughout the explored DN area (Fig. 2E, yellow and light orange symbols).

**Fig. 13:**
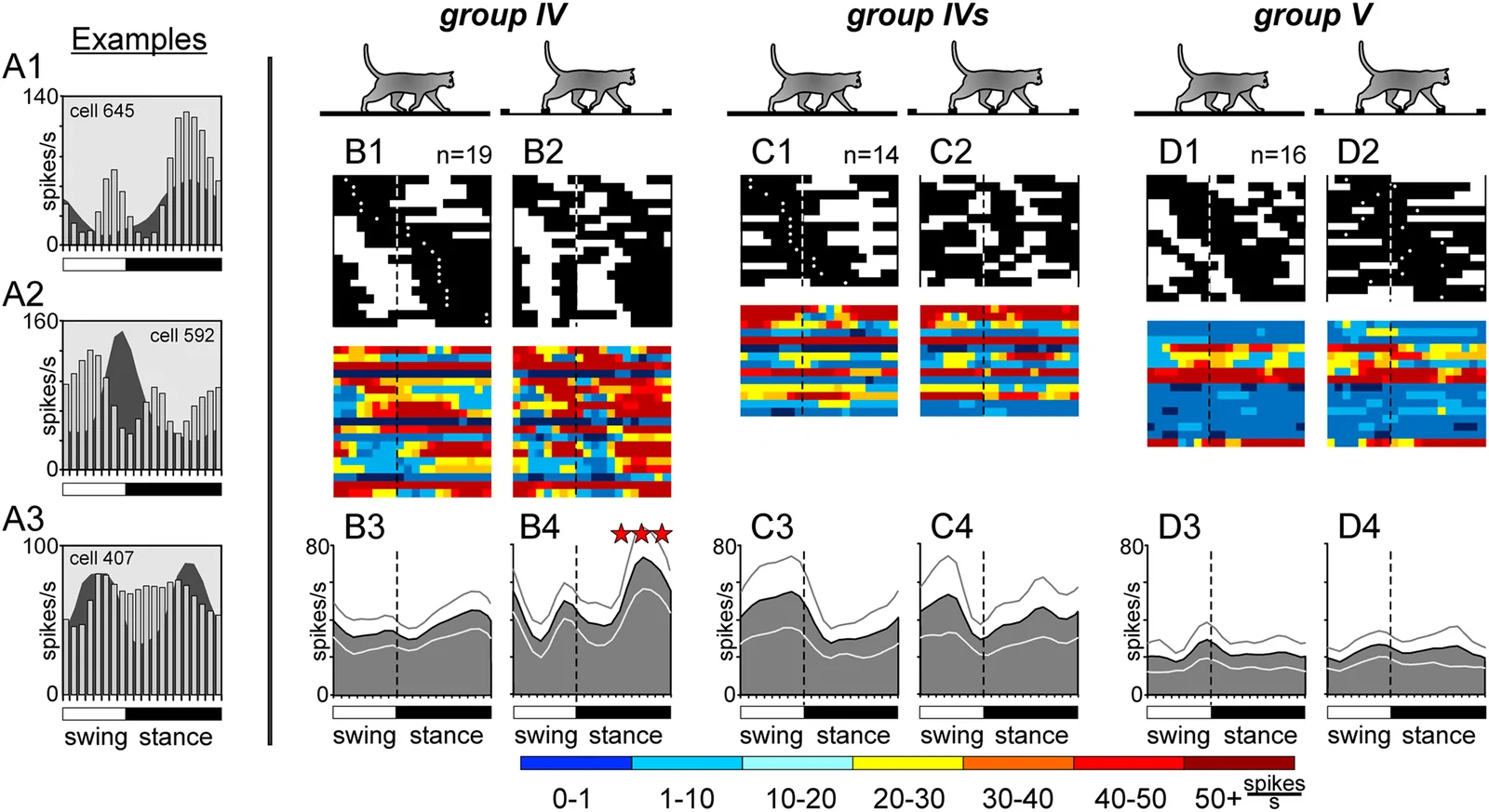
Population characteristics of neurons with different number of PEFs per stride on the flat surface and ladder, *groups IV*, *IVs, V* and *Vs*. **A1**: Example *group IV* neuron fires an additional PEF upon the flat-to-ladder transition. On the flat surface, cell #645 had one PEF with a peak in late stance. On the ladder, it still had a PEF in late stance and also fired a PEF at end-swing. **A2**: Example *group IVs* neuron shifts firing to different stride phases, resulting in a complete overhaul of the firing pattern upon the flat-to-ladder transition. On the flat surface, cell #592 had one PEF in late swing. On the ladder, there were two PEF's: one in beginning of swing and one in beginning of stance. **A3**: Example *group V* neuron fires one instead of two PEFs upon the flat-to-ladder transition. On the flat surface, cell #407 had two PEFs: one in mid-swing and one in mid-stance. On the ladder, the cell's firing in the beginning of stance increased, merging the two PEFs into one. **B**: *Group IV* neurons (n = 19). **B1,2**: Phase distribution of PEFs during the flat-surface (B1) and ladder (B2) locomotion. Top panels show phase distributions of PEFs; bottom panels show corresponding distributions of discharge frequencies. In B1, neurons are rank-ordered so that those active earlier in the cycle are plotted at top of the graph. In B2, neurons are rank-ordered in same sequence as they are shown in B1. **B3,4**: Phase distributions of discharge frequencies of *group IV* population. **C**: Same for *group IVs* neurons (*n* = 14). **D**: *Same for group V* neurons (*groups V* [n = 7] and *Vs* [n = 9] are combined). Designations are as in Fig. 5

Besides a phase shift, the number of PEFs often changed between tasks. A third (34%, 33/98) of cells with one PEF on the flat surface discharged two PEFs on the ladder (light orange in Fig. 8D). We called them *group IV* (n = 19) or *group IVs* (*n* = 14) neurons, depending on whether the phase of the flat-surface PEF shifted (Figs. 9G,K). Figures 13A1,2 show typical examples. In 68% (13/19) of *group IV* cells, a second PEF emerged in the former trough (e.g., Fig. 13A1). In 32% (6/19), a long PEF seen on the flat-surface split into two on the ladder (not illustrated). Along with these changes, dM increased in 74% (14/19) of these cells, and M increased in 6 cells as well. In addition, the average total duration of the two PEFs was 11% shorter than the mean duration of their single PEFs on the flat surface *(p* = 0.010, *t* test), making this group's firing more stride phase-precise on the ladder. *Group IV* neurons represented 12% (19/157) of the sample and were the third most populous group with respect to the type of the signal sent during ladder locomotion. On the flat surface, the firing of *group IV* peaked at the end of stance (Fig. 13B1,3). Upon transition to the ladder, the fraction of cells with a PEF at end-swing increased (*p* < 0.001, χ^*2*^ test; Fig. 13B2), leading to formation of a second, albeit small, group activity peak in this phase (Fig. 13B4). In addition, the peak at end-stance increased. Among the 14 *group IVs* neurons, whose PEF phase changed, the firing of 9 was completely overridden by an emergence of two new PEFs in completely new phases (e.g., Fig. 13A2). In the other 5 cells, the PEF shifted, and a new trough formed. Unlike in *group IV*, dM of *group IVs* cells often decreased on transition to the ladder, and their group activity profile did not significantly change (Figs.13C3,4).

Half (53%, 16/30) of neurons with two PEFs on the flat surface had only one PEF on the ladder (dark orange in Fig. 8D). We called them *group V* (*n* = 7) or *group Vs* (*n* = 9) cells depending on whether the phase of the flat-surface PEF changed (Figs. 9H,L). In most (*n* = 11), one PEF simply disappeared. In 5 cells, the transition occurred due to increased firing between the two PEFs, merging them (e.g., Fig. 13A3).

Thus, *groups IVs* and *Vs* neurons together with *IIs and III* cells comprised 42% ((14 + 9 + 8 + 21)/123) of DN neurons stride-modulated during both tasks, for which visual input was the main modulator of their firing during ladder locomotion. For *group IV* and *V* (21% of this population, (19 + 7)/123, contributions of movement- and vision-related information were at par. The locomotion movement-related information set the phase of one of the PEFs during both tasks, while the vision-related information set the phase of the other PEF (*group IV*) or suppressed one (*group V*). Vision-related information also enhanced the salience of *group IV* signal by increasing the magnitude of stride-modulation in most cells while shortening their PEF. These activity adjustments might have aided in precise placement of feet on the ladder. In cat 14, these neurons were found throughout the explored DN area (Fig. 2E, yellow and orange symbols).

E Neurons with firing not stride-modulated in either task

The firing of 13 cells (8% of the sample) was not stride-modulated during either locomotion task. Nine were from cat JS. Across all cats, their average firing rate during standing was 50.2 ± 9.5 spikes/s. This rate remained unchanged upon transition to walking on the flat surface or ladder. Three of these cells had a RF on the head or neck, three were activated by a movement of either forelimb, and three were unresponsive. On the flat-to-ladder transition, the firing rate of only two of these cells changed, decreasing by 10–15%. Thus, the activity of the great majority of these cells did not encode either rhythmic or static locomotor aspects.

The data on the integration of motor and visual information in the activity of individual DN neurons can be summarized as follows. Across 144 neurons with stride-modulated firing during at least one of the locomotion tasks, 15 gained and 6 lost modulation on the flat-to-ladder transition, and the depth of modulation of 64 neurons with stride-modulated firing during both tasks changed (Fig. 8A). Thus, the salience of the signal changed in 59% (85/144) of neurons in this population. The discharge rate (magnitude of the signal) changed in 54% (78/144; Fig. 4G). The phase of the firing of 63% (78/123) neurons with a stride-modulated activity during both tasks changed (*groups IIs*, *III*, *IV*, and *V*), and the duration of PEF(s) (precision of firing) changed in 30% (37/123; Fig. 8B). These changes could be classified into 3 broad categories, comprised of 12 groups, in respect to the manner of integration of motor and visual information and resulting signal sent during vision-guided stepping (Fig. 9). These groups are analogous to those reported earlier for neurons of the VL thalamus (Figs. 10, 11 and 12 in [17]). The described activity changes represent individual DN neurons' signals that combine motor and visual information to guide accurate stepping on complex surface.

### Relationship of the Vision-related Activity Components of DN Neuronal Groups with Gaze Behaviors

**Fig. 14:**
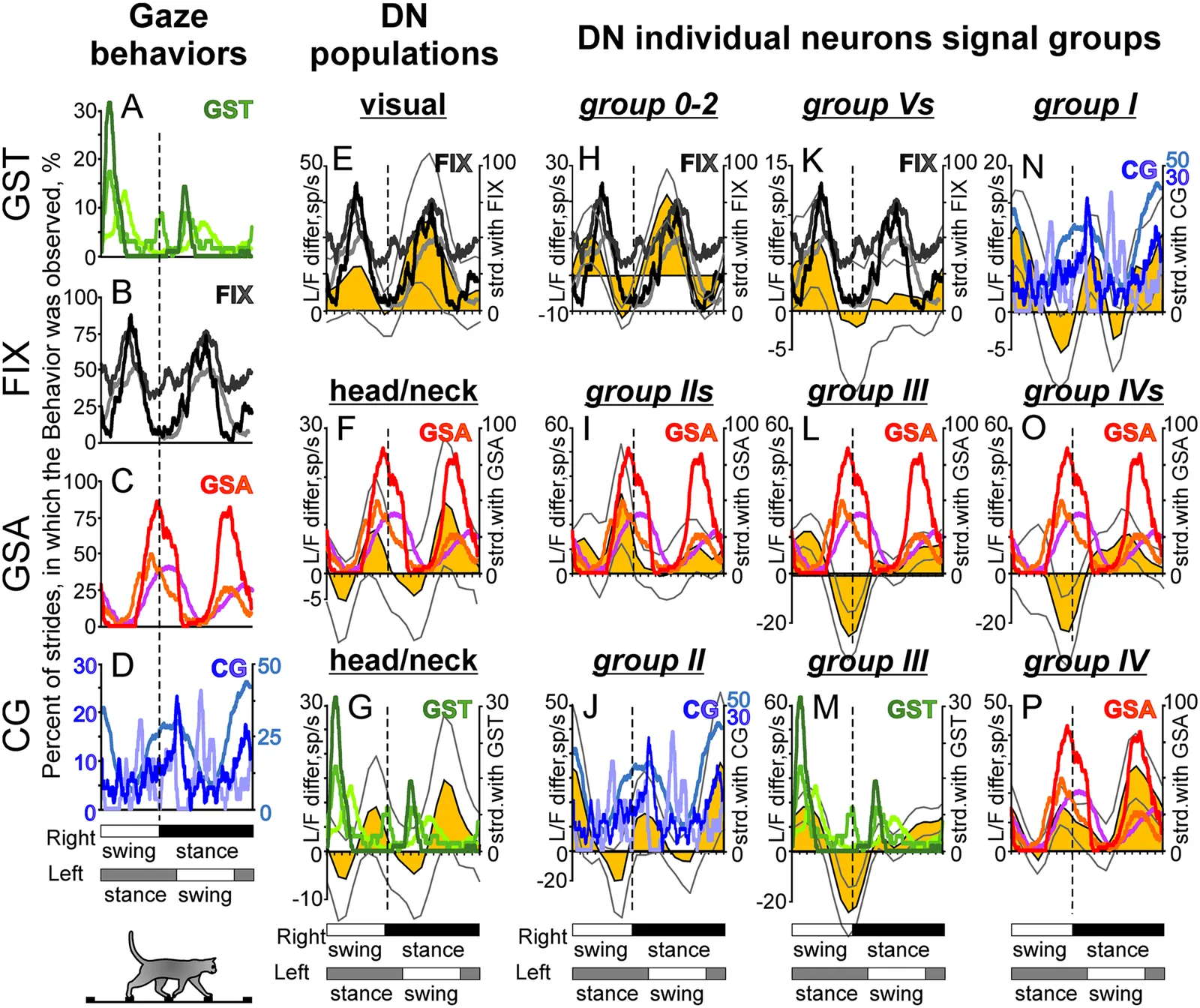
Relationship of the vision-related components of the activity of DN neuronal groups with four gaze behaviors. **A–D**: Frequency distributions of gaze behaviors in the step cycle of ladder locomotion obtained from three cats in a previous study (modified from Fig. 14 in [51]). Three different shades of the same color show data from three different cats. **A**: Gaze shifts along the ladder toward the animal, GST. **B**: Gaze fixations on the ladder, FIX. **C:** Gaze shifts along the ladder away from the animal, GSA. **D**: Constant gaze along the ladder, CG. **E- G:** Relationship of the vision processing-related activity component of DN neuronal groups with a visual (E) and head & neck-related RF (F, G) with the frequency distribution of gaze behaviors. In each panel, the yellow area histogram is the bin-by-bin difference between the average activity of the group of neurons during the flat-surface and ladder locomotion (grey lines show SEM). Overlaid line graphs show the frequency distribution of a gaze behavior of the three cats that best match the profile of the component. Left y-axis: the activity component (spikes/s). Right y-axis: frequency of the gaze behavior (percent of strides [strd.] during which it was observed). **E**: The vision-related activity component of neurons with a vision-related RF, plotted with the frequency distribution of gaze fixations, FIX. The activity component was calculated as the difference between the group's average firing rates during flat-surface and ladder locomotion plotted in Figs. 7B4 & 7E4. **F**, **G**: The vision-related activity component of neurons with a head & neck-related RF (calculated as the difference between average firing rates of the group during the two tasks as shown in Figs. 7A4 & 7D4), plotted with the frequency distribution of gaze shifts along the walking surface away from the cat, GSA (F), and toward the cat, GST (G). **H-P:** Analogous plots for neuronal groups defined in Fig. 9. The activity component for a group was calculated as the difference between the average firing rates of the group during the flat-surface and ladder locomotion as shown for: **H**, in Figs. 10C3 & 10C4;** I**, in Figs. 12C3 & 12C4; **J**, in Figs. 12B3 & 12B4; **L & M**, in Figs. 11D3 & 11D4; **N**, in Figs. 11C3 & 11C4; **O**, in Figs. 13C3 & 13C4; **P**, in Figs. 13B3 & 13B4. See text for details

Vision-related activity components of some DN neuronal groups coincided with specific gaze behaviors. In a study of cat gaze during flat-surface and ladder locomotion, we found that four gaze behaviors repeat twice per stride [51] Fig. 14 A-D). At the onset of a forelimb swing, gaze shifts along the walkway toward the cat, i.e., the cat starts looking at a closer site (gaze shift toward, GST; Fig. 14A). Mid-swing is dominated by gaze fixations (FIX, Fig. 14B). In late swing, gaze shifts away (GSA; Fig. 14C). Finally, at stance onset, during the forelimbs’ double-support phase (illustrated e.g. in Fig. 15 in [51]) constant gaze (CG) dominates (Fig. 14D). This sequence repeats with the other forelimb’s movement. Although FIXs and GSAs occur slightly earlier in the cycle on the ladder than flat surface, the overall pattern remains the same. This means that the difference in the activity of neurons between two locomotion tasks chiefly reflects the difference in processing of visual information, not in oculomotor behavior or head movement (for the pattern of cat head movement during locomotion see [52]).

Mirroring the sequence of gaze behaviors during the stride, different DN neuronal groups activated in sequence. To visualize the relation of neuronal group responses to gaze behaviors, Figs. 14E-P plot the differences in their activity between the flat-surface and ladder locomotion as yellow area histograms. These histograms are overlaid on the line graphs of gaze behavior distributions, replotted from Figs. 14A-D. For each neuronal group, the gaze behavior best matching the profile of its activity component related to visuomotor demands of ladder locomotion is shown.

For the group of visually responsive cells, the profile of this component resembled FIX (Fig. 14E), both peaking in mid-swing. This temporal correspondence is consistent with the suggestion that these neurons transmit visual information obtained during gaze fixations. For cells with a head & neck RF, the component coincided with or slightly preceded GSA peaks (Fig. 14F), and also had a trough in early swing that coincided with GSTs (Fig. 14G). This suggests the activity component of head & neck-receptive neurons that is related to visuomotor demands of the ladder task reflects gaze shifts while encoding their direction.

For the *group 0–2* neurons, those whose firing was only stride-modulated when visual control was needed on the ladder, the profile of this component resembled FIX, with a stronger, tighter signal during the contralateral (left) forelimb's swing (Fig. 14H). The vision-related component of *group Vs* activity also peaked simultaneously with FIX (Fig. 14K). This suggests these neuronal group process visual information obtained during fixations. The profile of the vision-related activity component of *group 0–1* neurons did not follow any gaze behavior (not illustrated).

The activity component of *groups IIs and III* related to visuomotor demands of ladder locomotion peaked with gaze shifts: *group IIs* with GSAs at swing end (Fig. 14I), *group III* with GSTs in early swing (Fig. 14M). The *group III* signal also had a negative peak at the end of swing, during GSAs (Fig. 14L). A similar negative element was also seen in *group IVs* activity (Fig. 14O). The vision-related component of *group IV* activity coincided with GSAs at the end of swing of each forelimb (Fig. 14P). Visual sampling is suppressed during fast gaze shifts ([53], rev. in [54]), and these neuronal groups may signal this.

Finally, the vision-related activity components of *groups I* and *II*, peaked during forelimbs' double-support phase, coinciding with the peak of constant gaze (CG, Figs. 14J,N), suggesting these neurons process visual information obtained during constant gaze.

## Discussion

There are four main findings in this study. First, 82% of DN neurons have stride-related modulation of the firing during locomotion on the flat surface, a task not requiring visual control and step accuracy. Since the activity of only 14% of cells was modulated in the decerebrate preparation [55], the wide-spread modulation of the firing in the awake cat indicates that most modulation originates from the forebrain. Second, the firing of 88% of neurons change when cats switch from vision-independent locomotion to vision-guided accurate stepping on the ladder. The salience of the signal changes in 59% of neurons involved in cyclic stride-related activity during at least one of the locomotion tasks, and the magnitude of the signal changes in 54%. The phase of the firing changes in 63% of cells with a stride-related firing during both tasks, and the precision of firing changes in 30%. These changes, representing signals from individual DN neurons, presumably contribute to visual guidance of feet to desired locations. Third, we compared several DN neuronal subpopulations: cells with inputs from different segments of the limb or the head & neck, cells responding to visual stimuli, not responding to any external stimuli, and those with 1-PEF and 2-PEF discharge patterns. Each group displayed distinct activity during unobstructed locomotion and also sent different signals during visually guided stepping on complex surface. Four, some of these signals coincided with specific gaze behaviors, which suggests transmission of distinct visual information.

### DN Signals During Vision-independent Locomotion

#### Origin of Stride-related Activity in DN During Locomotion on a Flat Surface

During flat-surface locomotion, the firing of 82% of DN neurons was stride-modulated, consistent with our preliminary reports in awake cats [56, 57], but sharply contrasting with the data of [55], who found modulation in only 14% of neurons in decerebrate cats (33/243; *p* < 0.001, χ^*2*^ test). Both studies explored the rostral DN, so recording location cannot explain the difference. It has to be concluded that, in the awake cat walking on the flat surface, forebrain signals drive most stride-related DN modulation. Primary motor, premotor, somatosensory, and parietal area 5 cortices are all good candidates, as the activity of most neurons there is stride-modulated [21, 32, 58–66]. Neurons in these areas project to the lateral cerebellum via the pons, though direct ponto-dentate projection is sparse compared to the one to the cerebellar cortex [67, 68]^69^; [16, 70–76]. High firing rates of DN neurons during locomotion, despite inhibitory input from the cerebellar cortex, may be due to, among other factors, the locomotion-related input from the basal ganglia. This input comes via projections from the globus pallidus pars interna to fields of Farrell and pedunculopontine nucleus, from where the signal goes to the nucleus reticularis tegmenti pontis to be relayed to the lateral cerebellum, including the DN [75, 77–79]. There is also a projection from the subthalamic nucleus to the pedunculopontine nucleus, etc. [80, 81]. Indeed, the motor, premotor, and posterior parietal cortices all project to the striatum and subthalamic nucleus [82–87], and neuronal activity in various parts of the basal ganglia is modulated in the rhythm of strides on the flat surface [88–92], including the stride-modulated firing of 49% of neurons in the entopeduncular nucleus, the feline equivalent of the globus pallidus pars interna [88], and 9% in the subthalamic nucleus [91]. Further studies will determine the respective roles of different cortical areas in modulating the activity of DN neurons during vision-independent locomotion, and the specific routes by which they exert their influence. Greater discharge variability of DN neurons, CV, compared to that of pyramidal tract neurons in motor cortex (2.80 ± 0.17 *vs.* 1.85 ± 0.12 [41]], *p* < 0.001, *t* test) is likely due to an added variability at subcortical synapse relays.

We have previously suggested that the stride-modulation of the firing of neurons in the primary motor cortex, primary somatosensory cortex, premotor cortex area 6, and posterior parietal cortex area 5, as well as in the VL thalamus during locomotion on the flat surface to a great extent is determined by efference copy signals from the spinal cord locomotor central pattern generator, CPG [21, 59, 61, 65, 93]. The locomotor CPG is a network of interneurons in the spinal cord that generates the rhythmic pattern of locomotion [94–98], rev. in e.g., [99]. If this suggestion is correct, one should conclude that signals DN receives from those areas during flat-surface locomotion chiefly carry information about the operation of the spinal locomotor CPG. This is an extension of the hypothesis of Arshavsky and colleagues [100] that all commands transmitted from the brain to the spinal cord during locomotion take into account the current state of the spinal mechanisms, the phase of the locomotor cycle. This is accomplished by modulating the activity of all the brain centers involved in control of locomotion in the rhythm of strides. Such modulation allows these brain centers to adjust stepping without disturbing the basic locomotor pattern.

The fact that the activity of 14% of DN neurons in decerebrate cats was stride-modulated [55] shows that some information about the rhythmic locomotor process arrives to the nucleus from the spinal cord and brainstem. Signals from the spinal cord ascend to the cerebellum via various spinocerebellar and spinobulbar tracts (e.g., [100–102]. Though neurons of these tracts do not synapse on DN cells, information they carry can affect DN neuron activity via indirect spinocerebellar pathways [103].

#### Contribution of Peripheral and Internal Information to DN Neuronal Activity During Locomotion on the Flat Surface

The firing of somatosensory-responsive DN neurons increased from standing to locomotion, and during locomotion these neurons were more active than the non-responsive ones (Fig. 4C, middle). Cells responsive to limb movement or muscle palpation were more active than the head & neck-responsive ones. Stride-related modulation was more common and stronger among somatosensory-responsive cells than non-responsive ones. These observations suggest that somatosensory information, particularly from the limbs, plays an important role in activating and modulating discharges of DN neurons during locomotion. Yet, the firing of 74% of neurons without a somatosensory RF was stride-modulated as well (Fig. 7C). Moreover, in decerebrate cats, it was found that DN neurons with stride-modulated firing do not respond to movement perturbations of a single limb, and their discharge can be easily uncoupled from muscle activity by perturbing the step cycle [55]. This indicates that internal locomotion-related processes, rather than somatosensory information, drive the activity of DN neurons during flat-surface locomotion. This drive, however, appears to be stronger for cells with a somatosensory RF, especially those with a limb-related field. Such a preference may be useful if one assumes that in the DN, as in the primary motor cortex [104–107] there is a correspondence between the location of the source of somatosensory input and the target of motor output. If there is one, DN cells with a limb-related output would receive a stronger drive from the locomotor CPG and be in a better position to integrate visual information about the terrain into their locomotion-related activity to adjust the locomotor movement.

### DN Influence on the VL During Locomotion on the Flat Surface

**Fig. 15:**
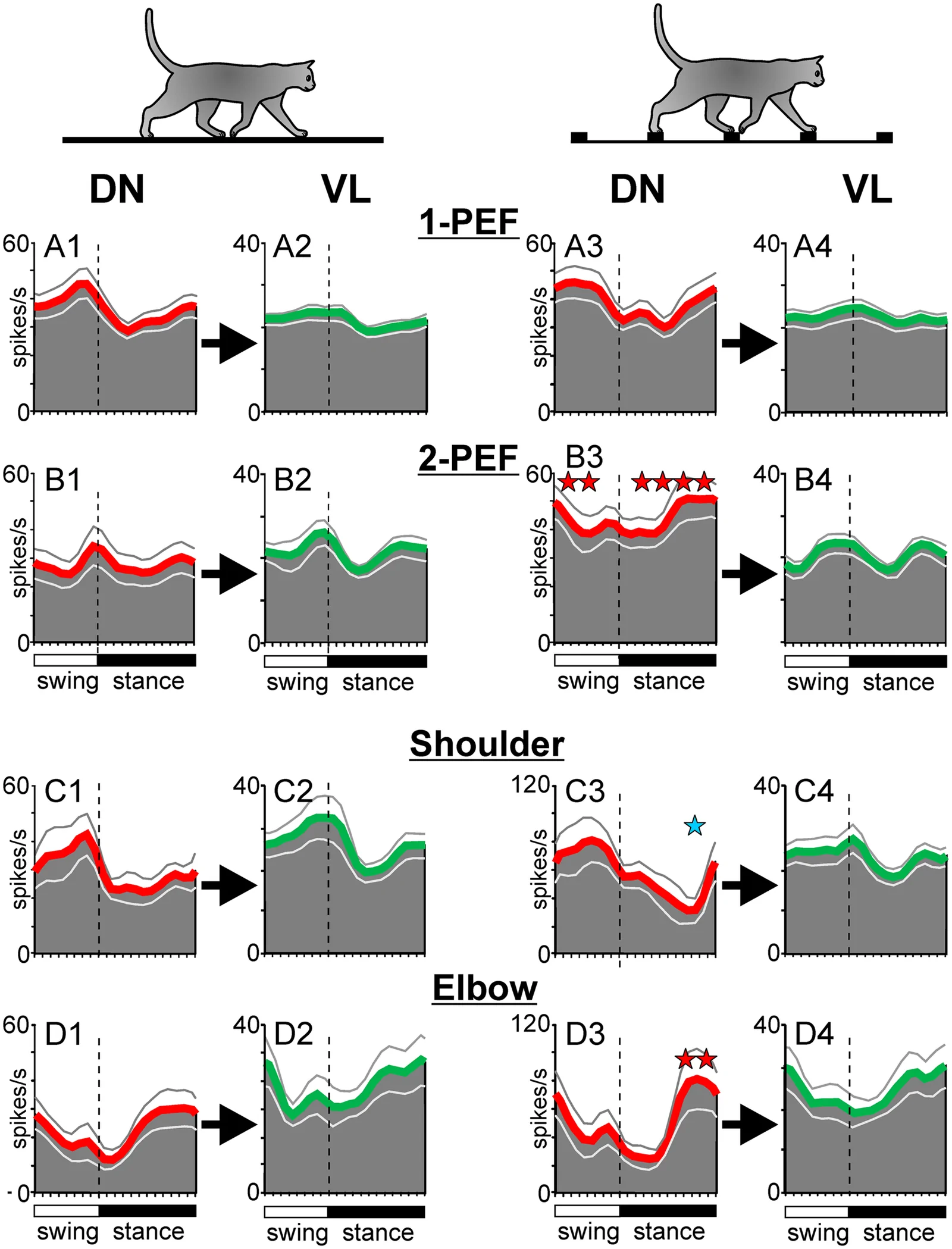
Comparison of activity profiles of 1-PEF, 2-PEF, shoulder- and elbow-related populations in the DN and VL thalamus. **A1—D1**: Group activity profiles during locomotion on the flat surface of 1-PEF (A), 2-PEF (B), shoulder-related (C), and elbow-related (D) neurons of the DN. **A2—D2**: Same for the VL. **A3—D3**: Group activity profiles during locomotion on the ladder of 1-PEF (A), 2-PEF (B), shoulder-related (C), and elbow-related (D) neurons of the DN. **A4—D4**: Same for the VL. Graphs for DN groups are replotted from Figs. 5A4-D4 and Figs. 6A4,B4,D4,E4. Graphs for VL 1-PEF and 2-PEF groups are adopted from Figs. 6E,K and 8D,I in [17]. Graphs for VL shoulder- and elbow-related groups are adopted from Figs. 8D4,E4 and 10D4,E4 in [18, 20]

On the flat surface, 63% of DN neurons fired one burst per stride, similarly to VL cells (62% [148/238]; [17]), while 19% fired two bursts, fewer than in the VL (29% [69/238]; *p* = 0.027, χ^*2*^ test). The firing of 1-PEF DN population peaked at the end of swing (Fig. 5A4 & Fig. 15A1) and did not translate into a similar activity pattern of VL 1-PEF population, which was not stride-modulated (Fig. 15A2; [17]). In contrast, the firing of the 2-PEF DN group that had peaks at the swing-to-stance and stance-to-swing transitions appeared to be reflected in the two-peaked activity profile of the VL 2-PEF population (Fig. 5B4 & Fig. 15B1, B2). Firing profiles of the DN shoulder & elbow and elbow only groups, while being distinct from each other (Figs. 6A,B *&* Figs. 15C1,D1), each had a profile similar to that of the VL shoulder- and elbow-related groups (Figs. 15C2,D2; [18, 20]. This suggests that during flat-surface locomotion DN neurons associated with the shoulder and elbow directly activate the corresponding VL cells.

### DN Signals During Vision-guided Accurate Stepping

#### Content of Signals

In this study, we characterized components of the DN neuronal activity related to visual guidance of feet to desired locations during locomotion on complex surface of a horizontally placed ladder. We have previously shown that the mechanics of locomotion on the flat surface and the "convenient" ladder with crosspieces just wide enough to fully support the paw and separated by half an average step length, are rather similar [32]. In the forelimb, only slight differences in the wrist movement and activity of some wrist-related muscles were seen between flat-surface and convenient ladder locomotion. In addition, cats had a more bent-forward posture while walking on the ladder. In our previous publications, we have considered potential sources, besides the use of vision, that might cause differences in neuronal activity between locomotion on the flat surface and such a ladder (e.g., [17]). In Beloozerova et al [32], we discussed whether the difference in posture, particularly the downward rotation of the head & neck during ladder locomotion compared to flat-surface, modulates limb-muscle activity during locomotion, and concluded that this activity more likely reflects the difference in the muscle control mechanisms between vision non-requiring and vision-guided locomotion. Minor variations in proprioceptive afferentation or the difference in attention (e.g., [108]) might have a role, however, they cannot account for the full volume and scope of the observed differences in neuronal discharges between two tasks. The major demand of walking along the ladder is the necessity to use vision and step on specific sites. Despite much practice, cats need to see the ladder during every step to land feet on the crosspieces [33, 50, 109]. Cats move their eyes and look at the pathway in different manners during flat-surface and ladder locomotion [32, 110]. Their gaze behavior is synchronized with strides, being part of the whole-body locomotion synergy; and noteworthy, on the ladder this synchronization increases [51]. See also [111] for humans. Taking together these considerations, we maintain that the differences in the firing behavior of neurons during the flat-surface and convenient ladder locomotion are caused primarily by processing visual information and formation vision information-based motor commands for accurate target stepping.

Visual cortical areas 17–19 are the source of projections to the rostral half of the pontine nuclei [112–117]. These nuclei also receive visual input from the superior colliculus and pretectum (e.g., [118, 119]) as well as the ventral lateral geniculate nucleus [120]. Consequently, cells in the pontine nuclei readily respond to visual stimuli, particularly moving ones, showing directional specificity [119, 121–124]. They project widely to the caudal cerebellum, most heavily the paraflocculus (e.g., [125–127], rev. in [14, 16], which in turn projects to the DN and interposed nuclei. Visual input to the cerebellum also comes from the inferior olive (e.g., [128, 129]).

During locomotion on complex terrain, visual information about stepping targets is first processed through visual networks and then incorporated into the locomotion rhythm to guide limbs. From this point on, it becomes integrated “visuo-motor” information that, in the afferent sense, is the “[processed] visual information,” while in the efferent sense it is the “limb control signal” reflecting preparation of the desired movement, an irreducibly sensorimotor signal. While in this study we did not investigate neuronal correlates of movement accuracy exhaustively, such as by testing responses of neurons in a dartboard-like task (e.g., [32]), we did investigate responses of the neurons to introduction of a requirement for the movement to be accurate. These responses were, for the most part, the results of processing of visual information about the walking surface and, simultaneously, the control signals for accurate feet placement on the ladder.

### Responses of DN Neuronal Populations to the Ladder Task, and Potential Contribution of DN Subpopulations to Processing of Visual Information for Accurate Stepping on the Ladder

DN neuronal subpopulations responded differently to the ladder task. The average discharge of 1-PEF subpopulation peaked earlier in the swing phase on the ladder than flat surface (Figs. 5A4, C4). This suggests more proactive control of limb transfer on the ladder. Experiments with a displaceable crosspiece showed that cats still can adjust foot transfer and accurately step on the crosspiece as long as the displacement completes within the first half of swing (Beloozerova, unpublished observation). Thus, the earlier firing of 1-PEF DN neurons during swing could assist feet placement on the ladder.

The fraction of 2-PEF neurons rose from 19 to 34% upon the flat-to-ladder transition; their firing rate increased, particularly during stance-to-swing transition (Figs. 5B4,D4), and the average modulation depth reached that of the 1-PEF group. The fractions of 1-PEF and 2-PEF neurons we observed were similar to those reported by [28]. During ladder locomotion, 2-PEF neurons are relatively rare in primary motor, premotor, and high subdivisions of primary somatosensory cortices (14–27%; [41, 59, 61]), but are common (38%) in posterior parietal cortex area 5 [50, 59]. As noted, area 5 is one of the cortical areas projecting to the lateral cerebellum via the pons. During locomotion on a complex surface, its neuronal activity reflects the visual heterogeneity of the walking surface [50] and the distance and time to contact with an obstacle [130]. Recently, we found that 2-PEF neurons in layers II–IV of area 5 are active during the phase of the stride when constant gaze behavior dominates [59]. Constant gaze, or "travel fixation", occurs when a subject looks a fixed distance ahead during locomotion [131–133], and images of objects flow across the retina in a constant pattern. Such "optic flow" provides information about both environment and self-motion [134–137]. We found, however, that this information is not transmitted to the motor cortex by a coherent activity of cortico-cortically projecting cells of area 5 [59]. The increased fraction of 2-PEF neurons in the DN on the flat-to-ladder transition suggests an increased influence from area 5. This means a stronger subcortical link between area 5 and motor cortex via lateral cerebellum during visually controlled stepping compared to vision-independent locomotion, a link that may be used for transmitting parietal processing of optic flow during visually guided stepping.

The head & neck- and vision-responsive groups both developed two activity peaks per cycle on the flat-to-ladder transition. These peaks were roughly in opposite phases between these two groups (Fig. 7D4,E4) and correlated with a specific gaze behavior. As described earlier, four gaze behaviors repeat twice per stride [51] Fig. 14A-D). The profile of the activity component related to visuomotor demands of ladder locomotion of visually responsive neurons resembled the frequency distribution of gaze fixations (FIX, Fig. 14E). This temporal correspondence is consistent with the suggestion that this group transmits visual information obtained during fixations. The profile of neurons with a head & neck RF coincided with or slightly preceded peaks in gaze shifts away (GSAs), and also had a trough in early swing coinciding with gaze shifts toward (GSTs; Fig. 14F,G), suggesting that the activity component of head & neck-receptive neurons that is related to visuomotor demands of the ladder reflects gaze shifts while encoding their direction (a gaze shift results from a combined movement of the head and eyes). An analogous reciprocal relationship with gaze shifts was seen in the activity of layer V 1-PEF neurons of the parietal cortex area 5 [59]. Because visual sampling is suppressed during fast gaze shifts [53], rev. in [54], for layer V 1-PEF cells of area 5, we speculated that the increased firing at the end of swing may serve as a warning of a temporary gap in visual input and facilitate processing of information obtained during constant gaze (CG) immediately after, whereas reduced activity in the first half of swing may signal another gap and disinhibit recipients in preparation for information that will be obtained during gaze fixations right after [59]. Similar suggestions can be made in respect to the head & neck-related DN group, with an addition that this group transmits such signals in a temporal correspondence with the movement of each, the right and left, limb.

Earlier, Marple-Horvat and colleagues [138] found that 50% of DN neurons responded to the displacement of a ladder crosspiece occurring at a distance, a behaviorally important visual cue requiring stride adjustment. Similar to our finding for the flat-to-ladder transition, responses included activation, inhibition, or had a complex profile. Two-thirds of those neurons also reacted to a full-field flash of light, a stronger visual stimulus that, however, did not require a motor response from the animal. In our study, only a few neurons responded to objects presented to sitting cats, however, the overwhelming majority (88%) showed vision processing-related activity during ladder locomotion. A larger fraction of visually responsive cells we saw (*p* < 0.001, χ^*2*^ test) is probably due to greater visual difference between the flat surface and ladder compared to visual stimulation provided by displacement of a single crosspiece in the study of [138].

On the flat-to-ladder transition, the activity of fore- and hindlimb-related neuronal groups diverged. PEFs of forelimb-related cells became shorter, more stride-phase specific, and their average dM tended to be greater than in the hindlimb-related groups. These differences were absent during locomotion on the flat surface, suggesting that forelimb-related adjustments were especially important for accurate ladder stepping, while hindlimb-related neurons could use forelimb network information to adjust the subsequent stride (e.g., [139]).

### Types of Visual Information Integrated with Locomotion Movement-related Signals in the Activity of Individual DN Neurons

We identified 12 modes of integration of motor and visual information in the activity of individual neurons (Fig. 9). What kind of visual information do individual neurons integrate?

**Fig. 16:**
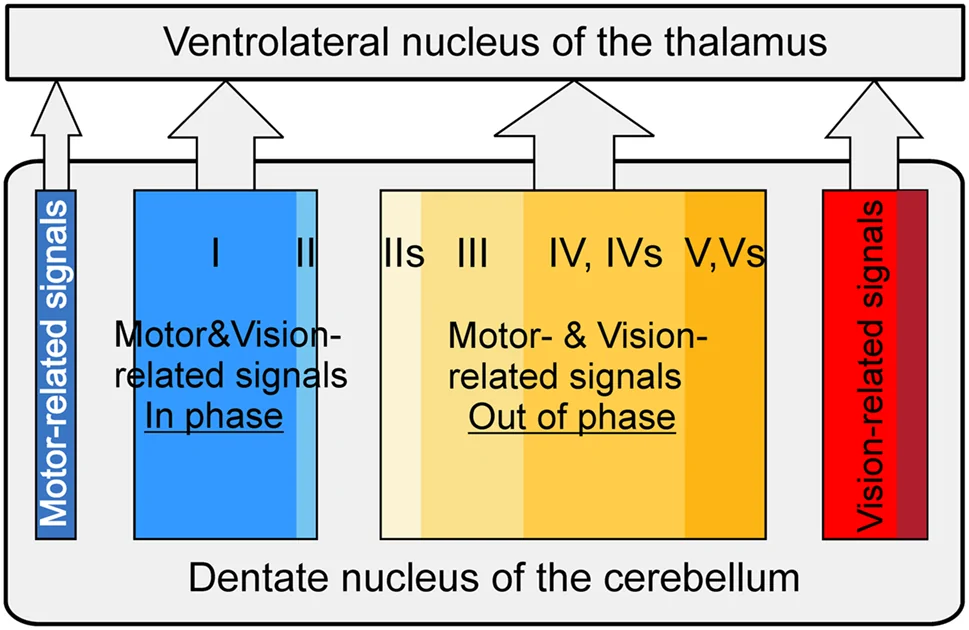
Signals from the DN to VL conveyed by individual neurons during visually guided stepping. Arrow width reflects the fraction of neurons conveying signals of the stated type. See Discussion for details

First, 13% of the DN population, neurons of *groups 0–1*, *0–2*, *1–0*, and *2–0*, only ever transmitted vision-related information during locomotion and, on the ladder, did not relay anything about the locomotion movement (Figs. 9A-D, Fig. 10, red arrow in Fig. 16). Discharges *of group 0–1* and *0–2* neurons on the ladder were stride-modulated only because visual sampling of the walking surface is stride-synchronized (see [51] for the gaze/stride synchronization). The profile of the vision processing-related activity component of *group 0–2* neurons resembled frequency distribution of gaze fixations, both peaking in mid-swing (Fig. 14H). Indeed, we previously showed that during swing of one forelimb, cats look at the future landing site for the other forelimb [51, 110] Fig. 8  A in [33]. This timing suggests that *group 0–2* neurons process visual information obtained during gaze fixations. Cells of *groups 1–0* & *2–0* (the dark red part of the red arrow in Fig. 16) likely contributed to vision-guided stepping by withdrawing from rhythmic locomotor signaling.

Most DN neurons, however, combined locomotor movement-related and vision-related information in their discharges. In 33% of the population, the firing was dominated by vision-related processes, *groups IIs*, *III*, *IVs*, & *Vs* neurons (Figs. 9E-H; Figs.11D, 12A2,C, 13C,D; orange arrow in Fig. 16). Profiles of vision processing-related activity components of all these groups aligned with gaze behaviors occurring during swing of the ipsilateral (right) forelimb (Figs. 14I,K-M,O). The vision-related component of *group Vs* activity peaked with gaze fixations (Fig. 14K), which temporal correspondence is consistent with the suggestion that this group transmits visual information obtained during gaze fixations. In contrast, vision-related components of *groups IIs and III* activity peaked with gaze shifts: *group IIs* with GSAs at swing end (Fig. 14I), *group III* with GSTs in early swing (Fig. 14M). The *group III* signal also had a negative component at the end of swing, during GSAs (Fig. 14L), and a similar negative peak was also seen in *group IVs* activity (Fig. 14O). Activation and suppression of *groups IIs, III,* & *IVs* during gaze shifts may serve functions analogous to those suggested above for head & neck-related cells.

For 16% of neurons, *groups IV* and *V*, motor- and vision-related information contributed equally to their discharges, both substantially shaping the discharge pattern (Figs. 9K, L; Figs. 13B, D; orange arrow in Fig. 16). The vision-related component of *group IV* activity coincided with GSAs at the end of swing of each forelimb (Fig. 14P), potentially signaling about gaps in visual input during GSAs similar to head & neck-related and *group IIs, III,* & *IVs* neurons.

The firing of 28% of neurons, *group I* and *II*, was dominated by the locomotion movement-related information (Figs. 9I, J; Figs. 11 C, 12A,B; light blue/cyan arrow in Fig. 16). The vision-related activity components of these groups coinciding with the peak of constant gaze (Figs. 14J,N), suggesting that these neurons process visual information obtained during constant gaze. Notably, *groups I & II* were much larger than *groups 0–2 & Vs*, whose vision-related activity coincided with gaze fixations. This is consistent with a suggestion made above that the results of constant gaze data processing are transmitted from the parietal to motor cortex via the DN rather than directly.

Finally, 5% of the population (associate members of *groups I* & *II*) always transmitted only movement-related information, even during vision-guided stepping (dark blue arrow in Fig. 16). The fraction of such neurons was significantly smaller in the DN than VL, where it was 13% (32/238, [17]* p* = 0.007, χ^*2*^ test), suggesting that pure locomotion movement-related information arrives to the VL mainly from other sources. The most viable ones are the cerebellar interposed and fastigial nuclei, and direct spino-thalamic pathways [140–142]. Indeed, the activity of most neurons in the interposed and fastigial nuclei is strongly stride-modulated on the flat surface even in decerebrate cats [55, 143–145]. Unlike DN neurons, neurons of these nuclei receive substantial direct spino-cerebellar input [100–102, 146–148], and they also give extensive projections to the VL [38, 39, 149–153]. Because interposed neurons respond rather mildly to substantial changes in walking speed or slope, i.e., to changes in the vigor of locomotor movement [144], as do motor cortex neurons [154, 155], it was concluded that during flat-surface locomotion they mainly transmit information about the spinal locomotor CPG-generated rhythm rather than peripheral apparatus [154, 156].

### DN Influence on the VL Activity During Locomotion on the Ladder

On the ladder, the activity of most DN neurons was stride-modulated, 54% (84/157) had 1-PEF and 34% (54/157) 2-PEFs. These fractions matched data from an earlier report by [28]. Unlike on the flat surface where the fraction of 2-PEF neurons was smaller in the DN than VL, on the ladder it did not differ (29% [69/238], [17] *p* = 0.256, χ^*2*^ test). This suggests greater DN influence on VL 2-PEF neurons during ladder locomotion.

As in VL, PEFs of DN neurons spanned the step cycle. Group firing of 1-PEF DN population peaked in the first half of swing (Fig. 5C4 & Fig. 15A3), earlier than on the flat surface. As on the flat surface, this did not translate into a modulated profile of the VL 1-PEF population activity, which remained rather steady (Fig. 15A4; [17]. The 2-PEF DN population increased firing, especially during the stance-to-swing transition, but the VL 2-PEF group activity also did not reflect this (Fig. 5B4 & Fig. 15B). Yet, in [17] we reported that a subset of VL 2-PEF neurons, the “stance” neurons, increased firing during swing, possibly influenced by the increased DN 2-PEF activity.

The main observation regarding the influence of DN neurons on VL cells during ladder locomotion is a considerable similarity of the changes in the firing of individual DN and VL neurons upon the flat-to-ladder transition (Figs. 8–13 and Figs. 9–12 in [17]. In the DN, we observed all the types of integration of motor and visual information (*groups I—V*) previously described in the VL [17]. Their proportions differ, however, offering some insights into the DN to VL signal transmission.

First, fewer DN than VL neurons transmit purely movement-related signals during both vision-independent and vision-guided stepping (dark blue arrow in Fig. 16*vs*. left arrow in Fig. 12 of [17]. Although a single DN neuron synapses on many VL cells [157, 158], this still suggests that the VL receives pure locomotion movement-related information from other sources as well, such as cerebellar interposed and fastigial nuclei, and direct spino-thalamic pathways as discussed above.

Second, more DN than VL neurons transmit purely vision-related signals during ladder locomotion, *groups 0–1* and *0–2* DN cells, jointly representing ~ 10% (15/157) of the DN population (red arrow in Fig. 16*vs*. right arrow in Fig. 12 of [17]. This means that the DN provides a substantial volume of pure vision-related information to the VL, which information is then integrated in the VL with locomotion movement-related information and relayed to cerebral cortex.

Third, a quarter of DN neurons belong to *groups I* or *II*, that integrate visual information into locomotion movement-related rhythm by adjusting the magnitude, salience, and/or precision of their movement-related firing without changing its phase (blue arrow in Fig. 16). In [17] we suggested that such signals may reach the VL from the cerebellum, to be then relayed to the motor cortex. Since *groups I* and *II* are more numerous in the VL than DN, additional integration of this type is likely to occur within the VL. A large fraction of neurons engaged in stable-phase integration may aid smooth locomotion on complex terrain.

Finally, many DN and VL neurons integrate visual information into the locomotion movement-related rhythm by shifting the firing phase along with other adjustments (orange arrow in Fig. 16 and middle-right arrow in Fig. 12 of [17]. This indicates that the phase adjustment is an essential element of visuo-motor integration in both cerebellar and VL thalamus processing.

These integration patterns can also be interpreted within computational frameworks of cerebellar function in which the cerebellum contributes to state estimation, forward modeling, and error-based modification of motor commands (e.g., [159–161]). From this perspective, the stride-related DN activity during flat-surface locomotion provides phase-specific information about the ongoing locomotor state, whereas the changes observed during ladder locomotion—the altered firing rate, modulation depth, phase, and temporal precision—reflect incorporation of visually derived information for foot placement into this locomotor-state signal and may contribute to adjustments required for accurate stepping. The coexistence of neurons dominated by vision-related signals, those dominated by locomotor movement-related signals, and neurons in which the two contribute comparably is consistent with a distributed dentate representation of predicted limb state, desired foot placement, and possible mismatch between them. However, our experiments did not directly measure prediction errors, distinguished predictive state signals from corrective motor commands on a trial-by-trial basis, or tested causality. Thus, the data are compatible with forward-model and error-signal-processing frameworks, but do not by themselves establish which specific computation the DN perform during visually guided stepping.

### The DN Participates in the Differential Control of Different Limb Segments

We previously showed that different forelimb segments are controlled differently by the thalamo-cortical locomotor network that includes the VL thalamus [17, 18, 20], motor compartment of the reticular nucleus of the thalamus [162, 163], primary motor cortex [93], primary somato-sensory cortex [61], and parietal cortex area 5 [59]. Data on the activity of DN neurons is consistent with the assumption that the DN participates in this differential control. During flat-surface locomotion, the activity of DN neurons related to different forelimb segments differs: the firing of the shoulder-related group peaks in the second half of swing (Figs. 6 A & 15C1), while that of the elbow-related group peaks in the second half of stance (Figs. 6B & 15D1). As noted above, these activity profiles resemble those of shoulder- and elbow-related subpopulations in the VL (Figs. 15C2, D2; [18, 20]).

Shoulder- and elbow-related DN populations also respond differently to the ladder task. The shoulder-related group decreases activity in the second half of stance, while the elbow-related one increases it (Figs. 6D4,E4; Figs. 15C3,D3). These population-level changes do not have any apparent effect on the group activity of the shoulder- and elbow-related VL groups (Figs. 15C4 *vs.* C2 & D4 *vs*. D2; [18, 20]). However, the firing of all individual DN shoulder- and elbow-related neurons changes, with about half showing a *group III-V* response dominated by visual information and half a *group I & II* response dominated by motor information while visual information adjusts the intensity, magnitude, and/or precision of their stride-related activity modulation. These changes are likely to contribute to changes in the activity of VL neurons. The fact that signaling from these DN subpopulations does not change movement of the shoulder and elbow joints on the ladder compared to flat surface (Figs. 6G2,3) indicates that these signals influence neuronal networks controlling other characteristics of locomotion, such as the specific step size.

### Limitations of the Study

A major limitation of this study is the lack of identification of axonal projections of DN neurons. It is known that the VL is one of the main targets of DN projection, however, the DN also projects to other thalamic nuclei (e.g., [9, 39, 164, 165]) and to several other brain regions (e.g., [78, 166–172]). There are interneurons in the DN as well (e.g., [173]). Thus, characterizing discharge patterns of neurons with identified axonal projections would provide a better understanding of transmission of movement- and vision-related information from the lateral cerebellum during locomotion. Also, recording in the caudal part of the DN may shed additional light on DN participation in visuomotor control during locomotion.

A second limitation is that 66% of neurons were recorded from one animal, cat 14. We believe, however, that our population-level findings are fairly robust, because they were consistent between this animal and pooled data from the other two, cats 13 and JS, who contributed 30% of data (e.g., Fig. 4A).

A third limitation is that, for the hindlimb-related neurons, we may have overestimated the fractions of cells whose peak activity occurred in different stride phases during the flat-surface and ladder locomotion (*groups IIs*, *III*, *IVs*, and *Vs*). This could happen because hindlimb stepping was inferred from the record of the ipsilateral forelimb, and the variability among strides may have reduced the accuracy of this inference, inflating estimates of phase differences between the two tasks.

Finally, we would like to note that many suggestions advanced here are based on the results of a simple averaging of discharges of neurons with dissimilar profiles of stride-phase related activity. Such averaging negates specific contribution of individual neurons. In return, it reveals the joined output of the group. For small groups, however, the phase heterogeneity among neurons may influence the population representation, thus additional data will be needed to further investigate these suggestions.

## Conclusion

We analyzed, for the first time, the firing of DN neurons in awake cats walking on a flat surface, a vision non-requiring locomotion task. Comparing this with the activity during vision-guided stepping on a horizontal ladder allowed us to describe activity components related to processing of visual information for accurate vision-guided stepping. We found such components in the activity of 88% of DN neurons. They are manifested as stride phase-specific increases and decreases in discharge, often enhancing the salience and phase precision of the signals. These signals differ by the limb segment, and many coincide with a specific gaze behavior: fixation, constant gaze episodes, or gaze shift, suggesting transmission of specific visual or gaze movement-related information. The characteristics of these target stepping-related activity components of the DN neuronal activity are well-suited to contribute to the known target stepping-related activity of neurons in the VL thalamus.

## Electronic supplementary material

Below is the link to the electronic supplementary material.

Supplementary file1 (DOCX 79 KB)

## Data Availability

The data supporting the findings of this study are available from the corresponding author (I.N.B.; ibelooz@gmail.com) upon request.

## Appendix

Some data obtained from three of the cats used in this study were included in previous publications. Data on the activity of the motor cortex during locomotion obtained from cat 8 were included in Karayannidou et al. 174, Beloozerova et al. 32, and Stout et al. 64, and data on the activity of red nucleus of this cat during postural corrections were included in Zelenin et al. 175. Data on the activity of somatosensory cortex during locomotion obtained from cat 13 were reported in Favorov et al. 61, and data on the activity of premotor cortex during locomotion of cat 14 were included in Viana Di Prisco et al. 65. In addition, data on the stabilization of paw trajectory during locomotion obtained from cat 8 were included in Klishko et al. 176, and data on the movement of the head and gaze during locomotion collected from cat 13 were reported in Rivers et al. 110 and Zubair et al. 52, 51. Finally, data from cat 14 on the strategies that cats use to avoid an obstacle on the path and data on when they need to see to step accurately on a complex surface were included in Chu et al. 177 and Volgushev et al. 33, respectively.

## Abbreviations

CV: Coefficient of variability
dM: Coefficient of stride-related activity modulation, the “depth” of modulation, characterizes the fluctuation of the probability of the discharge over the step cycle
DN: Dentate (lateral) nucleus of the cerebellum
GEE: Logistic generalized estimating equations, a statistical method for data analysis
M: Coefficient of stride-related activity modulation, characterizes the fluctuation of the discharge rate over the step cycle
PEF: Period of elevated firing
RF: Receptive field
S1: Primary somatosensory cortex, in this report refers to cytoarchitectonic areas 1 & 2
SMotor cortex: Sensory-motor cortex, in this report refers to cytoarchitectonic areas 1, 2, 4, 5, & 6
VL: Ventrolateral thalamus

## References

[1] Bastian AJ, Martin TA, Keating JG, Thach WT. Cerebellar ataxia: abnormal control of interaction torques across multiple joints. J Neurophysiol. 1996;76:492–509. 10.1152/jn.1996.76.1.492 8836239

[2] Bastian AJ, Zackowski KM, Thach WT. Cerebellar ataxia: torque deficiency or torque mismatch between joints? J Neurophysiol. 2000;83:3019–30. 10.1152/jn.2000.83.5.3019 10805697

[3] Beaubaton D, Trouche E. Participation of the cerebellar dentate nucleus in the control of a goal-directed movement in monkeys. Effects of reversible or permanent dentate lesion on the duration and accuracy of a pointing response. Exp Brain Res. 1982;46(1):127–38. 10.1007/BF00238106 7067785

[4] Botterell EH, Fulton JF. Functional localization in the cerebellum of primates III. Lesions of hemispheres (neocerebellum). J Comp Neurol. 1938;69:63–87.

[5] Flament D, Hore J. Movement and electromyographic disorders associated with cerebellar dysmetria. J Neurophysiol. 1986;55(6):1221–33. 10.1152/jn.1986.55.6.1221 3734856

[6] Holmes G. The cerebellum of man. Brain. 1939;62:1–30.

[7] Horn KM, Pong M, Gibson AR. Functional relations of cerebellar modules of the cat. J Neurosci. 2010;30(28):9411–23. 10.1523/JNEUROSCI.0440-10.2010 PMC3865504 20631170

[8] Robinson FR, Fuchs AF. The role of the cerebellum in voluntary eye movements. Annu Rev Neurosci. 2001;24:981–1004. 10.1146/annurev.neuro.24.1.981 11520925

[9] Sakayori N, Kato S, Sugawara M, Setogawa S, Fukushima H, Ishikawa R, et al. Motor skills mediated through cerebellothalamic tracts projecting to the central lateral nucleus. Mol Brain. 2019;12(1):13. 10.1186/s13041-019-0431-x PMC6368787 30736823

[10] Aoki S, Sato Y, Yanagihara D. Lesion in the lateral cerebellum specifically produces overshooting of the toe trajectory in leading forelimb during obstacle avoidance in the rat. J Neurophysiol. 2013;110(7):1511–24. 10.1152/jn.01048.2012 23615542

[11] Buckley E, Mazzà C, McNeill A. A systematic review of the gait characteristics associated with cerebellar ataxia. Gait Posture. 2018;60:154–63. 10.1016/j.gaitpost.2017.11.024 29220753

[12] Chambers WW, Sprague JM. Functional localization in the cerebellum. I. Organization in the longitudinal corticonuctear zones and their contribution to the control of posture, both extra-pyramidal and pyramidal. J Comp Neurol. 1955;103:105–29. 10.1002/cne.901030107 13263445

[13] Chambers WW, Sprague JM. Functional localization in the cerebellum II. Somatotopic organization in cortex and nuclei. Arch Neurol Psych. 1955;74:653–80. 10.1001/archneurpsyc.1955.02330180071008 13268132

[14] Glickstein M. How are visual areas of the brain connected to motor areas for the sensory guidance of movement? Trends Neurosci. 2000;23(12):613–7. 10.1016/s0166-2236(00)01681-7 11137151

[15] Glickstein M. What does the cerebellum really do? Curr Biol. 2007;17(19):R 824-827. 10.1016/j.cub.2007.08.009 17925205

[16] Stein JF, Glickstein M. Role of the cerebellum in visual guidance of movement. Physiol Rev. 1992;72:967–1017. 10.1152/physrev.1992.72.4.967 1438583

[17] Marlinski V, Nilaweera WU, Zelenin PV, Sirota MG, Beloozerova IN. Signals from the ventrolateral thalamus to the motor cortex during locomotion. J Neurophysiol. 2012;107(1):455–72. 10.1152/jn.01113.2010 PMC3349693 21994259

[18] Beloozerova IN, Stout EE, Sirota MG. Distinct thalamo-cortical controls for shoulder, elbow, and wrist during locomotion. Front Comput Neurosci. 2013;7:62. 10.3389/fncom.2013.00062 PMC3659318 23734124

[19] Beloozerova IN, Marlinski V. Contribution of the ventrolateral thalamus to the locomotion-related activity of motor cortex. J Neurophysiol. 2020;124(5):1480–504. 10.1152/jn.00253.2020 PMC7701357 32783584

[20] Beloozerova IN, Stout EE, Sirota MG. Distinct thalamo-cortical controls for shoulder, elbow, and wrist during locomotion. Front Comput Neurosci. 2013;7:62. 10.3389/fncom.2013.00062. PMC3659318 23734124

[21] Beloozerova IN, Sirota MG. The role of the motor cortex in the control of accuracy of locomotor movements in the cat. J Physiol (Lond). 1993;461:1–25. 10.1113/jphysiol.1993.sp019498 PMC1175242 8350259

[22] Den Otter AR, Geurts AC, de Haart M, Mulder T, Duysens J. Step characteristics during obstacle avoidance in hemiplegic stroke. Exp Brain Res. 2005;161:180–92. 10.1007/s00221-004-2057-0 15517222

[23] Farr TD, Liu L, Colwell KL, Whishaw IQ, Metz GA. Bilateral alteration in stepping pattern after unilateral motor cortex injury: a new test strategy for analysis of skilled limb movements in neurological mouse models. J Neurosci Meth. 2006;153:104–13. 10.1016/j.jneumeth.2005.10.011 16309746

[24] Friel KM, Drew T, Martin JH. Differential activity-dependent development of corticospinal control of movement and final limb position during visually guided locomotion. J Neurophysiol. 2007;97:3396–406. 10.1152/jn.00750.2006 PMC2740651 17376849

[25] Liddell EGT, Phillips CG. Pyramidal section in the cat. Brain. 1944;67:1–9.

[26] Metz GA, Whishaw IQ. Cortical and subcortical lesions impair skilled walking in the ladder rung walking test: a new task to evaluate fore- and hindlimb stepping, placing, and co-ordination. J Neurosci Methods. 2002;115:169–79. 10.1016/s0165-0270(02)00012-2 11992668

[27] Trendelenburg W. Untersuchungen uber reizlose vorubergehende Aussaltung am Zentralnervensystem. III. Die extermitaten Region der Grosshirninde. Pflugers Arch. 1911;137:515–44.

[28] Marple-Horvat DE, Criado JM. Rhythmic neuronal activity in the lateral cerebellum of the cat during visually guided stepping. J Physiol (Lond). 1999;518(2):595–603. 10.1111/j.1469-7793.1999.0595p.x PMC2269445 10381604

[29] Beloozerova IN, Sirota MG. The role of motor cortex in control of locomotion. In: Gurfincel VS, Ioffe ME, Massion J, Roll JP, editors. Stance and motion. Facts and concepts. New York: Plenum Press; 1988. p. 163–76.

[30] Beloozerova IN, Sirota MG, Swadlow HA. Activity of different classes of neurons of the motor cortex during locomotion. J Neurosci. 2003;23(3):1087–97. 10.1523/JNEUROSCI.23-03-01087.2003 PMC6741911 12574439

[31] Beloozerova IN, Sirota MG, Swadlow HA, Orlovsky GN, Popova LB, Deliagina TG. Activity of different classes of neurons of the motor cortex during postural corrections. J Neurosci. 2003;23(21):7844–53. 10.1523/JNEUROSCI.23-21-07844.2003 PMC6740594 12944514

[32] Beloozerova IN, Farrell BJ, Sirota MG, Prilutsky BI. Differences in movement mechanics, electromyographic, and motor cortex activity between accurate and non-accurate stepping. J Neurophysiol. 2010;103:2285–300. 10.1152/jn.00360.2009 PMC2853277 20164404

[33] Volgushev M, Nguyen CT, Iyer GS, Beloozerova IN. When cats need to see to step accurately? J Physiol (Lond). 2022;600(1):75–94. 10.1113/JP282255 PMC9241584 34761816

[34] Pryor K. Lads before the wind. New York: Harper and Row; 1975.

[35] Skinner BF. The behavior of organisms. New York: Appleton-Century-Crofts Inc; 1938.

[36] de Carvalho WD, Rosalino LM, Dalponte JC, Santos B, Harumi Adania C, Lustosa Esbérard CE. Can footprints of small and medium sized felids be distinguished in the field? Evidences from Brazil’s Atlantic Forest. Trop Conserv Sci. 2015;8(3):760–77.

[37] Prilutsky BI, Sirota MG, Gregor RJ, Beloozerova IN. Quantification of motor cortex activity and full-body biomechanics during unconstrained locomotion. J Neurophysiol. 2005;94(4):2959–69. 10.1152/jn.00704.2004 15888524

[38] Evrard HC, Craig AD. Retrograde analysis of the cerebellar projections to the posteroventral part of the ventral lateral thalamic nucleus in the macaque monkey. J Comp Neurol. 2008;508:286–314. 10.1002/cne.21674 18322920

[39] Nakano K, Takimoto T, Kayahara T, Takeuchi Y, Kobayashi Y. Distribution of cerebellothalamic neurons projecting to the ventral nuclei of the thalamus: an HRP study in the cat. J Comp Neurol. 1980;194:427–39. 10.1002/cne.901940209 7440809

[40] Efron B, Tibshirani RJ. An introduction to the bootstrap. New York: Chapman & Hall; 1993.

[41] Stout EE, Beloozerova IN. Differential responses of fast- and slow-conducting pyramidal tract neurons to changes in accuracy demands during locomotion. J Physiol. 2013;591(2647–2666):2013. 10.1113/jphysiol.2012.232538 PMC3678048 23381901

[42] Batschelet E. Circular statistics in biology. London: Academic; 1981.

[43] Drew T, Doucet S. Application of circular statistics to the study of neuronal discharge during locomotion. J Neurosci Methods. 1991;38:171–81. 10.1016/0165-0270(91)90167-x 1784121

[44] Fisher NI. Statistical analysis of circular data. Cambridge: Cambridge University Press; 1993.

[45] Reinoso-Suarez F. Topographischer Hirnatlas der Katze für experimental physiologische Untersuchungen. Darmstadt, Germany: E. Merck; 1961.

[46] Snider RS, Niemer WT. A stereotaxic atlas of the cat brain. Chicago and London: University of Chicago Press; 1961.

[47] Berman AL. The brain stem of the cat: a cytoarchitectonic atlas with stereotaxic coordinates. Madison: University of Wisconsin Press; 1968.

[48] Berntson GG, Paulucci TS, Torello MW. An atlas of the deep cerebellar nuclei and subtentorial brainstem of the cat with compensation for skull-size. Brain Res Bull. 1978;3(5):475–92. 10.1016/0361-9230(78)90077-1 122713

[49] Hoover JE, Strick PL. The organization of cerebellar and basal ganglia outputs to primary motor cortex as revealed by retrograde transneuronal transport of herpes simplex virus type 1. J Neurosci. 1999;19(4):1446–63. 10.1523/JNEUROSCI.19-04-01446.1999 PMC6786031 9952421

[50] Beloozerova IN, Sirota MG. Integration of motor and visual information in the parietal area 5 during locomotion. J Neurophysiol. 2003;90(2):961–71. 10.1152/jn.01147.2002 12904498

[51] Zubair HN, Chu KMI, Johnson JL, Rivers TJ, Beloozerova IN. Gaze coordination with strides during walking in the cat. J Physiol (Lond). 2019;597(21):5195–229. 10.1113/JP278108 PMC9260858 31460673

[52] Zubair HN, Beloozerova IN, Sun H, Marlinski V. Head movement during walking in the cat. Neuroscience. 2016;332:101–20. 10.1016/j.neuroscience.2016.06.031 PMC4986613 27339731

[53] Bridgeman B, Hendry D, Stark L. Failure to detect displacement of the visual world during saccadic eye movements. Vision Res. 1975;15:719–22. 10.1016/0042-6989(75)90290-4 1138489

[54] Wurtz RH. Neuronal mechanisms of visual stability. Vision Res. 2008;48:2070–89. 10.1016/j.visres.2008.03.021 PMC2556215 18513781

[55] Schwartz AB, Ebner TJ, Bloedel JR. Responses of interposed and dentate neurons to perturbations of the locomotor cycle. Exp Brain Res. 1987;67:323–38. 10.1007/BF00248553 3622692

[56] Beloozerova IN, Sirota MG. Cortically controlled gait adjustments in the cat. Ann N Y Acad Sci. 1998;860:550–3. 10.1111/j.1749-6632.1998.tb09101.x 9928360

[57] Nilaweera WU, Beloozerova IN. Neurons of lateral nucleus of cerebellum change their step-related frequency modulation when visual control of stepping is required. Program No 367.25. In: Neuroscience meeting planner. Chicago, IL: Society for Neuroscience, 2009. [Online]

[58] Andujar JE, Lajoie K, Drew T. A contribution of area 5 of the posterior parietal cortex to the planning of visually guided locomotion: limb-specific and limb-independent effects. J Neurophysiol. 2010;103(2):986–1006. 10.1152/jn.00912.2009 20018828

[59] Beloozerova IN, Nilaweera WU, Viana Di Prisco G, Marlinski V. Signals from posterior parietal area 5 to motor cortex during locomotion. Cereb Cortex. 2023;33(4):1014–43. 10.1093/cercor/bhac118 PMC9930630 35383368

[60] Drew T. Motor cortical activity during voluntary gait modifications in the cat. I. Cells related to the forelimbs. J Neurophysiol. 1993;70(1):179–99. 10.1152/jn.1993.70.1.179 8360715

[61] Favorov OV, Nilaweera WU, Miasnikov AA, Beloozerova IN. Activity of somatosensory-responsive neurons in high subdivisions of SI cortex during locomotion. J Neurosci. 2015;35(20):7763–76. 10.1523/JNEUROSCI.3545-14.2015 PMC4438126 25995465

[62] Fortier-Lebel N, Nakajima T, Drew T. Two premotor areas (6aα and 6aγ) involved in motor preparation and execution during visually guided locomotion in the cat. Cereb Cortex. 2025;35(7):bhaf159. 10.1093/cercor/bhaf159 PMC12315547 40748337

[63] Nakajima T, Fortier-Lebel N, Drew T. Premotor cortex provides a substrate for the temporal transformation of information during the planning of gait modifications. Cereb Cortex. 2019;29(12):4982–5008. 10.1093/cercor/bhz039 PMC6918931 30877802

[64] Stout EE, Sirota MG, Beloozerova IN. Known and unexpected constraints evoke different kinematic, muscle, and motor cortical neuron responses during locomotion. Eur J Neurosci. 2015;42(9):2666–77. 10.1111/ejn.13053 PMC4626377 26302230

[65] Viana Di Prisco G, Marlinski V, Beloozerova IN. Activity of cat premotor cortex neurons during visually guided stepping. J Neurophysiol. 2023;130(4):838–60. 10.1152/jn.00114.2023 PMC10642938 37609687

[66] Widajewicz W, Kably B, Drew T. Motor cortical activity during voluntary gait modifications in the cat. II. Cells related to the hindlimbs. J Neurophysiol. 1994;72(5):2070–89. 10.1152/jn.1994.72.5.2070 7884445

[67] Brodal P. The corticopontine projection in the cat. I. Demonstration of a somatotopically organized projection from the primary sensorimotor cortex. Exp Brain Res. 1968;5(3):210–34. 5721752

[68] Brodal P. The corticopontine projection in the cat. I. The projection from the proreate gyrus. J Comp Neurol. 1971;142(2):127–39. 10.1002/cne.901420202 4106737

[69] Brodal P. The corticopontine projection in the cat. II. The projection from the orbital gyrus. J Comp Neurol. 1971;142(2):141–51. 10.1002/cne.901420203 4106738

[70] Allen GI, Tsukahara N. Cerebrocerebellar communication systems. Physiol Rev. 1974;54(4):957–1006. 10.1152/physrev.1974.54.4.957 4370744

[71] Broch-Smith T, Brodal P. Organization of the cortico-ponto-cerebellar pathway to the dorsal paraflocculus. An experimental study with anterograde and retrograde transport of WGA-HRP in the cat. Arch Ital Biol. 1990;128(2–4):249–71. 1702610

[72] Brodal P, Bjaalie JG. Organization of the pontine nuclei. Neurosci Res. 1992;13(2):83–118. 10.1016/0168-0102(92)90092-q 1374872

[73] Shinoda Y, Sugiuchi Y, Futami T. Excitatory inputs to cerebellar dentate nucleus neurons from the cerebral cortex in the cat. Exp Brain Res. 1987;67:299–315. 10.1007/BF00248551 3040457

[74] Shinoda Y, Sugiuchi Y, Futami T, Izawa R. Axon collaterals of mossy fibers from the pontine nucleus in the cerebellar dentate nucleus. J Neurophysiol. 1992;67(3):547–60. 10.1152/jn.1992.67.3.547 1578244

[75] Yoshikazu S, Yuriko S, Takahiro F. Organization of excitatory inputs from the cerebral cortex to the cerebellar dentate nucleus. Can J Neurol Sci. 1993;20(S3):S19–28. 8334589

[76] Shinoda Y, Izawa Y, Sugiuchi Y, Futami T. Functional significance of excitatory projections from the precerebellar nuclei to interpositus and dentate nucleus neurons for mediating motor, premotor and parietal cortical inputs. Prog Brain Res. 1997;114:193–207. 10.1016/s0079-6123(08)63365-8 9193145

[77] Gerrits NM, Voogd J. The projection of the nucleus reticularis tegmenti pontis and adjacent regions of the pontine nuclei to the central cerebellar nuclei in the cat. J Comp Neurol. 1987;258:52–69. 10.1002/cne.902580104 3571536

[78] Gibson AR, Horn KM, Pong M. Nucleus reticularis tegmenti pontis: a bridge between the basal ganglia and cerebellum for movement control. Exp Brain Res. 2023;241(5):1271–87. 10.1007/s00221-023-06574-0 PMC10129968 37000205

[79] Giolli RA, Gregory KM, Suzuki DA, Blanks RH, Lui F, Betelak KF. Cortical and subcortical afferents to the nucleus reticularis tegmenti pontis and basal pontine nuclei in the macaque monkey. Vis Neurosci. 2001;18:725–40. 10.1017/s0952523801185068 11925008

[80] Edley SM, Graybiel AM. The afferent and efferent connections of the feline nucleus tegmenti pedunculopontinus, pars compacta. J Comp Neurol. 1983;217:187–215. 10.1002/cne.902170207 6886052

[81] Mori F, Okada KI, Nomura T, Kobayashi Y. The pedunculopontine tegmental nucleus as a motor and cognitive interface between the cerebellum and basal ganglia. Front Neuroanat. 2016;10:109. 10.3389/fnana.2016.00109 PMC5097925 27872585

[82] Kunzle H. Bilateral projections from precentral motor cortex to the putamen and other parts of the basal ganglia: an autoradiographic study in *Macaca fascicularis*. Brain Res. 1975;88:195–209. 10.1016/0006-8993(75)90384-4 50112

[83] Nambu A. A new dynamic model of the cortico-basal ganglia loop. Prog Brain Res. 2004;143:461–6. 10.1016/S0079-6123(03)43043-4 14653188

[84] Nambu A. Somatotopic organization of the primate basal ganglia. Front Neuroanat. 2011;5:26. 10.3389/fnana.2011.00026 PMC3082737 21541304

[85] Ragsdale CW Jr, Graybiel A. The fronto-striatal projection in the cat and monkey and its relationship to inhomogeneities established by acetylcholinesterase histochemistry. Brain Res. 1981;208:259–66. 10.1016/0006-8993(81)90556-4 6163506

[86] Selemon LD, Goldman-Rakic PS. Longitudinal topography and interdigitation of corticostriatal projections in the rhesus monkey. J Neurosci. 1985;5:776–94. 10.1523/JNEUROSCI.05-03-00776.1985 PMC6565017 2983048

[87] Yeterian E, Pandya DN. Striatal connections of the parietal cortices in rhesus monkeys. J Comp Neurol. 1993;332:175–97. 10.1002/cne.903320204 8331211

[88] Mullié Y, Arto I, Yahiaoui N, Drew T. Contribution of the entopeduncular nucleus and the globus pallidus to the control of locomotion and visually guided gait modifications in the cat. Cereb Cortex. 2020;30(9):5121–46. 10.1093/cercor/bhaa106 PMC7391415 32377665

[89] Sales-Carbonell C, Taouali W, Khalki L, Pasquet MO, Petit LF, Moreau T, et al. No discrete start/stop signals in the dorsal striatum of mice performing a learned action. Curr Biol. 2018;28:3044-3055.e3045. 10.1016/j.cub.2018.07.038 PMC6231539 30270180

[90] Schwarz M, Sontag KH, Wand P. Sensory-motor processing in substantia nigra pars reticulata in conscious cats. J Physiol (Lond). 1984;347:129–47. 10.1113/jphysiol.1984.sp015057 PMC1199438 6707952

[91] Shi LH, Luo F, Woodward DJ, Chang JY. Neural responses in multiple basal ganglia regions during spontaneous and treadmill locomotion in rats. Exp Brain Res. 2004;157:314. 10.1007/s00221-004-1844-y 15067433

[92] West MO, Carelli RM, Pomerantz M, Cohen SM, Gardner JP, Chapin JK, et al. A region in the dorsolateral striatum of the rat exhibiting single-unit correlations with specific locomotor limb movements. J Neurophysiol. 1990;64:1233–46. 10.1152/jn.1990.64.4.1233 2258744

[93] Stout EE, Beloozerova IN. Pyramidal tract neurons receptive to different forelimb joints act differently during locomotion. J Neurophysiol. 2012;107(7):1890–903. 10.1152/jn.00650.2011 PMC3331672 22236716

[94] Graham Brown BT. The intrinsic factors in the act of progression in the mammal. Proc Biol Sci. 1911;84:308–19. 10.1098/rspb.1911.0077.

[95] Grillner S, Zangger P. On the central generation of locomotion in the low spinal cat. Exp Brain Res. 1979;34:241–61. 10.1007/BF00235671 421750

[96] Forssberg H, Grillner S, Halbertsma J. The locomotion of the low spinal cat. I. Coordination within a hindlimb. Acta Physiol Scand. 1980;108:269–81. 10.1111/j.1748-1716.1980.tb06533.x 7376922

[97] Forssberg H, Grillner S, Halbertsma J, Rossignol S. The locomotion of the low spinal cat. II. Interlimb coordination. Acta Physiol Scand. 1980;108(283–295):1980. 10.1111/j.1748-1716.1980.tb06534.x 7376923

[98] Rybak IA, Shevtsova NA, Lafreniere-Roula M, McCrea DA. Modelling spinal circuitry involved in locomotor pattern generation: insights from deletions during fictive locomotion. J Physiol (Lond). 2006;577:617–39. 10.1113/jphysiol.2006.118703 PMC1890439 17008376

[99] Grillner S, Kozlov A. The CPGs for limbed locomotion – facts and fiction. Int J Mol Sci. 2021;22(11):5882. 10.3390/ijms22115882 PMC8198624 34070932

[100] Arshavsky YI, Gelfand IM, Orlovsky GN. Cerebellum and rhythmical movements. Heidelberg: Springer Berlin; 1986.

[101] Bloedel JR, Courville J. Cerebellar afferent systems. In: Brooks VB, editor. *Handbook of physiology*, section I, vol. 2, part 2. Bethesda: American Physiological Society; 1981. p. 735–830.

[102] Stecina K, Fedirchuk B, Hultborn H. Information to cerebellum on spinal motor networks mediated by the dorsal spinocerebellar tract. J Physiol. 2013;591(22):5433–43. 10.1113/jphysiol.2012.249110 PMC3853486 23613538

[103] Bantli H, Bloedel JR. Spinal input to the lateral cerebellum mediated by infratentorial structures. Neuroscience. 1977;2(4):555–68. 10.1016/0306-4522(77)90051-3 199856

[104] Asanuma H, Stoney SD Jr, Abzug C. Relationship between afferent input and motor outflow in cat motorsensory cortex. J Neurophysiol. 1968;31:670–81. 10.1152/jn.1968.31.5.670 5711138

[105] Murphy JT, Wong YC, Kwan HC. Afferent-efferent linkages in motor cortex for single forelimb muscles. J Neurophysiol. 1975;38:990–1014. 10.1152/jn.1975.38.4.990 125786

[106] Rosen I, Asanuma H. Peripheral afferent input to the forelimb area of the monkey motor cortex: input-output relations. Exp Brain Res. 1972;14:257–73. 10.1007/BF00816162 4626361

[107] Sakata H, Miyamoto J. Topographic relationship between the receptive fields of neurons in the motor cortex and the movements elicited by focal stimulation in freely moving cats. Jpn J Physiol. 1968;18:489–507. 10.2170/jjphysiol.18.489 5304340

[108] Flierman NA, Koay SA, van Hoogstraten WS, Ruigrok TJH, Roelfsema P, Badura AI, et al. Encoding of cerebellar dentate neuron activity during visual attention in rhesus macaques. Elife. 2025;13:RP99696. 10.7554/eLife.99696 PMC11737872 39819496

[109] Hollands MA, Marple-Horvat DE. Visually guided stepping under conditions of step cycle-related denial of visual information. Exp Brain Res. 1996;109:343–56. 10.1007/BF00231792 8738381

[110] Rivers TJ, Sirota MG, Guttentag AI, Ogorodnikov DA, Shah NA, Beloozerova IN. Gaze shifts and fixations dominate gaze behavior of walking cats. Neuroscience. 2014;275:477–99. 10.1016/j.neuroscience.2014.06.034 PMC4169884 24973656

[111] Matthis JS, Yates JL, Hayhoe MM. Gaze and the control of foot placement when walking in natural terrain. Curr Biol. 2018;28:1224-1233.e5. 10.1016/j.cub.2018.03.008 PMC5937949 29657116

[112] Brodal P. The corticopontine projection from the visual cortex in the cat. I. The total projection and the projection from area 17. Brain Res. 1972;39(2):297–317. 10.1016/0006-8993(72)90438-6 4113019

[113] Brodal P. The corticopontine projection from the visual cortex in the cat. II. The projection from areas 18 and 19. Brain Res. 1972;39(2):319–35. 10.1016/0006-8993(72)90439-8 4113020

[114] Gibson A, Baker J, Mower G, Glickstein M. Corticopontine cells in area 18 of the cat. J Neurophysiol. 1978;41(2):484–95. 10.1152/jn.1978.41.2.484 650278

[115] Sanides D, Fries W, Albus K. The corticopontine projection from the visual cortex of the cat. An autoradiographic investigation. J Comp Neurol. 1978;179:49–76. 10.1002/cne.901790106 8980718

[116] Glickstein M, Cohen JL, Dixon B, Gibson A, Hollins M, Labossiere E, et al. Corticopontine visual projections in macaque monkeys. J Comp Neurol. 1980;190:209–29. 10.1002/cne.901900202 7381057

[117] Glickstein M, May JG 3rd, Mercier BE. Corticopontine projections in the macaque: the distribution of labelled cortical cells after large injections of horseradish peroxidase in the pontine nuclei. J Comp Neurol. 1985;235:343–59. 10.1002/cne.902350306 3998215

[118] May PJ, Warren S, Kojima Y. The superior colliculus projection upon the macaque inferior olive. Brain Struct Funct. 2024;229(8):1855–71. 10.1007/s00429-023-02743-7 PMC11792976 38240754

[119] Mower G, Gibson A, Glickstein M. Tectopontine pathways in the cat: laminar distribution of cells of origin and visual properties of target cells in dorsolateral pontine nucleus. J Neurophysiol. 1979;42:1–15. 10.1152/jn.1979.42.1.1 85698

[120] Graybiel A. Visuo-cerebellar and cerebellar-visual connections involving the ventral lateral geniculate nucleus. Exp Brain Res. 1974;20:303–6. 10.1007/BF00238320 4426354

[121] Baker J, Gibson A, Glickstein M, Stein J. Visual cells in the pontine nuclei of the cat. J Physiol (Lond). 1976;225:414–33. 10.1113/jphysiol.1976.sp011287 PMC1309255 1255527

[122] Mower G, Gibson A, Robinson F, Stein J, Glickstein M. Visual pontocerebellar projections in the cat. J Neurophysiol. 1980;43:355–65. 10.1152/jn.1980.43.2.355 7381525

[123] Suzuki DA, May JG, Keller EL, Yee RD. Visual motion-producing properties of neurones in dorsolateral pontine nucleus of alert monkey. J Neurophysiol. 1990;63:37–59. 10.1152/jn.1990.63.1.37 2299385

[124] Thier P, Koehler W, Buettner UW. Neuronal activity in the dorsolateral pontine nuclei of the alert monkey modulated by visual stimuli and eye movements. Exp Brain Res. 1988;70:496–512. 10.1007/BF00247598 3384051

[125] Burne RA, Mihailoff GA, Woodward DJ. Visual corticopontine input to the paraflocculus: a combined autoradiographic and horseradish peroxidase study. Brain Res. 1978;143:139–46. 10.1016/0006-8993(78)90757-6 630397

[126] Kralj-Hans I, Baizer JS, Swales C, Glickstein M. Independent roles for the dorsal paraflocculus and vermal lobule VII of the cerebellum in visuomotor coordination. Exp Brain Res. 2007;177(2):209–22. 10.1007/s00221-006-0661-x 16951960

[127] Robinson FR, Cohen JL, May J, Sestokas AK, Glickstein M. Cerebellar targets of visual pontine cells in the cat. J Comp Neurol. 1984;223(4):471–82. 10.1002/cne.902230402 6325507

[128] Fushiki H, Sato Y, Miura A, Kawasaki T. Climbing fiber responses of Purkinje cells to retinal image movement in cat cerebellar flocculus. J Neurophysiol. 1994;71(4):1336–50. 10.1152/jn.1994.71.4.1336 8035218

[129] Gellman R, Houk JC, Gibson AR. Somatosensory properties of the inferior olive of the cat. J Comp Neurol. 1983;215(2):228–43. 10.1002/cne.902150210 6853776

[130] Marigold DS, Drew T. Posterior parietal cortex estimates the relationship between object and body location during locomotion. Elife. 2017;6:e28143. 10.7554/eLife.28143 PMC5650472 29053442

[131] Fowler GA, Sherk HA. Gaze during visually-guided locomotion in cats. Behav Brain Res. 2003;139(1–2):83–96. 10.1016/s0166-4328(02)00096-7 12642179

[132] Patla AE, Vickers JN. Where and when do we look as we approach and step over an obstacle in the travel path? NeuroReport. 1997;8(17):3661–5. 10.1097/00001756-199712010-00002 9427347

[133] Patla AE, Vickers JN. How far ahead do we look when required to step on specific locations in the travel path during locomotion? Exp Brain Res. 2003;148(1):133–8. 10.1007/s00221-002-1246-y 12478404

[134] Gibson JJ. Visually controlled locomotion and visual orientation in animals. Br J Psychol. 1958;49(3):182–94. 10.1111/j.2044-8295.1958.tb00656.x 13572790

[135] Lee DN. The optic flow field: the foundation of vision. Philos Trans R Soc Lond B Biol Sci. 1980;290(1038):169–79. 10.1098/rstb.1980.0089 6106236

[136] Sherk H, Fowler GA. Visual analysis and image motion in locomoting cats. Eur J Neurosci. 2001;13(6):1239–48. 10.1046/j.0953-816x.2001.01493.x 11285021

[137] Sun HJ, Carey DP, Goodale MA. A mammalian model of optic flow utilization in the control of locomotion. Exp Brain Res. 1992;91(1):171–5. 10.1007/BF00230026 1301371

[138] Marple-Horvat DE, Criado JM, Armstrong DM. Neuronal activity in the lateral cerebellum of the cat related to visual stimuli at rest, visually guided step modification, and saccadic eye movements. J Physiol (Lond). 1998;506(Pt 2):489–514. 10.1111/j.1469-7793.1998.489bw.x PMC2230718 9490874

[139] McVea DA, Pearson KG. Long-lasting memories of obstacles guide leg movements in the walking cat. J Neurosci. 2006;26:1175–8. 10.1523/JNEUROSCI.4458-05.2006 PMC6674580 16436604

[140] Craig AD. Retrograde analyses of spinothalamic projections in the macaque monkey: input to the ventral lateral nucleus. J Comp Neurol. 2008;508:315–28. 10.1002/cne.21672 18322921

[141] Mackel R, Iriki A, Brink EE. Spinal input to thalamic VL neurons: evidence for direct spinothalamic effects. J Neurophysiol. 1992;67:132–44. 10.1152/jn.1992.67.1.132 1552315

[142] Yen CT, Honda CN, Jones EG. Electrophysiological study of spinothalamic inputs to ventrolateral and adjacent thalamic nuclei of the cat. J Neurophysiol. 1991;66:1033–47. 10.1152/jn.1991.66.3.1033 1753274

[143] Armstrong DM, Edgley SA. Discharges of nucleus interpositus neurons during locomotion in the cat. J Physiol (Lond). 1984;351:411–32. 10.1113/jphysiol.1984.sp015253 PMC1193125 6747870

[144] Armstrong DM, Edgley SA. Discharges of interpositus and Purkinje cells of the cat cerebellum during locomotion under different conditions. J Physiol (Lond). 1988;400:425–45. 10.1113/jphysiol.1988.sp017130 PMC1191817 3418533

[145] Orlovsky GN. Work of the neurons of the cerebellar nuclei during locomotion. Biophysics. 1972;17:1177–85.

[146] Eccles JC, Rosén I, Scheid P, Táboríková H. Patterns of convergence onto interpositus neurons from peripheral afferents. J Neurophysiol. 1974;37(6):1438–48. 10.1152/jn.1974.37.6.1438 4436712

[147] Matsushita M, Ikeda M. Spinal projections to the cerebellar nuclei in the cat. Exp Brain Res. 1970;10(5):501–11. 10.1007/BF00234266 4194263

[148] Eccles JC, Sabah NH, Táboríková H. The pathways responsible for excitation and inhibition of fastigial neurones. Exp Brain Res. 1974;19(1):78–99. 10.1007/BF00233396 4360384

[149] Angaut P, Guilbaud G, Reymond MC. An electrophysiological study of the cerebellar projections to the nucleus ventralis lateralis of thalamus in the cat. I. Nuclei fastigii et inerpositus. J Comp Neurol. 1968;134(1):9–20. 10.1002/cne.901340103 5712414

[150] Angaut P. The cerebello-thalamic projection in the cat. In: Interactions C-C, editor. Massion J and Sasaki K. Amsterdam: Elsevier North-Holland; 1979. p. 1943.

[151] Ilinsky IA, Kultas-Ilinsky K. An autoradiographic study of topographical relationships between pallidal and cerebellar projections to the cat thalamus. Exp Brain Res. 1984;54:95–106. 10.1007/BF00235822 6698151

[152] Rinvik E, Grofová I. Cerebellar projections to the nuclei ventralis lateralis and ventralis anterior thalami. Experimental electron microscopical and light microscopical studies in the cat. Anat Embryol (Berl). 1974;146:95–111. 10.1007/BF00341384 4618725

[153] Rispal-Padel L, Grangetto A. The cerebello-thalamo-cortical pathway. Topographical investigation at the unitary level in the cat. Exp Brain Res. 1977;28:101–23. 10.1007/BF00237089 880997

[154] Armstrong DM, Drew T. Discharges of pyramidal tract and other motor cortical neurones during locomotion in the cat. J Physiol (Lond). 1984;346:471–95. 10.1113/jphysiol.1984.sp015036 PMC1199513 6699782

[155] Beloozerova IN, Sirota MG. The role of the motor cortex in the control of vigour of locomotor movements in the cat. J Physiol (Lond). 1993;461:27–46. 10.1113/jphysiol.1993.sp019499 PMC1175243 8350266

[156] Orlovsky GN, Deliagina TG, Grillner S. Neuronal control of locomotion. From mollusc to man. New York: Oxford Univ. Press; 1999.

[157] Auman TD, Horne MK. Ramification and termination of single axons in the cerebellothalamic pathway of the rat. J Comp Neurol. 1996;376:420–30. 10.1002/(SICI)1096-9861(19961216)376:3<420::AID-CNE5>3.0.CO;2-4 8956108

[158] Mason A, Ilinsky IA, Maldonado S, Kultas-Ilinsky K. Thalamic terminal fields of individual axons from the ventral part of the dentate nucleus of the cerebellum in *Macaca mulatta*. J Comp Neurol. 2000;421(3):412–28. 10813796

[159] Bastian AJ. Learning to predict the future: the cerebellum adapts feedforward movement control. Curr Opin Neurobiol. 2006;16(6):645–9. 10.1016/j.conb.2006.08.016 17071073

[160] Kakei S, Bostan AC, Ebner TJ, Fakharian MA, Gomi H, Guell X, et al. Consensus paper: models of cerebellar functions. Cerebellum. 2026;25(1):15. 10.1007/s12311-025-01939-3 PMC12886374 41661363

[161] Popa LS, Ebner TJ. Cerebellum, Predictions and Errors. Front Cell Neurosci. 2019;12:524. 10.3389/fncel.2018.00524 PMC6340992 30697149

[162] Marlinski V, Beloozerova IN. Burst firing of neurons in the thalamic reticular nucleus during locomotion. J Neurophysiol. 2014;112(1):181–92. 10.1152/jn.00366.2013 PMC4064390 24740856

[163] Marlinski V, Sirota MG, Beloozerova IN. Differential gating of thalamocortical signals by reticular nucleus of thalamus during locomotion. J Neurosci. 2012;32(45):15823–36. 10.1523/JNEUROSCI.0782-12.2012 PMC3505038 23136421

[164] Melik-Musyan AB, Fanardjyan VV. Projections of the central cerebellar nuclei to the intralaminar thalamic nuclei in cats. Neurophysiol. 1998;30:39–47. 10.1007/BF02463111.

[165] Nakamura H. Cerebellar projections to the ventral lateral geniculate nucleus and the thalamic reticular nucleus in the cat. J Neurosci Res. 2018;96(1):63–74. 10.1002/jnr.24105 28639357

[166] Asanuma C, Thach WT, Jones EG. Distribution of cerebellar terminations and their relation to other afferent terminations in the ventral lateral thalamic region of the monkey. Brain Res Rev. 1983;5:237–65. 10.1016/0165-0173(83)90015-2 6189561

[167] Asanuma C, Thach WT, Jones EG. Brainstem and spinal projections of the deep cerebellar nuclei in the monkey, with observations in the brainstem projections of the dorsal column nuclei. Brain Res Rev. 1983;5:299–322. 10.1016/0165-0173(83)90017-6 6189563

[168] Bantli H, Bloedel JR. Monosynaptic activation of a direct reticulo-spinal pathway by the dentate nucleus. Pflugers Arch. 1975;357(3–4):237–42. 10.1007/BF00585978 1238988

[169] Novello M, Bosman LWJ, De Zeeuw CI. A systematic review of direct outputs from the cerebellum to the brainstem and diencephalon in mammals. Cerebellum. 2024;23(1):210–39. 10.1007/s12311-022-01499-w PMC10864519 36575348

[170] Pong M, Horn KM, Gibson AR. Pathways for control of face and neck musculature by the basal ganglia and cerebellum. Brain Res Rev. 2008;58(2):249–64. 10.1016/j.brainresrev.2007.11.006 18199482

[171] Sugimoto T, Mizuno N, Uchida K. Distribution of cerebellar fiber terminals in the midbrain visuomotor areas: an autoradiographic study in the cat. Brain Res. 1982;238(2):353–70. 10.1016/0006-8993(82)90110-x 7093658

[172] Tolbert DL, Bantli H, Bloedel JR. Multiple branching of cerebellar efferent projections in cats. Exp Brain Res. 1978;31(3):305–16. 10.1007/BF00237291 648599

[173] Chan-Palay V. Neuronal circuitry in the nucleus lateralis of the cerebellum. Z Anat Entwicklungsgesch. 1973;142(3):259–65. 10.1007/BF00519132 4361150

[174] Karayannidou A, Beloozerova IN, Zelenin PV, Stout EE, Sirota MG, Orlovsky GN, et al. Activity of pyramidal tract neurons in the cat during standing and walking on an inclined plane. J Physiol (Lond). 2009;587(Pt 15):3795–811. 10.1113/jphysiol.2009.170183 PMC2746611 19491244

[175] Zelenin PV, Beloozerova IN, Sirota MG, Orlovsky GN, Deliagina TG. Activity of red nucleus neurons in the cat during postural corrections. J Neurosci. 2010;30(43):14533–42. 10.1523/JNEUROSCI.2991-10.2010 PMC2988228 20980611

[176] Klishko AN, Farrell BJ, Beloozerova IN, Latash ML, Prilutsky BI. Stabilization of cat paw trajectory during locomotion. J Neurophysiol. 2014;112(6):1376–91. 10.1152/jn.00663.2013 PMC4137248 24899676

[177] Chu MI, Seto SH, Beloozerova IN, Marlinski V. Strategies for obstacle avoidance during walking in the cat. J Neurophysiol. 2017;118(2):817–31. 10.1152/jn.00033.2017 PMC5539443 28356468

