# Supplementary material for: Integration of Motor and Visual Information in the Dentate (lateral) Nucleus of Cerebellum During Locomotion
Source: Cerebellum. 2026 Oct 24;25(6):161. doi: 10.1007/s12311-026-02088-x (PMC13695671; doi:10.1007/s12311-026-02088-x)
Supplement: Supplementary file 1 [file 12311_2026_2088_MOESM1_ESM.docx]

**Supplementary Figure 1.** **Example responses of neurons to passive somatosensory or visual stimulation, and the firing during standing.**

**A:** Neuron #649 activates in response to passive flexion of the right shoulder.

**B:** Neuron #638 activates in response to a visual stimulus moving in front of the animal from the right to the left.

**C:** Neuron #531 firing during standing.
